# Adverse perinatal outcomes associated with antiretroviral therapy in women living with HIV: A systematic review and meta-analysis

**DOI:** 10.3389/fmed.2022.924593

**Published:** 2023-02-03

**Authors:** Clara Portwood, Harriet Sexton, Mary Kumarendran, Zoe Brandon, Shona Kirtley, Joris Hemelaar

**Affiliations:** ^1^National Perinatal Epidemiology Unit, Nuffield Department of Population Health, University of Oxford, Oxford, United Kingdom; ^2^Centre for Statistics in Medicine, Nuffield Department of Orthopaedics, Rheumatology, and Musculoskeletal Sciences, University of Oxford, Oxford, United Kingdom

**Keywords:** HIV, perinatal, pregnancy, antiretroviral, preterm (birth)

## Abstract

**Background:**

Maternal HIV infection is associated with an increased risk of adverse perinatal outcomes. The World Health Organization (WHO) recommends immediate initiation of lifelong antiretroviral therapy (ART) for all people living with HIV, including pregnant women living with HIV (WLHIV). We aimed to assess the risk of adverse perinatal outcomes in WLHIV receiving ART compared to ART-naïve WLHIV and HIV-negative women.

**Materials and methods:**

We conducted a systematic literature review by searching PubMed, CINAHL, Global Health, and EMBASE for studies published between Jan 1, 1980, and April 20, 2020. Two investigators independently selected relevant studies and extracted data from studies reporting on the association of pregnant WLHIV receiving ART with adverse perinatal outcomes. Perinatal outcomes examined were preterm birth (PTB), very PTB, spontaneous PTB (sPTB), low birth weight (LBW), very LBW (VLBW), term LBW, preterm LBW, small for gestational age (SGA), very SGA (VSGA), stillbirth, and neonatal death. Random-effects meta-analyses examined the risk of adverse perinatal outcomes in WLHIV receiving ART compared to ART-naïve WLHIV and HIV-negative women. Subgroup and sensitivity analyses were performed based on country income status and study quality, and adjustment for confounding factors assessed.

**Results:**

Of 94,594 studies identified, 73 cohort studies, including 424,277 pregnant women, met the inclusion criteria. We found that WLHIV receiving ART are associated with a significantly decreased risk of PTB (relative risk 0.79, 95% CI 0.67–0.93), sPTB (0.46, 0.32–0.66), LBW (0.86, 0.79–0.93), and VLBW (0.62, 0.39–0.97) compared to ART-naïve WLHIV. However, WLHIV receiving ART are associated with a significantly increased risk of PTB (1.42, 1.28–1.57), sPTB (2.20, 1.32–3.67), LBW (1.58, 1.36–1.84), term LBW (1.88, 1.23–2.85), SGA (1.69, 1.32–2.17), and VSGA (1.22, 1.10–1.34) compared to HIV-negative women.

**Conclusion:**

ART reduces the risk of adverse perinatal outcomes in pregnant WLHIV, but the risk remains higher than in HIV-negative women. Our findings support the WHO recommendation of immediate initiation of lifelong ART for all people living with HIV, including pregnant WLHIV.

**Systematic review registration:**

https://www.crd.york.ac.uk/prospero/, identifier CRD42021248987.

## Introduction

37.7 million people globally were living with HIV in 2020, of whom 19.3 million are women over the age of 15 (1). An estimated 1.3 million women living with HIV (WLHIV) are pregnant each year, the vast majority residing in sub-Saharan Africa. This population is increasing, with women and girls accounting for 59% of new HIV infections in sub-Saharan Africa, a region that also has the highest neonatal and child mortality rates (2).

Pregnancies in WLHIV without antiretroviral therapy (ART) are associated with an increased risk of preterm birth (PTB), low birthweight (LBW), small for gestational age (SGA), and stillbirth, compared to HIV-negative women (3). PTB is the leading cause of neonatal and child mortality globally, with an estimated 14.8 million preterm births occurring each year (4). 23.3 million infants born SGA contribute to 21.9% of neonatal deaths in low- and middle-income countries (LMICs) (5). Both PTB and SGA contribute to the 18 million infants born annually with LBW (6), a perinatal outcome commonly used in LMICs, as gestational age at birth is often unknown.

ART is crucial for WLHIV to improve maternal health and to reduce perinatal HIV transmission. In the past, World Health Organization (WHO) guidelines included combination ART (cART) for pregnant WLHIV who required treatment for their own health, whereas zidovudine (ZDV) monotherapy was recommended for prevention of perinatal HIV transmission. From 2013, WHO recommended that all pregnant WLHIV should receive cART during pregnancy (7). This was updated in 2015 to a recommendation that all people living with HIV should initiate lifelong cART as soon as possible after diagnosis, irrespective of CD4 count, including pregnant WLHIV (8). As a result, the proportion of pregnant WLHIV receiving ART increased from 44 to 82% during 2010–2018. Whether ART use in pregnancy is associated with an increased risk of adverse perinatal outcomes has been controversial. A number of studies suggest adverse perinatal outcomes are associated with ART exposure during pregnancy, with conflicting results regarding regimen complexity, drug classes, and timing of ART initiation (9–14).

The United Nations’ Sustainable Development Goal 3 (SDG3) target 3.2 aims to end preventable deaths of new-borns and children under 5 years of age by 2030 and reduce neonatal and under-5 mortality to 12 and 25 per 1,000 live births, respectively (15). As the number of pregnant WLHIV receiving ART increases, a better understanding of the association of ART with perinatal outcomes is crucial. It is uncertain whether ART improves perinatal outcomes in WLHIV, and whether ART restores the risk of adverse perinatal outcomes to a level comparable with HIV-negative women. We conducted a systematic review and meta-analysis to examine the risk 11 specific perinatal outcomes in WLHIV receiving ART compared to WLHIV without ART and HIV-negative women.

## Materials and methods

### Search strategy

The systematic review and meta-analyses were conducted based on a protocol developed according to the Cochrane guidelines and registered online (PROSPERO, number CRD42021248987). Electronic literature databases PubMed, CINAHL (Ebscohost), Global Health (Ovid), EMBASE (Ovid) were searched for studies published between Jan 1, 1980, and April 20, 2020 using a comprehensive search strategy adapted for each database, developed by a specialist librarian (SK). Both free text and controlled vocabulary search terms for “pregnancy outcome,” “specific perinatal outcomes,” “HIV,” and “antiretroviral therapy” were used. No methodological, country, or language filters were applied, and both full-text articles and abstracts were considered. The full search terms can be found in Supplementary Appendix 1. Retrieved citations were imported into EndNote reference manager (EndNote X9; Clarivate Analytics, Philadelphia, PA, USA) and deduplicated.

### Study selection and eligibility criteria

Studies that contained information on the association of pregnant WLHIV receiving ART with adverse perinatal outcomes were eligible. The titles and abstracts of citations retrieved by the literature searches were reviewed and full text manuscripts of selected citations were obtained and assessed against the eligibility criteria by at least two independent investigators (CP, HS, MK, and ZB). Inclusion criteria were study design (prospective and retrospective cohort studies), population (pregnant women), exposure (WLHIV with ART exposure) and comparators (WLHIV without ART exposure or HIV-negative women). ART exposure was defined as any number, class, and combination of antiretroviral drugs received during pregnancy. cART exposure was defined as exposure to ≥ 3 antiretroviral drugs. WLHIV were not considered to have been exposed to ART if they only received a single ART dose at delivery or received antenatal ART for < 30 days. Studies were not included if less than 95% of women in an exposure or comparator group conformed to the exposure/comparator definition (e.g., < 95% of WLHIV received ART) or if additional treatment was received by one exposure/comparator group only. Perinatal outcomes of interest were defined as follows: preterm birth (PTB, birth < 37^+0^ weeks gestation); (16) very PTB (VPTB, birth < 32^+0^ weeks gestation); (16) spontaneous PTB (sPTB, birth following spontaneous onset of labor < 37^+0^ weeks gestation); low birthweight (LBW, < 2,500 g); (6) very LBW (VLBW, < 1,500 g); (6) small for gestational age (SGA, birthweight for gestational age < 10^th^ centile); (17) very SGA (VSGA, birthweight for gestational age < 3^rd^ centile), (17) stillbirth (delivery of an infant without any signs of life with birthweight ≥ 1,000 g or gestational age ≥ 24^+0^ weeks or body length ≥ 35 cm); (18) and neonatal death (NND, death of an infant in the first 28 days of life) (18). Term and preterm LBW were defined according to definitions of PTB and LBW. Perinatal outcome data were not included if outcomes were not defined or if defined differently from our definitions. If a cohort was reported more than once, the study containing the most recent and complete data was included. If studies reported different perinatal outcomes for the same cohort, each study was included. References of included studies were assessed for additional relevant studies. Details of excluded papers are available upon request. Any ambiguities or disagreements regarding inclusion of studies were resolved through discussion with the senior investigator (JH).

### Data extraction

Data on study and population characteristics, HIV/ART exposures and perinatal outcomes were independently extracted from eligible studies by at least two investigators (CP, HS, MK, and ZB) and reviewed by the senior investigator (JH). Outcome data according to HIV/ART exposure were extracted. Information on methods to adjust for confounders, including regression analysis (i.e., confounders corrected for), risk factor analysis (i.e., risk factors not significantly different between groups), and matching was extracted. Reported unadjusted and adjusted relative risks (RR), odds ratios (OR), and 95% confidence intervals (CIs) of perinatal outcomes according to HIV/ART exposure were also extracted.

### Quality assessment

The quality of individual studies was assessed using an adapted Newcastle-Ottawa Scale by at least two investigators (CP, HS, MK, and ZB) and reviewed by the senior investigator (JH). Nine criteria were assessed in three groups: Selection of study participants (maximum 4 points), Comparability of comparator groups (maximum 2 points), and Assessment of outcomes of interest, including methods to assess gestational age at birth (maximum 3 points). Studies were defined as “good,” “average,” or “poor” quality according to predefined criteria (Supplementary Appendix 2).

### Statistical analysis

Perinatal outcomes were compared between WLHIV receiving ART and either WLHIV without ART or HIV-negative women. Dichotomous outcome data according to HIV/ART exposure from individual studies were used to generate RRs and 95% CIs. Pairwise meta-analyses were carried out if two or more studies reported data for the same perinatal outcome for WLHIV receiving ART as well as WLHIV without ART or HIV-negative women. For all meta-analyses, a random-effects model was used to calculate a weighted summary effect estimate (RR) and 95% CI. Meta-analyses were represented in forest plots and the *I*^2^ statistic was used to quantify heterogeneity due to clinical and methodological variability between studies. The degree of heterogeneity was classified as none (< 25%), low (25–49%), moderate (50–74%), or high (≥ 75%). Prescribed subgroup analyses were carried out to assess the effects of country income status and sensitivity analyses were done to investigate whether study quality and the adjustment for confounders had an impact on the associations between HIV/ART exposure and perinatal outcomes. The Peters’ test was used to assess publication bias in meta-analyses containing ten or more studies. All statistical analyses were done with Stata version 13 (College Station, TX, USA). The systematic review is reported according to the Preferred Reporting Items for Systematic Reviews and Meta-Analyses (PRISMA) guidelines.

## Results

**FIGURE 1:**
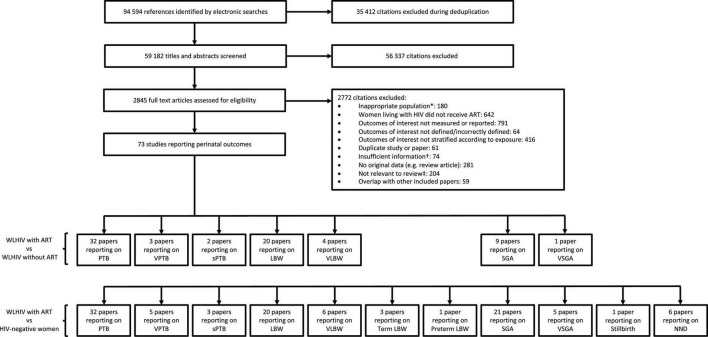
Study selection. *For example, women living with HIV were not pregnant. †For example, paper did not provide relevant outcome data. ‡For example, Assisted Reproductive Technology. ART, antiretroviral therapy; HIV, human immunodeficiency virus; LBW, low birthweight; NND, neonatal death; PTB, preterm birth; SGA, small for gestational age; sPTB, spontaneous preterm birth; VLBW, very low birthweight; VPTB, very preterm birth; VSGA, very small for gestational age; WLHIV, women living with HIV. See Materials and methods for definitions of perinatal outcomes.

The literature search yielded 94,594 citations, of which 73 studies reported relevant data (Figure 1). The perinatal outcomes reported for WLHIV receiving ART compared to WLHIV without ART were PTB (32 studies), VPTB (3), sPTB (2), LBW (20), VLBW (4), SGA (9), and VSGA (1) (Figure 1). The perinatal outcomes reported for WLHIV receiving ART compared to HIV-negative women were PTB (32 studies), VPTB (5), sPTB (3), LBW (20), VLBW (6), term LBW (3), preterm LBW (1), SGA (21), VSGA (5), stillbirth (1), and NND (6) (Figure 1).

Characteristics of included studies are summarized in Table 1 (10, 19–89). 33 prospective (45%) and 40 retrospective (55%) cohort studies analyzed data from 424,277 women in 27 countries (Table 1). 36 studies (49%) with 64,778 women took place in high income countries (HICs), and 37 studies (51%) with 359,499 women took place in low- and middle-income countries (LMICs). 50 studies (68%) reported the methods used to determine gestational age, with six (8%) studies exclusively using, or confirming gestational age with, first trimester ultrasound, the most accurate method of establishing gestational age (12). 38 studies (52%) used last normal menstrual period (LNMP), 27 studies (37%) used second trimester or unspecified ultrasound, 12 studies (16%) used symphysis-fundal height measurements, and six studies (8%) used Ballard score to determine gestational age. Two studies (3%) used an unspecified “clinical method” to determine gestational age. 35 studies (48%) reported using > 1 method to determine gestational age. 23 (32%) studies did not report methods used to determine gestational age. 57 studies (78%) used methods to assess potential confounding factors. Regression analysis was conducted in 28 studies, risk factor analysis was carried out in 45 studies, and matching of participants was carried out in eight studies (Supplementary Appendix 2.4). Of the 41 comparisons which were adjusted for covariates in individual studies, only six resulted in a change of the effect estimate from significant to no significant difference in adverse perinatal outcomes between groups (Supplementary Appendix 4). Quality assessments classified 32 studies (44%) as poor quality, 40 (55%) as average quality and one (2%) as good quality (Table 1 and Supplementary Appendix 2.3). Studies from LMICs had quality ratings (3% good, 54% average, and 43% poor quality) comparable to studies from HICs (55% average, 44% poor quality).

**TABLE 1:** Characteristics of studies included in the systematic review and meta-analysis.

References	Country	Country Income Status	Cohort study design	Recruitment period	Number of women analyzed	Population characteristics*	Method to correct for confounders	Method to estimate gestational age	Quality assessment
Adam et al. (19)	Sudan	Middle	Retrospective	1/2009 to 12/2013	78	Women recruited from maternity hospital	Risk factor analysis	No description	Average
Ai-Jie and Yong-zhong (20)	China	Middle	Retrospective	1/2006 to 3/2008	155	Twins excluded, rural and urban setting	None	No description	Poor
Albert et al. (21)	Canada	High	Retrospective	1/1/1997 to 31/1/2018	477	Twins excluded, women recruited from a provincial surveillance database, 46.1% smoking, 23.3% alcohol use, 26.0% IDU	Risk factor analysis	Ultrasound in first and/or second trimester	Average
Azria et al. (22)	France	High	Retrospective	1/2003 to 6/2007	300	Twins excluded, women recruited from a level III maternity unit, urban setting, hospital deliveries, 4.3% smoking during pregnancy, 1.7% history of IDU	Risk factor analysis, matching	First day of LNMP, corrected if needed by routine first trimester ultrasound	Average
Bailey et al. (23)	Ukraine	Middle	Retrospective	2008 to 2010	3535	First born twin included, hospital deliveries, 14.7% history of IDU	None	LNMP and ultrasound (unspecified)	Poor
Balogun et al. (24)	Canada	High	Prospective	9/2010 to 12/2015	104	Twins excluded, women recruited from 4 sites in Toronto, 0% smoking	Risk factor analysis, matching	LNMP confirmed by ultrasound (unspecified)	Average
Bengtson et al. (25)	South Africa	Middle	Prospective	3/2013 to 8/2015	1116	Twins excluded, women recruited from antenatal care clinics in Gugulethu Cape Town, urban setting, 17.2% alcohol use	None	Ultrasound (unspecified), LNMP, or symphysis-fundal height	Poor
Boer et al. (26)	Netherlands	High	Retrospective	12/1997 to 7/2003	294	First born twin included, women recruited from an academic medical centre, 12.9% smoking, 1.7% history of IDU	Regression analysis, matching	LNMP confirmed by first trimester ultrasound	Poor
Boyajian et al. (27)	Canada	High	Retrospective	1/1/2003 to 10/1/2010	364	Second twin excluded, women recruited from tertiary pregnancy referral centre, hospital deliveries, 6.3% smokers, 1.4% IDU	Regression analysis, risk factor analysis, matching	No description	Average
Carceller et al. (28)	Canada	High	Retrospective	1997 to 2005	412	Recruited from a tertiary hospital in Montreal, urban setting, hospital deliveries	None	No description	Poor
Chagomerana et al. (29)	Malawi	Low	Retrospective	1/4/2012 to 15/11/2015	3074	Twins excluded, urban setting, hospital deliveries	Regression analysis	LNMP	Average
Chen et al. (30)	Botswana	Middle	Retrospective	1/5/2009 to 30/4/2011	33148	First born twin included, hospital deliveries, 5.3% alcohol use, 1.7% smoking	Regression analysis, risk factor analysis	LNMP, symphysis-fundal height, or ultrasound (unspecified)	Average
Chibwesha et al. (31)	Zambia	Low	Retrospective	1/2/2006 to 31/12/2012	200557	First born twin included, women recruited from MNCH health system, urban setting	None	LNMP and symphysis-fundal height	Poor
Cooper et al. (32)	USA	High	Prospective	1/1990 to 6/2000	1542	Twins excluded, 31% IDU	Risk factor analysis	LNMP, ultrasound (unspecified), symphysis-fundal height, or neonatal assessment (unspecified)	Poor
Cotter et al. (33)	USA	High	Prospective	1/1990 to 12/2002	1337	Twins excluded, 5.4% alcohol use, 11.2% smoking, 17.8% IDU, women recruited from medical centre, hospital deliveries	Regression analysis, risk factor analysis	LNMP and/or ultrasound (unspecified)	Poor
Dadabhai et al. (34)	Malawi	Low	Prospective	1/2016 to 9/2017	1299	Twins excluded, 96% of deliveries occurred in healthcare facilities, urban setting	Regression analysis	Ballard score and LNMP	Average
De Souza et al. (35)	USA	High	Retrospective	1/1/1990 to 31/12/1994	403	First born twin included, women recruited from a tertiary hospital, 18.9% IDU	Risk factor analysis	No description	Average
Djeha et al. (36)	Canada	High	Prospective	1/2003 to 12/2016	159	Urban setting, 9.4% smoking	None	First trimester ultrasound or LNMP	Average
Duryea et al. (37)	USA	High	Retrospective	1/1984 to 4/2014	1004	Twins excluded, women recruited from hospital, hospital deliveries	Regression analysis, risk factor analysis	No description	Average
European Collaborative Study (38)	Belgium, Denmark, Germany, Italy, Netherlands, Poland, Spain, Sweden United Kingdom	High	Prospective	1985 to 12/2001	2414	Women recruited from medical centres, 19.6% history of or current IDU	Regression analysis, risk factor analysis	LNMP or ultrasound (unspecified)	Average
Gagnon et al. (39)	Canada	High	Retrospective	1/2007 to 31/2012	384	Twins excluded, women recruited from tertiary referral centre, urban setting, hospital deliveries, 6% smoking, 1% alcohol use, 2% IDU	Regression analysis, risk factor analysis	First trimester ultrasound or conception date by assisted reproduction if available	Average
Garcia-Otero et al. (40)	Spain	High	Prospective	12/2014 to 3/2017	94	Women recruited from hospital and hospital clinic, urban setting, 20.2% smoking, 3.2% IDU	Risk factor analysis	No description	Average
Gibango et al. (41)	South Africa	Middle	Prospective	4/2012 to 10/2012	496	Twins excluded, women recruited from a tertiary academic hospital, urban setting, hospital deliveries	None	Ballard score	Poor
Goetghebuer et al. (42)	Belgium	High	Prospective	12/2010 to 11/2013	255	Women recruited from hospital antenatal clinic, urban setting, 9.2% smoking, 10.1% alcohol use	Risk factor analysis	Ballard score	Average
Gonzales et al. (43)	Mozambique	Low	Prospective	3/2010 to 4/2012	1744	Semi-rural setting	Risk factor analysis	Ballard score, symphysis-fundal height	Average
Habib et al. (44)	Tanzania	Low	Retrospective	1999 to 2006	5870	Twins excluded, women recruited from an electronic birth registry, hospital deliveries	Regression analysis	LNMP	Average
Haeri et al. (45)	USA	High	Retrospective	1/2000 to 1/2007	453	Women recruited from 2 tertiary care centres, 13.3% smoking	Regression analysis, risk factor analysis, matching	LNMP and ultrasound (unspecified)	Average
Hernandez et al. (46)	Spain	High	Prospective	6/2006 to 12/2007	56	Twins excluded, women recruited from materno-fetal medicine department of hospital, urban setting, 25% smoking, 0% alcohol use, 0% IDU	Risk factor analysis, matching	No description	Average
Hofer et al. (47)	Brazil	Middle	Prospective	1996 to 2010	588	Twins excluded, women recruited from tertiary care centre, urban setting	Risk factor analysis	No description	Average
Hu et al. (48)	China	Middle	Prospective	10/2009 to 5/2018	802	Twins included, urban setting	Regression analysis, risk factor analysis	First or second trimester ultrasound, in the absence of ultrasound LNMP used	Average
Joseph et al. (49)	Nigeria	Middle	Retrospective	1/2008 to 6/2009	249	Twins excluded, women recruited from a tertiary referral centre, hospital deliveries	Risk factor analysis	No description	Average
Jumare et al. (50)	Nigeria	Middle	Prospective	2013 to 2017	424	Twins included, women recruited from a specialist hospital, urban setting	Risk factor analysis	LNMP	Average
Kakkar et al. (51)	Canada	High	Prospective	1988 to 2011	589	Twins excluded, women recruited from a tertiary referral centre and the largest maternal-health centre in the province	Regression analysis, risk factor analysis	LNMP and ultrasound (unspecified)	Average
Kowalska et al. (52)	Poland	Middle	Prospective	1/1995 to 2/2003	102	Twins included, women recruited from an outpatient HIV clinic, 47.1% IDU	Risk factor analysis	LNMP	Poor
Li et al. (10)	Tanzania	Low	Prospective	11/2004 to 9/2011	3314	Women recruited from hospitals, health centres and dispensaries, urban setting	Risk factor analysis	LNMP and symphysis-fundal height	Poor
Li et al. (53)	China	Middle	Prospective	10/2014 to 9/2017	1449	Twins excluded, women recruited from midwifery hospitals	Regression analysis, risk factor analysis	LNMP or ultrasound (unspecified)	Average
Liff et al. (54)	Botswana	Middle	Prospective	4/2016 to 4/2017	179	Twins excluded, women recruited from 8 nationwide delivery sites	Risk factor analysis	Second trimester ultrasound	Poor
Lopez et al. (55)	Spain	High	Retrospective	1/1986 to 6/2010	1557	Twins excluded, women recruited from a tertiary hospital, urban setting, hospital deliveries, 55.2% smoking	Regression analysis, risk factor analysis, matching	Second trimester ultrasound	Poor
Malaba et al. (56)	South Africa	Middle	Prospective	4/2013 to 8/2015	1793	Twins excluded, recruited from large community primary care facility, urban setting	Regression analysis, risk factor analysis	LNMP and symphysis-fundal height	Average
Malaba et al. (57)	South Africa	Middle	Prospective	4/2014 to 10/2016	1787	Twins excluded, women recruited from a large primary care antenatal clinic, urban setting	Regression analysis	LNMP and symphysis-fundal height	Average
Mandelbrot et al. (58)	France	High	Retrospective	1/9/1985 to 31/12/1996	2834	Twins excluded, 31% IDU, recruited from obstetrical services, hospital deliveries	None	LNMP, confirmed by first trimester ultrasound	Poor
Marazzi et al. (59)	Malawi and Mozambique	Low	Retrospective	7/2005 to 6/2009	3273	Twins included, women recruited from DREAM centres	Regression analysis	LNMP and clinical exam (unspecified)	Average
Marti et al. (60)	Spain	High	Prospective	1/1/1997 to 31/12/2003	167	Twins excluded, women recruited from hospital, hospital deliveries, urban setting, 1% IDU	None	No description	Poor
Matheson et al. (61)	USA	High	Prospective	3/1986 to 12/1993	321	Twins excluded, 41.7% IDU	Risk factor analysis	Ballard score	Average
Mehta et al. (62)	South Africa	Middle	Retrospective	7/10/2013 to 6/10/2014	10293	Twins included, women recruited from hospital, urban setting, hospital deliveries, 0.09% smoking, 0.2% alcohol use, 0.04% IDU	Risk factor analysis	LNMP, ultrasound (unspecified)	Average
Moodley et al. (63)	South Africa	Middle	Retrospective	7/2011 to 12/2011, 1/2014 to 6/2014	9847	Twins excluded, data abstracted from maternity registers of a regional hospital	Regression analysis, risk factor analysis	LNMP and/or ultrasound (unspecified)	Average
Moseholm et al. (64)	Denmark	High	Retrospective	1/1/2000 to 31/12/2016	2980	Twins excluded, women recruited from specialised clinical centres for treatment and care of pregnant women living with HIV, 7.6% smoking during pregnancy	Risk factor analysis, matching	No description	Average
Olagbuji et al. (65)	Nigeria	Middle	Prospective	1/2007 to 12/2008	406	Twins excluded, women recruited from a tertiary referral centre, all delivered in a healthcare facility	Risk factor analysis	No description	Poor
Orloff et al. (66)	USA	High	Retrospective	1/7/1994 to 30/6/1998	927	Twins included, urban setting, 46.5% smoking, 45.8% alcohol use, 42.7% IDU	None	No description	Poor
Phiri et al. (67)	USA	High	Retrospective	1/1/1994 to 31/12/2009	790	6.7% alcohol use, 25.0% smoking, 11.0% IDU	Regression analysis	LNMP, ultrasound (unspecified), and clinical assessment	Poor
Ramokolo et al. (68)	South Africa	Middle	Retrospective	10/2012 to 5/2013	8778	Women recruited from primary health facilities	Risk factor analysis	LNMP	Average
Rempis et al. (69)	Uganda	Low	Retrospective	2/2013 to 12/2013	412	Twins excluded, all deliveries in a private referral hospital	Risk factor analysis	No description	Poor
Rudin et al. (70)	Switzerland	High	Prospective	1984 to 2007	1040	Twins excluded, 22% smoking, 26% IDU	None	No description	Poor
Santosa et al. (71)	South Africa	Middle	Prospective	28/5/2013 to 20/7/2016	633	Twins excluded, women recruited from hospital, 98.7% hospital deliveries, urban setting, 6.4% smoking, 8.2% alcohol	Regression analysis, risk factor analysis	Ultrasound <14 weeks	Good
Saums et al. (72)	USA	High	Retrospective	2011 to 2018	3729	Women recruited from hospital, urban setting, hospital deliveries, 11.5% smoking, 2.9% alcohol use, 13.4% IDU	Risk factor analysis	No description	Average
Schulte et al. (73)	USA	High	Retrospective	1989 to 2004	11231	27.6% history of IDU	Regression analysis	LNMP, ultrasound (unspecified), neonatal assessment (unspecified)	Poor
Sebitloane and Moodley (74)	South Africa	Middle	Retrospective	1/4/2011 to 30/4/2014	1461	Twins excluded, women recruited at a regional hospital, urban setting, hospital deliveries	None	No description	Poor
Short et al. (75)	United Kingdom	High	Retrospective	1996 to 2010	331	Twins included, women recruited from a HIV antenatal clinic, urban setting, deliveries in a tertiary hospital,13.0% smoking	None	No description	Poor
Silverman (76)	Zambia	Low	Retrospective	Unspecified	1238	Twins included	Risk factor analysis	No description	Poor
Simonds et al. (77)	USA	High	Retrospective	1985 to 12/1995	1366	Twins excluded, 18.4% IDU	None	Ballard score	Poor
Snijdewind et al. (78)	Netherlands	High	Retrospective	1/1997 to 2/2015	10795	Twins excluded, women recruited from 26 nationwide sites, 10.8% smoking, 11.7% alcohol use, 0.6% IDU	Risk factor analysis	Early ultrasound or LNMP	Average
Tiam et al. (79)	Lesotho	Middle	Prospective	6/2014 to 2/2016	1594	Women recruited from 14 mixed setting study centres across 3 districts, 91.6% delivered in a health facility	None	LNMP	Poor
Townsend ECS (80)	Belgium, Denmark, Germany, Italy, Netherlands, Poland, Spain, Sweden United Kingdom	High	Prospective	1990 to 2006	4253	Twins excluded, 35.4% IDU	Regression analysis	LNMP and/or ultrasound (unspecified)	Poor
Townsend NSHPC (80)	United Kingdom, Ireland	High	Prospective	1990 to 2006	6426	Women recruited from 205 hospitals across UK and Ireland, 4.4% IDU	Regression analysis	No description	Poor
Tuomala et al. (81)	USA	High	Retrospective	1/1/1990 to 1998	3266	Twins excluded, women recruited from PACTS and WITS studies, and 3 single site studies, 39.9% tobacco use during pregnancy, 26.9% alcohol use during pregnancy, 28.7% IDU use during pregnancy	Regression analysis, risk factor analysis	LNMP and/or ultrasound (unspecified), or neonatal assessment (unspecified)	Average
Van der Merwe et al. (82)	South Africa	Middle	Retrospective	10/2004 to 3/2007	1630	Twins excluded, women recruited from HIV referral centres including a tertiary hospital, 3.7% smoking, 3.9% alcohol use	Regression analysis, risk factor analysis	LNMP, ultrasound (unspecified), symphysis-fundal height, neonatal assessment (unspecified)	Poor
Von Linstow et al. (83)	Denmark	High	Retrospective	1/6/1994 to 30/6/2008	255	Twins included, women recruited from 6 centres nationwide, all hospital deliveries, 15.4% smoking, 2.2% IDU	None	Late ultrasound at 18-20 weeks	Poor
Watts et al. (84)	USA and Puerto Rico	High	Retrospective	2007 to 31/10/2010	1869	Twins excluded, 17% smoking,17% smoking, 8.0% alcohol use, 8.0% IDU	Regression analysis	Clinical method (unspecified) and ultrasound (unspecified)	Average
Wedderburn et al. (85)	South Africa	Middle	Prospective	5/3/2012 to 31/3/2015	732	Women recruited from 2 community based antenatal care clinics, peri-urban setting, 35% smoker, 14.5% alcohol	Risk factor analysis	Ultrasound (unspecified), LNMP and symphysis-fundal height	Average
Wilkinson et al. (86)	Tanzania	Low	Prospective	3/2012 to 11/2012	100	Twins excluded	Risk factor analysis	LNMP, or symphysis-fundal height	Average
Yu et al. (87)	China	Middle	Retrospective	6/2006 to 7/2010	194	Twins excluded, 8.8% IDU	Risk factor analysis	No description	Poor
Zash et al. (88)	Botswana	Middle	Retrospective	15/8/2014 to 15/8/2016	57005	Twins excluded, women recruited from 8 government hospitals, hospital deliveries, 8.3% alcohol or smoking in pregnancy	Regression analysis	LNMP and/or ultrasound (unspecified), or symphysis-fundal height	Average
Ziske et al. (89)	Tanzania	Low	Prospective	9/2008 to 9/2009	144	Twins excluded, women recruited from antenatal care (HIV+ receiving ART) or maternity ward (HIV+ no ART), rural setting, hospital deliveries	Risk factor analysis	No description	Poor

*Details on the inclusion of twins, recruitment centre, urban/rural setting, deliveries at home/hospital, smoking, alcohol use, and IDU were sought and reported here if provided by each study. ART, antiretroviral therapy; DREAM, Determined, Resilient, Empowered, AIDS-free, Mentored and Safe; ECS, European Collaborative Study; HIV, human immunodeficiency virus; HIV+, HIV positive; IDU, illicit drug use; LNMP, last normal menstrual period; MNCH, Maternal, New-born, and Child Health; NSHPC, National Study of HIV in Pregnancy and Childhood; PACTS, Perinatal AIDS Collaborative Transmission Studies; WITS, Women and Infants Transmission Study.

The ART regimens taken by WLHIV receiving ART, exposure comparisons reported, and perinatal outcomes analyzed are displayed for each study in Table 2. 41 studies (56%) reported perinatal outcomes in WLHIV receiving ART compared to WLHIV without ART, and 38 studies (52%) compared perinatal outcomes in WLHIV receiving ART with HIV-negative women. Six studies (8%) reported on both comparisons. In 32 (44%) studies ≥ 95% of women received cART in the group of WLHIV who received ART. Only five studies (7%) included WLHIV solely exposed to ZDV monotherapy. The remaining 36 studies (49%) reported on WLHIV receiving a mixture of different ART regimens (Table 2).

**TABLE 2:** Antiretroviral therapies, HIV/ART comparisons, and perinatal outcomes reported by studies included in the systematic review and meta-analysis.

References	ART regimens	WLHIV with ART vs. WLHIV without ART	WLHIV with ART vs. HIV-negative women	Perinatal outcomes
Adam et al. (19)	ZDV-3TC dual therapy, cART (proportions/drug class(es) unspecified)	No	Yes	PTB
Ai-Jie and Yong-zhong (20)	77.4% ZDV monotherapy, 22.6% NNRTI-based cART (ZDV-3TC-NVP)	Yes	No	LBW
Albert et al. (21)	4.5% mono/dual/triple NRTI therapy, 17.7% NNRTI-based cART, 73.7% PI-based cART, 4.1% INSTI-based cART	Yes	No	sPTB
Azria et al. (22)	PI-based cART (LPV/r)	No	Yes	PTB, VPTB, SGA, VSGA, NND
Bailey et al. (23)	91.3% ZDV monotherapy, 1.2% dual therapy, 7.5% cART (91.0% PI-based cART)	Yes	No	PTB
Balogun et al. (24)	PI-based cART (50.7% LPV/r, 31.8% ATV/r, 4.8% DRV/r)	No	Yes	sPTB, SGA
Bengtson et al. (25)	NNRTI-based cART (TDF-FTC/3TC-EFV)	No	Yes	PTB, SGA, VSGA
Boer et al. (26)	PI-/NNRTI-based cART (proportions unspecified)	No	Yes	PTB, LBW, VLBW
Boyajian et al. (27)	75.0% PI-based cART, 25.0% non-PI based cART	No	Yes	PTB, LBW, SGA
Carceller et al. (28)	85.4% PI-based cART, 14.6% non-PI based cART	No	Yes	PTB, Term LBW
Chagomerana et al. (29)	NNRTI-based cART (TDF-3TC-EFV)	Yes	No	PTB, VPTB
Chen et al. (30)	58.4% ZDV monotherapy, 2.9% PI-based cART (LPV/r-ZDV-3TC), 33.5% NNRTI-based cART (NVP-ZDV-3TC) 5.2% unspecified cART	No	Yes	PTB, SGA
Chibwesha et al. (31)	66.6% ZDV monotherapy, 33.4% cART (unspecified drug class(es))	Yes	Yes	LBW
Cooper et al. (32)	62.0% ZDV monotherapy, 16.2% dual therapy (96.8% 2 NRTIs, 2.2% NRTI-NNRTI, 0.5% 2 NNRTIs), 21.8% cART (NNRTI-, PI-, or NNRTI-PI based)	Yes	No	PTB, LBW
Cotter et al. (33)	49.3% ZDV monotherapy, 37.3% non-PI-based cART, 13.4% PI-based cART	Yes	No	LBW, VLBW
Dadabhai et al. (34)	NNRTI-based cART (TDF-3TC-EFV)	No	Yes	PTB, LBW, Term LBW, Preterm LBW, SGA, VSGA
De Souza et al. (35)	ZDV monotherapy	Yes	No	PTB
Djeha et al. (36)	85.6% PI-based ART, 14.4% non-PI-based ART (regimen complexities unspecified)	Yes	No	SGA
Duryea et al. (37)	72.2% PI-based cART, 27.8% non-PI-based ART (regimen complexities unspecified)	Yes	No	PTB, SGA
European Collaborative Study (38)	52.4% ZDV monotherapy, 13.4% dual therapy, 34.2% PI/non-PI based cART	Yes	No	LBW
Gagnon et al. (39)	1% monotherapy, 22% non-PI-based ART, 77% PI-based ART (regimen complexities unspecified)	No	Yes	PTB, LBW, SGA
Garcia-Otero et al. (40)	cART (29.8% NNRTI-containing, 66.0% PI-containing, 14.9% INSTI-containing)	No	Yes	PTB, SGA, NND
Gibango et al. (41)	ZDV-containing dual therapy, NNRTI-based cART (proportions unspecified)	Yes	Yes	PTB, LBW, VLBW
Goetghebuer et al. (42)	77.3% PI-based cART, 12.9% NNRTI-based cART, 5.3% NRTI-based cART, 4.5% other regimen	No	Yes	PTB, LBW
Gonzales et al. (43)	ZDV monotherapy, cART (proportions/drug class(es) unspecified)	No	Yes	PTB, LBW, NND
Habib et al. (44)	Unspecified ART	Yes	Yes	PTB, SGA
Haeri et al. (45)	cART (94% NRTI-containing, 20% NNRTI-containing, 74% PI-containing)	No	Yes	PTB, sPTB, Term LBW, SGA
Hernandez et al. (46)	4.2% ZDV monotherapy, 33.3% NNRTI-based cART, 58.3% PI-based cART, 4.2% NRTI-based cART	No	Yes	SGA
Hofer et al. (47)	35.2% ZDV monotherapy, 15.2% dual therapy, 15.9% NNRTI-based cART, 33.7% PI-based cART	Yes	No	PTB
Hu et al. (48)	20.1% ZDV monotherapy/ZDV-3TC dual therapy, 79.9% cART (NNRTI-/PI- based)	Yes	No	PTB, SGA
Joseph et al. (49)	NNRTI-based cART (NVP)	Yes	No	LBW
Jumare et al. (50)	cART (drug class(es) unspecified)	No	Yes	LBW
Kakkar et al. (51)	16.8% ZDV monotherapy, 14.5% NRTI-/NNRTI- containing dual therapy/cART, 68.7% PI-based cART	Yes	No	PTB
Kowalska et al. (52)	43.2% ZDV monotherapy, 22.2% PI-based cART, 34.6% non-PI-based cART	Yes	No	PTB, LBW
Li et al. (10)	61.8% ZDV monotherapy, 35.5% NNRTI-based cART, 0.6% PI-based cART, 2.1% unspecified cART	Yes	No	PTB, LBW, SGA, VSGA
Li et al. (53)	24.2% mono/dual therapy, 75.8% cART (drug class(es) unspecified)	Yes	Yes	PTB, LBW, SGA
Liff et al. (54)	78.0% NNRTI-based cART, 12% INSTI-based cART, 10% other cART	No	Yes	PTB
Lopez et al. (55)	cART (98.7% NRTI-containing, 51.3% NNRTI-containing, 59.7% PI-containing)	No	Yes	PTB, sPTB
Malaba et al. (56)	71.6% NNRTI-based cART, 2.3% PI-based cART, 26.1% other cART	No	Yes	PTB, VPTB, LBW, VLBW, SGA
Malaba et al. (57)	92.5% NNRTI-based cART, 2.8% PI-based cART, 4.7% other cART	No	Yes	PTB, SGA
Mandelbrot et al. (58)	ZDV monotherapy	Yes	No	PTB
Marazzi et al. (59)	NRTI-/NNRTI-based cART (proportions unspecified)	Yes	No	PTB
Marti et al. (60)	15.1% ZDV monotherapy, 13.8% NRTI dual therapy, 7.9% NNRTI-based cART, 61.8% PI-based cART, 1.4% NRTI-based cART	Yes	No	PTB, LBW
Matheson et al. (61)	ZDV monotherapy	Yes	No	PTB
Mehta et al. (62)	98.0% NNRTI-based cART, 0.9% PI-based cART, 1.1% unspecified cART	No	Yes	PTB, LBW, SGA, NND
Moodley et al. (63)	27.5% ZDV monotherapy, 72.5% NNRTI-based cART	Yes	Yes	PTB, LBW, SGA
Moseholm et al. (64)	13.6% NNRTI-based cART, 78.4% PI-based cART, 5.7% NRTI-based cART, 2.3% unspecified cART	No	Yes	PTB
Olagbuji et al. (65)	NNRTI-based cART (ZDV/3TC/NVP)	No	Yes	LBW
Orloff et al. (66)	NRTI(ZDV)-containing ART	Yes	No	PTB
Phiri et al. (67)	20.0% ZDV monotherapy, 15.3% NRTI-NNRTI dual therapy, 21.3% NRTI dual therapy/cART, 43.4% PI-based therapy (unspecified regimen complexity)	Yes	No	PTB, SGA
Ramokolo et al. (68)	38.5% ZDV monotherapy, 61.5% NNRTI-based cART (TDF-3TC/FTC-NVP)	Yes	Yes	PTB, LBW, SGA
Rempis et al. (69)	NNRTI-based cART (TDF-3TC-EFV)	No	Yes	SGA
Rudin et al. (70)	26.4% ZDV mono/dual therapy, 61.8% PI-based cART, 11.8% non-PI-based cART	Yes	No	PTB, VPTB
Santosa et al. (71)	1.6% ZDV monotherapy, 96.0% cART, 2.4% unspecified regimen	No	Yes	PTB, VPTB, LBW, VLBW, SGA, VSGA, Stillbirth, NND
Saums et al. (72)	10.9% NNRTI-based cART, 54.7% PI-based cART, 34.3% INSTI-based cART	No	Yes	PTB
Schulte et al. (73)	42.1% monotherapy, 16.7% dual therapy, 12.6% PI-based cART, 28.6% non-PI-based cART	Yes	No	PTB, LBW
Sebitloane and Moodley (74)	36.6% ZDV monotherapy, 63.4% NNRTI-based cART	No	Yes	PTB
Short et al. (75)	20.1% ZDV monotherapy, 2.2% NRTI dual therapy, 42.4% NNRTI-based cART, 29.8% PI-based cART, 1.5% NRTI-based cART, 4.0% unspecified cART	Yes	No	PTB
Silverman (76)	PI-based cART (ZDV-3TC-LPV/r)	Yes	No	LBW
Simonds et al. (77)	ZDV monotherapy	Yes	No	PTB, LBW
Snijdewind et al. (78)	31.5% NNRTI-based cART, 66.7% PI-based cART, 1.8% other cART	No	Yes	PTB, VPTB, LBW, VLBW, SGA
Tiam et al. (79)	96.5% NNRTI-based cART, 2.3% other cART, 2.2% no ART	No	Yes	PTB, LBW, VLBW
Townsend et al. (ECS) (80)	27.8% monotherapy, 11.8% NRTI dual therapy, 36.2% PI-based cART, 24.2% non-PI-based cART	Yes	No	PTB
Townsend et al. (NSHPC) (80)	16.3% monotherapy, 3.2% dual therapy, 42.0% PI-based cART, 38.5% non-PI-based cART	Yes	No	PTB
Tuomala et al. (81)	74.8% ZDV monotherapy, 6.5% PI-based dual/cART, 18.7% non-PI-based dual/cART	Yes	No	PTB, VPTB, LBW, VLBW
Van der Merwe et al. (82)	42.8% NNRTI-based cART, 44.5% PI-based cART, 12.7% unspecified cART	Yes	No	PTB, LBW, VLBW
Von Linstow et al. (83)	12.1% ZDV monotherapy/dual therapy, 87.9% NNRTI-/PI-based cART	Yes	No	LBW
Watts et al. (84)	7.6% mono/dual therapy, 8.8% NNRTI-based cART, 72.9% PI-based cART, 10.7% NRTI-based cART	Yes	No	PTB, sPTB
Wedderburn et al. (85)	12.0% ZDV monotherapy, 81.0% NNRTI-based cART, 7.0% PI-based cART	No	Yes	PTB, LBW
Wilkinson et al. (86)	61.3% ZDV monotherapy, 34.1% NNRTI-based cART (ZDV-3TC-EFV), 4.5% no ART	No	Yes	PTB, LBW
Yu et al. (87)	NNRTI-based cART	Yes	No	PTB, LBW
Zash et al. (88)	72.7% NNRTI-based cART (TDF-FTC-EFV), 27.3% INSTI-based cART (TDF-FTC-DTG)	No	Yes	PTB, VPTB, SGA, VSGA, NND
Ziske et al. (89)	ZDV monotherapy	Yes	No	PTB

3TC, lamivudine; ART, antiretroviral therapy; ATV/r, ritonavir-boosted atazanavir; cART, combination antiretroviral therapy (≥ 3 antiretroviral drugs); DRV/r, ritonavir-boosted darunavir; ECS, european collaborative study; EFV, efavirenz; FTC, emtricitabine; INSTI, integrase inhibitor; LBW, low birthweight; LPV/r, ritonavir-boosted lopinavir; NND, neonatal death; NNRTI, non-nucleoside transcriptase inhibitor; NRTI, nucleoside reverse transcriptase inhibitor; NSHPC, national study of HIV in pregnancy and childhood; NVP, nevirapine; PI, protease inhibitor; PTB, preterm birth; SGA, small for gestational age; sPTB, spontaneous preterm birth; TDF, tenofovir disoproxil fumarate; VLBW, very low birthweight; VPTB, very preterm birth; VSGA, very small for gestational age; WLHIV, women living with HIV; ZDV, zidovudine.

**FIGURE 2:**
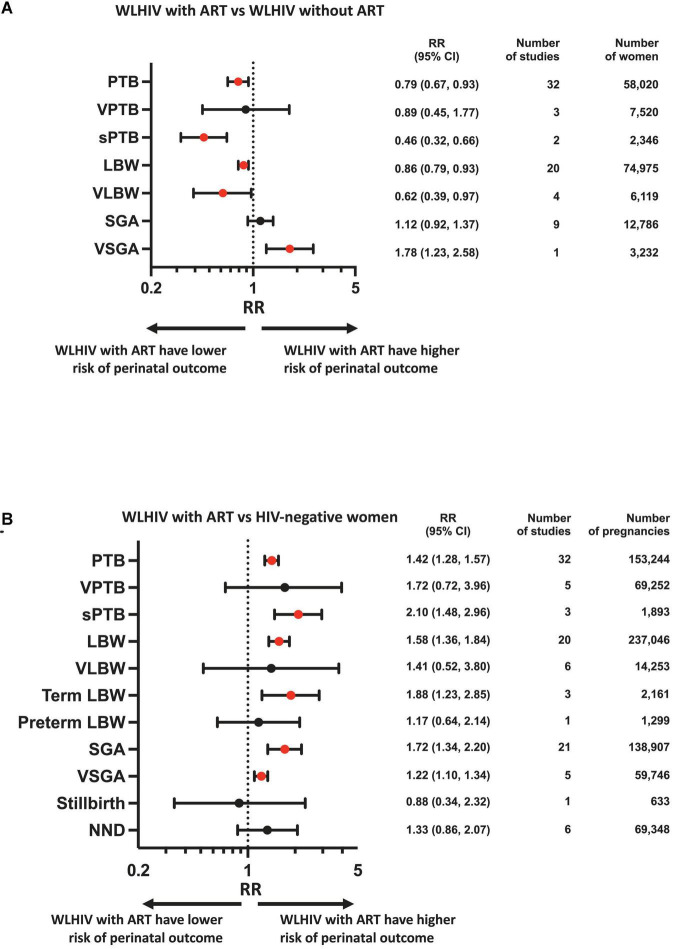
Perinatal outcomes of women living with HIV receiving ART compared to women living with HIV without ART and HIV-negative women. Random-effects meta-analysis results for perinatal outcomes associated with women living with HIV receiving ART compared to women living with HIV without ART **(A)** and HIV-negative women **(B)**. Statistically significant effects are presented with red dots and non-significant effects with black dots. ART, antiretroviral therapy; HIV, human immunodeficiency virus; LBW, low birthweight; NND, neonatal death; PTB, preterm birth; RR, relative risk; SGA, small for gestational age; sPTB, spontaneous preterm birth; VLBW, very low birthweight; VPTB, very preterm birth; VSGA, very small for gestational age; WLHIV, women living with HIV; 95% CI, 95% confidence interval.

**FIGURE 3:**
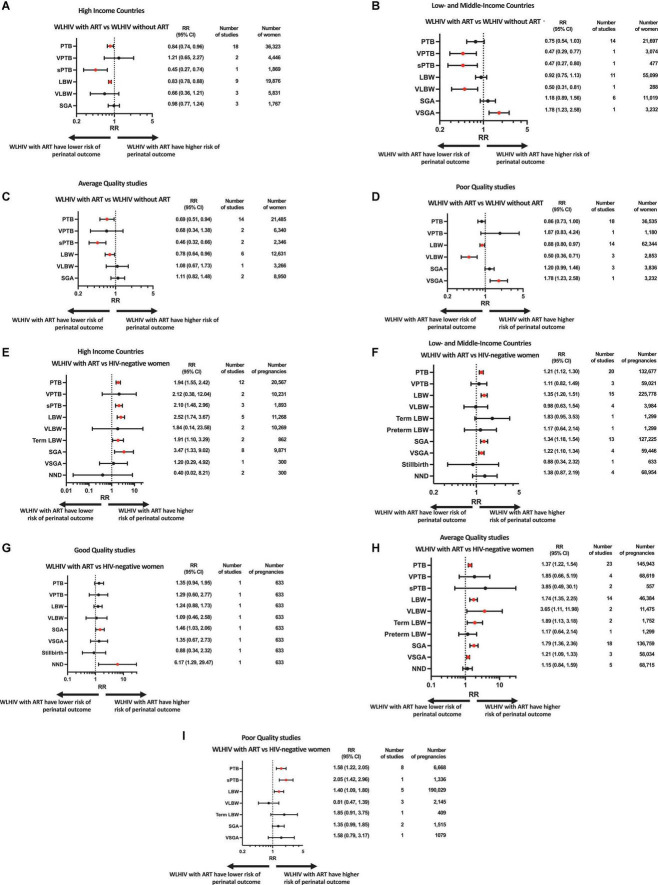
Subgroup and sensitivity analyses based on country income status and study quality. Random-effects meta-analysis results for perinatal outcomes associated with women living with HIV receiving ART compared to women living with HIV without ART **(A–D)** and HIV-negative women **(E–I)**. Subgroups consisted of studies done in high income countries **(A,E)** or low- and middle-income countries **(B,F)**. Sensitivity analysis was done for studies of good **(G)**, average **(C,H)** or poor **(D,I)** quality. Statistically significant effects are presented with red dots and non-significant effects with black dots. ART, antiretroviral therapy; HIV, human immunodeficiency virus; LBW, low birthweight; NND, neonatal death; PTB, preterm birth; RR, relative risk; SGA, small for gestational age; sPTB, spontaneous preterm birth; VLBW, very low birthweight; VPTB, very preterm birth; VSGA, very small for gestational age; WLHIV, women living with HIV; 95% CI, 95% confidence interval.

Random-effects meta-analyses were conducted to compare perinatal outcomes in WLHIV receiving ART with WLHIV without ART and HIV-negative women. The summary effect estimates are presented in Figure 2 and the forest plots in Supplementary Appendix 3. Subgroup analyses were carried out according to country income status (Figures 3A, B, E, F), and study quality (Figures 3C, D, G–I).

### WLHIV receiving ART vs. WLHIV without ART

41 studies, including 288,296 women, reported on seven perinatal outcomes in WLHIV receiving ART compared to WLHIV without ART.

In the analysis of 58,020 women from 32 studies, WLHIV receiving ART were associated with a significantly decreased risk of PTB compared to WLHIV without ART (RR 0.79, 95% CI 0.67–0.93) (Figure 2A). Heterogeneity between studies was high (*I*^2^ 90.1%, Supplementary Appendix 3.1), but there was no evidence of publication bias (Peters’ test, *p* = 0.395). The significance of this association was retained in subgroup analyses of studies conducted in HICs (0.84, 0.74–0.96) (Figure 3A) and in average quality studies (0.69, 0.51–0.94) (Figure 3C), but not in studies from LMICs or poor quality studies (Figures 3B, D). One study adjusted for covariates, which did not result in a change in the significance of the effect estimate (Supplementary Appendix 4.3).

WLHIV receiving ART were not associated with VPTB compared to WLHIV without ART (Figure 2A). However, in the one study conducted in a LMIC, a significantly decreased risk of VPTB was observed for WLHIV receiving ART (0.47, 0.29–0.77) (Figure 3B), which was not seen in studies from HICs (Figure 3A).

In the analysis of 2,346 women from two average quality studies, a significant association between WLHIV receiving ART and decreased risk of sPTB was observed, compared to WLHIV without ART (0.46, 0.32–0.66) (Figure 2A). There was no heterogeneity (*I*^2^ 0.0%, Supplementary Appendix 3.1). The significance of the association was retained in subgroup analyses of studies conducted in HICs (0.45, 0.27–0.74) and LMICs (0.47, 0.27–0.80) (Figures 3A, B).

In the analysis of 74,975 women from 20 studies, WLHIV receiving ART were associated with a significantly decreased risk of LBW compared to WLHIV without ART (0.86, 0.79–0.93) (Figure 2A). A moderate level of heterogeneity was observed between studies (*I*^2^ 56.1%, Supplementary Appendix 3.1), and there was no evidence of publication bias (Peters’ test, *p* = 0.109). The significance of the association was retained in subgroup analyses of studies conducted in HICs (0.83, 0.78–0.88) (Figure 3A), but not LMICs (Figure 3B), and in average (0.78, 0.64–0.96) and poor quality studies (0.88, 0.80–0.97) (Figures 3C, D). One study adjusted for covariates, which did not result in a change in the significance of the effect estimate (Supplementary Appendix 4.3).

In the analysis of 6,119 women from four studies, WLHIV receiving ART were associated with a significantly decreased risk of VLBW, compared to WLHIV without ART (0.62, 0.39–0.97) (Figure 2A). A moderate level of heterogeneity was observed (*I*^2^ 61.9%) (Supplementary Appendix 3.1). The significance of the association was retained in subgroup analyses of studies conducted in LMICs (0.50, 0.31–0.81) (Figure 3B) and in poor quality studies (0.50, 0.36–0.71) (Figure 3D), but not in studies from HICs or average quality studies (Figures 3A, C).

In the analysis of 12,786 women from nine studies, WLHIV receiving ART were not associated with SGA compared to WLHIV without ART (Figure 2A). There was a moderate level of heterogeneity (*I*^2^ 49.9%) (Supplementary Appendix 3.1) and no significant associations were seen in the subgroup analyses (Figure 3).

In the analysis of 3,232 women from one poor quality study conducted in a LMIC, WLHIV receiving ART were associated with a significantly increased risk of VSGA compared to WLHIV without ART (1.78, 1.23–2.58) (Figures 2A, 3B, D).

No data was found for WLHIV receiving ART compared to WLHIV without ART for term and preterm LBW, stillbirth, and NND.

### WLHIV receiving ART vs. HIV-negative women

38 studies, including 362,978 women, reported on 11 perinatal outcomes of WLHIV receiving ART compared to HIV-negative women.

In the analysis of 153,244 women from 32 studies, WLHIV receiving ART were associated with a significantly increased risk of PTB, compared to HIV-negative women (1.42, 1.28–1.57) (Figure 2B). Heterogeneity was high (*I*^2^ 86.5%, Supplementary Appendix 3.2), but there was no evidence of publication bias (Peters’ test, *p* = 0.371). The significant association was retained in subgroup analyses by country income status, with a higher relative risk estimate in HICs (1.94, 1.55–2.42) (Figure 3E) than LMICs (1.21, 1.12–1.30) (Figure 3F). The association was significant in average and poor quality studies (Figures 3H, I), but not the single good quality study (Figure 3G). Of the 11 studies which adjusted for covariates, only one resulted in a change in the significance of the effect estimate (Supplementary Appendix 4.1).

WLHIV receiving ART were not associated with VPTB, compared to HIV-negative women (Figure 2B). There was a high level of heterogeneity (*I*^2^ 92.0%) (Supplementary Appendix 3.2).

In the analysis of 1,893 women from three studies conducted in HICs, WLHIV receiving ART were associated with a significantly increased risk of sPTB (2.10, 1.48–2.96), compared to HIV-negative women (Figures 2B, 3E). There was no heterogeneity (*I*^2^ 12.5%, Supplementary Appendix 3.2). The significance of this association was retained in subgroup analyses of the single poor quality study (2.05, 1.42–2.96) (Figure 3I), but not in the average quality studies (Figure 3H). One study adjusted for covariates, which did not result in a change in the significance of the effect estimate (Supplementary Appendix 4.1).

In the analysis of 237,046 women from 20 studies, WLHIV receiving ART were associated with a significantly increased risk of LBW compared to HIV-negative women (1.58, 1.36–1.84) (Figure 2B). Heterogeneity was high (*I*^2^ 90.1%, Supplementary Appendix 3.2), but there was no evidence of publication bias (Peters’ test, *p* = 0.407). The significant association was retained in subgroup analyses by country income status, with a higher relative risk in HICs (2.52, 1.74–3.67) (Figure 3E) than LMICs (1.35, 1.20–1.51) (Figure 3F). The association was significant in average and poor quality studies (Figures 3H, I), but not the single good quality study (Figure 3G). Of the seven studies which adjusted for covariates, this resulted in a change in the significance of the effect estimate in two studies (Supplementary Appendix 4.1).

WLHIV receiving ART were not associated with VLBW, compared to HIV-negative women (Figure 2B). There was a high level of heterogeneity (*I*^2^ 91.1%, Supplementary Appendix 3.2). The association was significant for two average quality studies (3.65, 1.11–11.98) (Figure 3H).

In the analysis of 2,161 women from three studies, WLHIV receiving ART were associated with a significantly increased risk of term LBW compared to HIV-negative women (1.88, 1.23–2.85) (Figure 2B). There was no heterogeneity (*I*^2^ 0.0%, Supplementary Appendix 3.2). The significance of the association was retained in subgroup analyses of average quality studies (1.89, 1.13–3.18) (Figure 3H), but not in the single poor quality study (Figure 3I). There was no significant association in subgroup analyses by country income status (Figures 3E, F).

WLHIV receiving ART were not associated with preterm LBW, compared to HIV-negative women (Figure 2B).

In the analysis of 138,907 women from 21 studies, WLHIV receiving ART were associated with a significantly increased risk of SGA compared to HIV-negative women (1.72, 1.34–2.20) (Figure 2B). Heterogeneity was high (*I*^2^ 97.1%, Supplementary Appendix 3.2), but there was no evidence of publication bias (Peters’ test, *p* = 0.692). The significant association was retained in subgroup analyses by country income status, with a higher RR in HICs (3.47, 1.33–9.02) (Figure 3E) than LMICs (1.34, 1.18–1.54) (Figure 3F), and in good and average quality studies (Figures 3G, H), but not poor quality studies (Figure 3I). Of the ten studies which adjusted for covariates, this resulted in a change in the significance of the effect estimate in three studies (Supplementary Appendix 4.2).

In the analysis of 59,746 women from five studies, WLHIV receiving ART were associated with a significantly increased risk of VSGA, compared to HIV-negative women (1.22, 1.10–1.34) (Figure 2B). There was no heterogeneity (*I*^2^ 0.0%, Supplementary Appendix 3.2). The significant association was retained in subgroup analyses of studies conducted in LMICs (1.22, 1.10–1.34) (Figure 3F), but not in the single study from a HIC (Figure 3E). The significant association was retained in subgroup analyses of average quality studies (Figure 3H), but not poor or high quality studies (Figures 3G, I). Two studies adjusted for covariates, which did not result in a change in the significance of the effect estimate (Supplementary Appendix 4.2).

WLHIV receiving ART were not associated with stillbirth, compared to HIV-negative women (Figure 2B).

WLHIV receiving ART were not associated with NND, compared to HIV-negative women (Figure 2B). However, in the one good quality study a significantly increased risk of NND was observed for WLHIV receiving ART (6.17, 1.29, 29.47) (Figure 3G).

## Discussion

This meta-analysis shows that WLHIV receiving ART are associated with a significantly decreased risk of PTB, sPTB, LBW, and VLBW compared to WLHIV without ART. However, WLHIV receiving ART are associated with a significantly increased risk of PTB, sPTB, LBW, term LBW, SGA, and VSGA compared to HIV-negative women. Therefore, ART reduces the risk of adverse perinatal outcomes in pregnant WLHIV, but perinatal outcomes remain higher than in HIV-negative women.

As the proportion of pregnant WLHIV that receive ART during pregnancy continues to increase, it is an important finding that ART not only improves maternal health and reduces perinatal HIV transmission, but also improves perinatal outcomes in WLHIV. The decreased risk of PTB and LBW in WLHIV receiving ART was observed in HICs, but not in LMICs. This suggests that the benefits of ART in pregnancy may be diminished in LMIC settings, which may be attributable to initiation of ART late in pregnancy, which remains common in LMICs (14). As more WLHIV in LMICs initiate life-long ART from before pregnancy, this may further improve the perinatal outcomes of WLHIV in LMICs.

Our findings agree with a smaller meta-analysis by Shinar et al. which reported that WLHIV receiving ART are associated with a higher risk of PTB, LBW, and SGA compared to HIV-negative women (90). Our analysis includes 73 studies and examines 11 outcomes in contrast to the 27 studies and 4 outcomes examined in Shinar et al. Furthermore, our analysis examines whether ART improves perinatal outcomes in WLHIV. Our finding that WLHIV receiving ART are at increased risk of adverse perinatal outcomes compared to HIV-negative women also aligns with a previous meta-analyses reporting increased risks of adverse perinatal outcomes in WLHIV without ART (3). Importantly, the effect estimates for WLHIV receiving ART compared to HIV-negative women in the current analysis were smaller than those previously reported for WLHIV without ART compared to HIV-negative women: the relative risk of PTB for WLHIV on ART was 1.42 (1.28–1.57) compared to a relative risk of 1.63 (1.37–1.93) for WLHIV without ART; relative risk of LBW of 1.58 (1.36–1.84) for WLHIV on ART compared to 1.75 (1.52–2.02) for WLHIV without ART, and relative risk of stillbirth of 0.88 (0.34–2.32) for WLHIV on ART compared to 1.67 (1.05–2.66) for WLHIV without ART (3). This is consistent with our finding that ART improves perinatal outcomes in pregnant WLHIV women. It is noteworthy, however, that the reductions in relative risk estimates are modest and that the risks of adverse perinatal outcomes remain high in WLHIV receiving ART compared to HIV-negative women.

The increased risk for WLHIV receiving ART, compared to HIV-negative women, was found in both HICs and LMICs, and the relative risk estimates of PTB, LBW, and SGA were higher in HICs than in LMICs. This is despite the improvements of perinatal outcomes with ART in WLHIV, compared to WLHIV without ART, which were observed in HICs, but not LMICs. This may in part be due to the levels of adverse perinatal outcomes in HIV-negative women, which are low in HICs, but very high in some LMICs (71, 88).

This study has several strengths. It is the largest study to date reporting on a comprehensive range of adverse perinatal outcomes associated with WLHIV receiving ART, including 424,277 women from 73 studies. Importantly, the significant findings for PTB, LBW, and SGA were each powered by ≥20 studies with > 58,000 participants, thereby providing strong evidence for the associations found. The study was conducted according to Cochrane guidelines, with exposures and outcomes clearly defined at the outset to reduce misclassification bias and ensure consistency across studies.

This study has a number of limitations. All studies included are observational and are therefore associated with an increased risk of bias, which was extensively assessed for each study. Indeed, in studies that corrected for covariates using regression analysis, only 6 comparisons (15%) resulted in a change in significance of the effect estimate. Additionally, cohort studies may be more representative of events in the real world, compared to trials in which ART is initiated during pregnancy, often in the second or third trimester (i.e. 12, 91, 92). There were few studies (< 5) reporting on comparisons for several perinatal outcomes, including VPTB, sPTB, VLBW, term LBW, preterm LBW, VSGA, and stillbirth, which renders the results for these outcomes less reliable. 23 studies did not describe a method to estimate gestational age, and only six used first trimester ultrasound, which is the most accurate method to determine gestational age (93). Lack of accurate gestational age estimation may lead to misclassification bias for outcomes that rely on gestational age, such as PTB and SGA. Consequently, only one study was classified as “good” quality.

We included studies in which WLHIV receiving ART were exposed to any ART regimen in an effort to capture the overall effect of ART on perinatal outcomes since ART use in pregnancy was introduced. The evidence of the association of different ART regimens with adverse perinatal outcomes is conflicting (9, 13, 94). Some studies have shown an increased risk of PTB with antenatal initiation of cART compared to ZDV monotherapy (30), but this was not seen in other studies (10, 95). A recent meta-analysis suggested that ZDV monotherapy decreases the risk of PTB and LBW compared to ART-naïve WLHIV, while cART does not (96). Similarly, protease inhibitor containing cART was associated with an increased risk of PTB in a number of studies (11), but not in others (97). Preconception initiation of ART may be associated with increased risk of adverse outcomes compared to ART initiation during pregnancy, although this is disputed by others (13, 14). Differential ART regimens, as well as differences in the populations, settings, and methods to estimate gestational age between included studies, may have contributed to the heterogeneity observed in our analyses.

There is a need to determine the optimal ART regimen for use in pregnancy. Current WHO guidance recommends dolutegravir (DTG)-containing regimens as preferred first-line ART, including for women of childbearing potential and pregnant women (98). A retrospective cohort study from Botswana showed that perinatal outcomes were comparable between WLHIV receiving DTG-based and efavirenz (EFV)-based ART (88). Recent randomized controlled trials of ART regimens initiated during pregnancy showed that DTG-containing regimens had superior virological efficacy compared to EFV-based ART (91, 92), and that a regimen containing DTG, emtricitabine and tenofovir alafenamide fumarate had the lowest rate of adverse pregnancy outcomes (92).

The biological mechanisms contributing to the associations between HIV status, antenatal ART and adverse perinatal outcomes remain unclear. The pathogenesis underlying adverse perinatal outcomes is multifaceted, and the cause is often unknown (99). Our data indicate that perinatal outcomes in WLHIV receiving ART remain higher than in HIV-negative women, suggesting that adverse perinatal outcomes may be related to physiological changes resulting from HIV infection which are not reversed by ART. HIV-infection is associated with depletion of CD4+ T cells and chronic immune activation (100), which may interfere with the immunological processes of pregnancy. However, despite the success of ART in suppressing viral load, some people living with HIV never achieve full CD4+ T cell recovery (101). ART may promote a shift toward pro-inflammatory Th1 activity, counteracting the Th1 to Th2 shift required to support pregnancy (102). A number of innate immune cells, including innate lymphoid cells and mucosal associated invariant T cells, are rapidly depleted early after HIV infection, which is irreversible by institution of ART and may be associated with an increased risk of PTB (103, 104). It was reported that WLHIV receiving protease inhibitors have lower plasma progesterone levels, which was proposed as a potential mediator of adverse outcomes in WLHIV. Interestingly, a recent RCT of progesterone supplementation in pregnant WLHIV on ART (mostly NNRTI-ART, only 3% PI-ART), showed that administration of 17-alpha-hydroxyprogesterone had no effect on the primary outcomes of PTB or stillbirth, but was instead associated with a reduction in the risk of VSGA (105).

We have shown that ART reduces the risk of adverse perinatal outcomes in pregnant WLHIV, thereby supporting the WHO policy of initiation of ART at diagnosis for all people living with HIV, including pregnant women (98). However, the risk of adverse perinatal outcomes remains high in the increasing number of WLHIV who receive ART, compared to HIV-negative women, which continues to contribute to the global burden of adverse perinatal outcomes and limit progress toward achieving Sustainable Development Goal 3 (15). Further studies are urgently needed to determine the optimal ART regimen(s) in pregnancy to minimize adverse perinatal outcomes in WLHIV, elucidate the mechanism underlying adverse perinatal outcomes in WLHIV, and develop preventative and therapeutic interventions to improve perinatal outcomes in WLHIV.

## Data availability statement

The original contributions presented in this study are included in the article/Supplementary material, further inquiries can be directed to the corresponding author.

## Author contributions

CP, HS, MK, and ZB screened the literature search results for relevant manuscripts and assessed their eligibility, extracted data, and conducted methodological quality assessments. CP conducted the meta-analyses, subgroup and sensitivity analyses, interpreted the data, and wrote the first draft of the manuscript. SK designed and conducted the literature search. JH conceived, designed, coordinated the study, developed the systematic review protocol, assisted with the literature search, assessment of eligibility of manuscripts, data extraction, and methodological quality assessment, designed the meta-analysis plan, interpreted the data, wrote the manuscript, had full access to all the data in the study, and had final responsibility for the decision to submit the manuscript for publication. All authors read and approved the final version of the manuscript.
