# Supplementary material for: Adverse perinatal outcomes associated with antiretroviral therapy in women living with HIV: A systematic review and meta-analysis
Source: Front Med (Lausanne). 2023 Feb 3;9:924593. doi: 10.3389/fmed.2022.924593 (PMC9935588; doi:10.3389/fmed.2022.924593)
Supplement: Supplementary file 1 [file Data_Sheet_1.pdf]

Adverse perinatal outcomes associated with antiretroviral therapy in women living with HIV: a systematic review and meta-analysis

Appendices

Appendix 1: Literature search strategies **1**

Appendix 2: Quality assessment of studies **14**

Appendix 2.1: Adapted Newcastle-Ottawa quality assessment tool 14

Appendix 2.2: Classification of studies according to quality assessment 16

Appendix 2.3: Quality assessment of studies included in the systematic review and meta-analysis 17

Appendix 2.4: Confounding factors adjusted for in included studies 25

Appendix 3: Random-effects meta-analyses of risk of perinatal outcomes associated with pregnancies in women living with HIV receiving ART **30**

Appendix 3.1: WLHIV receiving ART vs WLHIV without ART 30

Appendix 3.2: WLHIV receiving ART vs HIV-negative women 34

Appendix 4: Adjusting for confounders in individual studies **41**

Appendix 4.1: Sensitivity analysis: adjusting for confounders in individual studies comparing WLHIV receiving ART to HIV-negative women for preterm birth (PTB), very preterm birth (VPTB), spontaneous preterm birth (sPTB), low birthweight (LBW), and very low birthweight (LBW) 41

Appendix 4.2: Sensitivity analysis: adjusting for confounders in individual studies comparing WLHIV receiving ART to HIV-negative women for small for gestational age (SGA), very small for gestational age (VSGA), stillbirth, and neonatal death (NND) 44

Appendix 4.3: Sensitivity analysis: adjusting for confounders in individual studies comparing WLHIV receiving ART to WLHIVwithout ART for preterm birth (PTB), very preterm birth (VPTB), spontaneous preterm birth (sPTB), low birthweight (LBW), and very low birthweight (LBW) 47

Appendix 1
Literature search strategies

Search strategy for “pregnancy outcome AND HIV OR antiretroviral therapy”

Database and platform: Pubmed (via https://www.ncbi.nlm.nih.gov/pubmed/)

Latest search date: 20 April 2020.

1. Pregnancy Outcome [Mesh] OR Pregnancy Complications, Infectious [Mesh] OR "pregnancy outcome*" [tiab] OR "pregnancy complication*" [tiab] OR pregnancy consequence* [tiab] OR "pregnancy characteristic" [tiab] OR "pregnancy characteristics" [tiab] OR pregnancy event* [tiab] OR pregnancy result* [tiab] OR pregnancy problem* [tiab] OR pregnancy morbidit* [tiab] OR pregnancy sequelae [tiab]

2. gestational outcome* [tiab] OR gestational complication* [tiab] OR gestational consequence* [tiab] OR gestational characteristic* [tiab] OR gestational event* [tiab] OR gestational result* [tiab] OR gestational problem* [tiab] OR gestational morbidit* [tiab] OR gestational sequelae [tiab]

3. "fetal outcome" [tiab] OR "fetal outcomes" [tiab] OR "fetal complication" [tiab] OR "fetal complications" [tiab] OR fetal consequence* [tiab] OR fetal characteristic* [tiab] OR fetal event* [tiab] OR fetal result* [tiab] OR fetal problem* [tiab] OR fetal morbidit* [tiab] OR fetal sequelae [tiab]

4. foetal outcome* [tiab] OR foetal complication* [tiab] OR foetal consequence* [tiab] OR foetal characteristic* [tiab] OR foetal event* [tiab] OR foetal result* [tiab] OR foetal problem* [tiab] OR foetal morbidit* [tiab] OR foetal sequelae [tiab]

5. "obstetric outcome" [tiab] OR "obstetric outcomes" [tiab] OR "obstetric complication" [tiab] OR "obstetric complications" [tiab] OR obstetric consequence* [tiab] OR obstetric characteristic* [tiab] OR obstetric event* [tiab] OR obstetric result* [tiab] OR "obstetric problem" [tiab] OR "obstetric problems" [tiab] OR obstetric morbidit* [tiab] OR obstetric sequelae [tiab]

6. obstetrical outcome* [tiab] OR "obstetrical complication" [tiab] OR "obstetrical complications" [tiab] OR obstetrical consequence* [tiab] OR obstetrical characteristic* [tiab] OR obstetrical event* [tiab] OR obstetrical result* [tiab] OR "obstetrical problem" [tiab] OR "obstetrical problems" [tiab] OR obstetrical morbidit* [tiab] OR obstetrical sequelae [tiab]

7. "labor outcome" [tiab] OR "labor outcomes" [tiab] OR "labor complication" [tiab] OR "labor complications" [tiab] OR "labor consequence" [tiab] OR "labor consequences" [tiab] OR labor characteristic* [tiab] OR labor event* [tiab] OR labor result* [tiab] OR labor problem* [tiab] OR labor morbidit* [tiab] OR labor sequelae [tiab]

8. labour outcome* [tiab] OR labour complication* [tiab] OR "labour consequence" [tiab] OR "labour consequences" [tiab] OR labour characteristic* [tiab] OR labour event* [tiab] OR labour result* [tiab] OR labour problem* [tiab] OR labour morbidit* [tiab] OR labour sequelae [tiab]

9. "birth outcome" [tiab] OR "birth outcomes" [tiab] OR birth complication* [tiab] OR birth consequence* [tiab] OR birth characteristic* [tiab] OR birth event* [tiab] OR birth result* [tiab] OR birth problem* [tiab] OR birth morbidit* [tiab] OR birth sequelae [tiab]

10. delivery outcome* [tiab] OR delivery complication* [tiab] OR delivery consequence* [tiab] OR delivery characteristic* [tiab] OR delivery event* [tiab] OR delivery result* [tiab] OR delivery problem* [tiab] OR delivery morbidit* [tiab] OR delivery sequelae [tiab]

11. neonate outcome* [tiab] OR neonate complication* [tiab] OR neonate consequence* [tiab] OR neonate characteristic* [tiab] OR neonate event* [tiab] OR neonate result* [tiab] OR neonate problem* [tiab] OR neonate morbidit* [tiab] OR neonate sequelae [tiab]

12. newborn outcome* [tiab] OR newborn complication* [tiab] OR newborn consequence* [tiab] OR newborn characteristic* [tiab] OR newborn event* [tiab] OR newborn result* [tiab] OR newborn problem* [tiab] OR newborn morbidit* [tiab] OR newborn sequelae [tiab]

13. new-born outcome* [tiab] OR new-born complication* [tiab] OR new-born consequence* [tiab] OR new-born characteristic* [tiab] OR new-born event* [tiab] OR new-born result* [tiab] OR new-born problem* [tiab] OR new-born morbidit* [tiab] OR new-born sequelae [tiab]

14. new born outcome* [tiab] OR new born complication* [tiab] OR new born consequence* [tiab] OR new born characteristic* [tiab] OR new born event* [tiab] OR new born result* [tiab] OR new born problem* [tiab] OR new born morbidit* [tiab] OR new born sequelae [tiab]

15. infant outcome* [tiab] OR infant complication* [tiab] OR infant consequence* [tiab] OR infant characteristic* [tiab] OR infant event* [tiab] OR infant result* [tiab] OR infant problem* [tiab] OR infant morbidit* [tiab] OR infant sequelae [tiab]

16. reproductive outcome* [tiab] OR reproductive complication* [tiab] OR reproductive consequence* [tiab] OR reproductive characteristic* [tiab] OR reproductive event* [tiab] OR reproductive result* [tiab] OR reproductive problem* [tiab] OR reproductive morbidit* [tiab] OR reproductive sequelae [tiab]

17. prelabour outcome* [tiab] OR prelabour complication* [tiab] OR prelabour consequence* [tiab] OR prelabour characteristic* [tiab] OR prelabour event* [tiab] OR prelabour result* [tiab] OR prelabour problem* [tiab] OR prelabour morbidit* [tiab] OR prelabour sequelae [tiab]

18. prelabor outcome* [tiab] OR prelabor complication* [tiab] OR prelabor consequence* [tiab] OR prelabor characteristic* [tiab] OR prelabor event* [tiab] OR prelabor result* [tiab] OR prelabor problem* [tiab] OR prelabor morbidit* [tiab] OR prelabor sequelae [tiab]

19. pre-labour outcome* [tiab] OR pre-labour complication* [tiab] OR pre-labour consequence* [tiab] OR pre-labour characteristic* [tiab] OR pre-labour event* [tiab] OR pre-labour result* [tiab] OR pre-labour problem* [tiab] OR pre-labour morbidit* [tiab] OR pre-labour sequelae [tiab]

20. pre-labor outcome* [tiab] OR pre-labor complication* [tiab] OR pre-labor consequence* [tiab] OR pre-labor characteristic* [tiab] OR pre-labor event* [tiab] OR pre-labor result* [tiab] OR pre-labor problem* [tiab] OR pre-labor morbidit* [tiab] OR pre-labor sequelae [tiab]

21. intrauterine outcome* [tiab] OR intrauterine complication* [tiab] OR intrauterine consequence* [tiab] OR intrauterine characteristic* [tiab] OR intrauterine event* [tiab] OR intrauterine result* [tiab] OR intrauterine problem* [tiab] OR intrauterine morbidit* [tiab] OR intrauterine sequelae [tiab]

22. intra-uterine outcome* [tiab] OR intra-uterine complication* [tiab] OR intra-uterine consequence* [tiab] OR intra-uterine characteristic* [tiab] OR intra-uterine event* [tiab] OR intra-uterine result* [tiab] OR intra-uterine problem* [tiab] OR intra-uterine morbidit* [tiab] OR intra-uterine sequelae [tiab]

23. antenatal outcome* [tiab] OR antenatal complication* [tiab] OR antenatal consequence* [tiab] OR antenatal characteristic* [tiab] OR antenatal event* [tiab] OR antenatal result* [tiab] OR antenatal

problem* [tiab] OR antenatal morbidit* [tiab] OR antenatal sequelae [tiab]

24. ante-natal outcome* [tiab] OR ante-natal complication* [tiab] OR ante-natal consequence* [tiab] OR ante-natal characteristic* [tiab] OR ante-natal event* [tiab] OR ante-natal result* [tiab] OR ante-natal problem* [tiab] OR ante-natal morbidit* [tiab] OR ante-natal sequelae [tiab]

25. prenatal outcome* [tiab] OR prenatal complication* [tiab] OR prenatal consequence* [tiab] OR prenatal characteristic* [tiab] OR prenatal event* [tiab] OR prenatal result* [tiab] OR prenatal problem* [tiab] OR prenatal morbidit* [tiab] OR prenatal sequelae [tiab]

26. pre-natal outcome* [tiab] OR pre-natal complication* [tiab] OR pre-natal consequence* [tiab] OR pre-natal characteristic* [tiab] OR pre-natal event* [tiab] OR pre-natal result* [tiab] OR pre-natal problem* [tiab] OR pre-natal morbidit* [tiab] OR pre-natal sequelae [tiab]

27. perinatal outcome* [tiab] OR perinatal complication* [tiab] OR perinatal consequence* [tiab] OR perinatal characteristic* [tiab] OR perinatal event* [tiab] OR perinatal result* [tiab] OR perinatal problem* [tiab] OR perinatal morbidit* [tiab] OR perinatal sequelae [tiab]

28. peri-natal outcome* [tiab] OR peri-natal complication* [tiab] OR peri-natal consequence* [tiab] OR peri-natal characteristic* [tiab] OR peri-natal event* [tiab] OR peri-natal result* [tiab] OR peri-natal problem* [tiab] OR peri-natal morbidit* [tiab] OR peri-natal sequelae [tiab]

29. neonatal outcome* [tiab] OR neonatal complication* [tiab] OR neonatal consequence* [tiab] OR neonatal characteristic* [tiab] OR neonatal event* [tiab] OR neonatal result* [tiab] OR neonatal problem* [tiab] OR neonatal morbidit* [tiab] OR neonatal sequelae [tiab]

30. neo-natal outcome* [tiab] OR neo-natal complication* [tiab] OR neo-natal consequence* [tiab] OR neo-natal characteristic* [tiab] OR neo-natal event* [tiab] OR neo-natal result* [tiab] OR neo-natal problem* [tiab] OR neo-natal morbidit* [tiab] OR neo-natal sequelae [tiab]

31. postnatal outcome* [tiab] OR postnatal complication* [tiab] OR postnatal consequence* [tiab] OR postnatal characteristic* [tiab] OR postnatal event* [tiab] OR postnatal result* [tiab] OR postnatal problem* [tiab] OR postnatal morbidit* [tiab] OR postnatal sequelae [tiab]

32. post-natal outcome* [tiab] OR post-natal complication* [tiab] OR post-natal consequence* [tiab] OR post-natal characteristic* [tiab] OR post-natal event* [tiab] OR post-natal result* [tiab] OR post-natal problem* [tiab] OR post-natal morbidit* [tiab] OR post-natal sequelae [tiab]

33. antepartum outcome* [tiab] OR antepartum complication* [tiab] OR antepartum consequence* [tiab] OR antepartum characteristic* [tiab] OR antepartum event* [tiab] OR antepartum result* [tiab] OR antepartum problem* [tiab] OR antepartum morbidit* [tiab] OR antepartum sequelae [tiab]

34. ante-partum outcome* [tiab] OR ante-partum complication* [tiab] OR ante-partum consequence* [tiab] OR ante-partum characteristic* [tiab] OR ante-partum event* [tiab] OR ante-partum result* [tiab] OR ante-partum problem* [tiab] OR ante-partum morbidit* [tiab] OR ante-partum sequelae [tiab]

35. intrapartum outcome* [tiab] OR intrapartum complication* [tiab] OR intrapartum consequence* [tiab] OR intrapartum characteristic* [tiab] OR intrapartum event* [tiab] OR intrapartum result* [tiab] OR intrapartum problem* [tiab] OR intrapartum morbidit* [tiab] OR intrapartum sequelae [tiab]

36. intra-partum outcome* [tiab] OR intra-partum complication* [tiab] OR intra-partum consequence* [tiab] OR intra-partum characteristic* [tiab] OR intra-partum event* [tiab] OR intra-partum result* [tiab] OR intra-partum problem* [tiab] OR intra-partum morbidit* [tiab] OR intra-partum sequelae [tiab]

37. peripartum outcome* [tiab] OR peripartum complication* [tiab] OR peripartum consequence* [tiab] OR peripartum characteristic* [tiab] OR peripartum event* [tiab] OR peripartum result* [tiab] OR peripartum problem* [tiab] OR peripartum morbidit* [tiab] OR peripartum sequelae [tiab]

38. peri-partum outcome* [tiab] OR peri-partum complication* [tiab] OR peri-partum consequence* [tiab] OR peri-partum characteristic* [tiab] OR peri-partum event* [tiab] OR peri-partum result* [tiab] OR peri-partum problem* [tiab] OR peri-partum morbidit* [tiab] OR peri-partum sequelae [tiab]

39. postpartum outcome* [tiab] OR postpartum complication* [tiab] OR postpartum consequence* [tiab] OR postpartum characteristic* [tiab] OR postpartum event* [tiab] OR postpartum result* [tiab] OR postpartum problem* [tiab] OR postpartum morbidit* [tiab] OR postpartum sequelae [tiab]

40. post-partum outcome* [tiab] OR post-partum complication* [tiab] OR post-partum consequence* [tiab] OR post-partum characteristic* [tiab] OR post-partum event* [tiab] OR post-partum result* [tiab] OR post-partum problem* [tiab] OR post-partum morbidit* [tiab] OR post-partum sequelae [tiab]

41. #1 or #2 or #3 or #4 or #5 or #6 or #7 or #8 or #9 or #10 or #11 or #12 or #13 or #14 or #15 or #16 or #17 or #18 or #19 or #20 or #21 or #22 or #23 or #24 or #25 or #26 or #27 or #28 or #29 or #30 or #31 or #32 or #33 or #34 or #35 or #36 or #37 or #38 or #39 or #40

42. HIV [Mesh] OR HIV Seropositivity [Mesh] OR HIV Infections [Mesh] OR HIV-2 [Mesh] OR HIV-1 [Mesh] OR AIDS Serodiagnosis [Mesh] OR Acquired Immunodeficiency Syndrome [Mesh] OR AIDS Arteritis, Central Nervous System [Mesh] OR AIDS-Associated Nephropathy [Mesh] OR AIDS Dementia Complex [Mesh] OR AIDS-Related Opportunistic Infections [Mesh] OR Lymphoma, AIDS-Related [Mesh]

43. HIV [tiab] OR HIV-1 [tiab] OR HIV-type-1 [tiab] OR ìHTLV IIIî [tiab] OR HTLV-III [tiab] OR ìHTLV type IIIî [tiab] OR HTLV-type-III [tiab] OR LAV [tiab] OR HTLV-III-LAV [tiab] OR LAV-HTLV-III [tiab] OR HIV-2 [tiab] OR HIV-type-2 [tiab] OR HIV-II [tiab] OR HTLV-IV [tiab] OR LAV-2 [tiab]

44. HIV-positive [tiab] OR HIV-1-positive [tiab] OR HIV-2-positive [tiab] OR HIV-infected [tiab] OR HIV-1-infected [tiab] OR HIV-type-1-infected [tiab] OR ìHTLV III-infectedî [tiab] OR HTLV-III-infected [tiab] OR ìHTLV type III-infectedî [tiab] OR HTLV-type-III-infected [tiab] OR LAV-infected [tiab]

45. HTLV-III-LAV-infected [tiab] OR LAV-HTLV-III-infected [tiab] OR HIV-2-infected [tiab] OR HIV-type-2-infected [tiab] OR HIV-II-infected [tiab] OR HTLV-IV-infected [tiab] OR LAV-2-infected [tiab] OR HIV-infection* [tiab] OR HIV-1-infection* [tiab] OR HIV-type-1-infection* [tiab] OR ìHTLV III-infection*î [tiab] OR HTLV-III-infection* [tiab] OR HTLV type III-infection* [tiab] OR HTLV-type-III-infection* [tiab]

46. LAV-infection* [tiab] OR HTLV-III-LAV-infection* [tiab] OR LAV-HTLV-III-infection* [tiab] OR HIV-2-infection* [tiab] OR HIV-type-2-infection* [tiab] OR HIV-II-infection* [tiab] OR HTLV-IV-infection* [tiab] OR LAV-2-infection* [tiab] OR Human Immunodeficiency Virus* [tiab] OR ìHuman Immune Deficiency Virus*î [tiab]

47. ìHuman T Cell Lymphotropic Virus Type IIIî [tiab] OR ìHuman T-Cell Lymphotropic Virus Type IIIî [tiab] OR ìHuman T Lymphotropic Virus Type IIIî [tiab] OR ìHuman T-Lymphotropic Virus Type IIIî [tiab] OR ìHuman T Lymphotropic Virus Type IVî [tiab] OR ìHuman T-Lymphotropic Virus Type IVî [tiab] OR ìHuman T Cell Leukemia Virus Type IIIî [tiab] OR ìHuman T-Cell Leukemia Virus Type IIIî [tiab]

48. ìLymphadenopathy-Associated Virus*î [tiab] OR ìLymphadenopathy Associated Virus*î [tiab] OR AIDS [tiab] OR ìAcquired Immune Deficiency Syndromeî [tiab] OR ìAcquired Immunodeficiency Syndromeî [tiab]

49. #42 or #43 or #44 or #45 or #46 or #47 or #48

50. Anti-HIV Agents [Mesh] OR HIV Fusion Inhibitors [Mesh] OR HIV Integrase Inhibitors [Mesh] OR HIV Protease Inhibitors [Mesh]

51. antiretrovirals [tiab] OR ìantiretroviral treatment*î [tiab] OR ìantiretroviral therapyî [tiab] OR ìantiretroviral therapiesî [tiab] OR ìantiretroviral regimen*î [tiab] OR ìantiretroviral drug*î [tiab] OR ìantiretroviral agent*î [tiab]

52. anti-retrovirals [tiab] OR ìanti-retroviral treatment*î [tiab] OR ìanti-retroviral therapyî [tiab] OR ìanti-retroviral therapiesî [tiab] OR ìanti-retroviral regimen*î [tiab] OR ìanti-retroviral drug*î [tiab] OR ìanti-retroviral agent*î [tiab]

53. antivirals [tiab] OR ìantiviral treatment*î [tiab] OR ìantiviral therapyî [tiab] OR ìantiviral therapiesî [tiab] OR ìantiviral regimen*î [tiab] OR ìantiviral drug*î [tiab] OR ìantiviral agent*î [tiab]

54. anti-virals [tiab] OR ìanti-viral treatment*î [tiab] OR ìanti-viral therapyî [tiab] OR ìanti-viral therapiesî [tiab] OR ìanti-viral regimen*î [tiab] OR ìanti-viral drug*î [tiab] OR ìanti-viral agent*î [tiab]

55. ìanti-HIV treatment*î [tiab] OR ìanti-HIV therapyî [tiab] OR ìanti-HIV therapiesî [tiab] OR ìanti-HIV regimen*î [tiab] OR ìanti-HIV drug*î [tiab] OR ìanti-HIV agent*î [tiab]

56. ìHIV treatment*î [tiab] OR ìHIV therapyî [tiab] OR ìHIV therapiesî [tiab] OR ìHIV regimenî [tiab] OR ìHIV regimensî [tiab] OR ìHIV drug*î [tiab] OR ìHIV agent*î [tiab]

57. ìanti-HIV-1 treatment*î [tiab] OR ìanti-HIV-1 therapyî [tiab] OR ìanti-HIV-1 therapiesî [tiab] OR ìanti-HIV-1 regimenî [tiab] OR ìanti-HIV-1 regimensî [tiab] OR ìanti-HIV-1 drug*î [tiab] OR ìanti-HIV-1 agent*î [tiab]

58. ìHIV-1 treatment*î [tiab] OR ìHIV-1 therapyî [tiab] OR ìHIV-1 therapiesî [tiab] OR ìHIV-1 regimen*î [tiab] OR ìHIV-1 drug*î [tiab] OR ìHIV-1 agentî [tiab] OR ìHIV-1 agentsî [tiab]

59. ìanti-HIV-2 treatment*î [tiab] OR ìanti-HIV-2 therapyî [tiab] OR ìanti-HIV-2 therapiesî [tiab] OR ìanti-HIV-2 regimen*î [tiab] OR ìanti-HIV-2 drug*î [tiab] OR ìanti-HIV-2 agent*î [tiab] OR ìHIV-2 treatment*î [tiab] OR ìHIV-2 therapyî [tiab] OR ìHIV-2 therapiesî [tiab] OR HIV-2 regimen* [tiab] OR ìHIV-2 drug*î [tiab] OR HIV-2 agent* [tiab]

60. anti-AIDS treatment* [tiab] OR ìanti-AIDS therapyî [tiab] OR ìanti-AIDS therapiesî [tiab] OR anti-AIDS regimen* [tiab] OR ìanti-AIDS drug*î [tiab] OR ìanti-AIDS agent*î [tiab]

61. ìAIDS treatment*î [tiab] OR ìAIDS therapyî [tiab] OR ìAIDS therapiesî [tiab] OR ìAIDS regimen*î [tiab] OR ìAIDS drug*î [tiab] OR ìAIDS agent*î [tiab] OR HAART [tiab] OR HAART-exposed [tiab] OR HAART-treated [tiab] OR Mega-HAART [tiab]

62. ARV [tiab] OR ARVs [tiab] OR cARV [tiab] OR cARVs [tiab] OR ARV-exposed [tiab] OR ARV-treated [tiab] OR combination-ARV [tiab] OR combination-ARVs [tiab] OR combined-ARV [tiab] OR combined-ARVs [tiab]

63. ART [tiab] OR Multi-ART [tiab] OR Triple-ART [tiab] OR cART [tiab] OR ART-exposed [tiab] OR ART-treated [tiab] OR combination-ART [tiab] OR combined-ART [tiab] OR sc-ART [tiab]

64. ìshort-course-antiretroviral therapyî [tiab] OR ìshort-course-antiretroviral therapiesî [tiab] OR ìshort-course-anti-retroviral therapyî [tiab] OR ìshort-course-anti-retroviral therapiesî [tiab]

65. ìcombination treatment*î [tiab] OR ìcombination therapyî [tiab] OR ìcombination therapiesî [tiab] OR ìcombination regimen*î [tiab] OR ìcombination drug*î [tiab] OR ìcombination agent*î [tiab]

66. ìcombined treatment*î [tiab] OR ìcombined therapyî [tiab] OR ìcombined therapiesî [tiab] OR ìcombined regimen*î [tiab] OR ìcombined drug*î [tiab] OR ìcombined agent*î [tiab]

67. monotherapy [tiab] OR monotherapies [tiab] OR mono-therapy [tiab] OR mono-therapies [tiab] OR ìdual therapyî [tiab] OR ìdual therapiesî [tiab] OR ìdual drug therapyî [tiab] OR ìdual drug therapiesî [tiab] OR bitherap* [tiab]

68. PI [tiab] OR PIs [tiab] OR PI-based [tiab] OR PI-boosted [tiab] OR PI-containing [tiab] OR PI-therap* [tiab] OR PI-treatment* [tiab] OR PI-regimen* [tiab] OR Ritonavir-boosted [tiab] OR ìprotease inhibitor*î [tiab] OR NRTI [tiab] OR NRTIs [tiab] OR NRTI-based [tiab]

69. NRTI-containing [tiab] OR NRTI-therap* [tiab] OR NRTI-treatment* [tiab] OR NRTI-regimen* [tiab] OR ìnucleoside reverse transcriptase inhibitor*î [tiab] OR ìnucleoside analog reverse transcriptase inhibitor*î [tiab]

70. NNRTI [tiab] OR NNRTIs [tiab] OR NNRTI-based [tiab] OR NNRTI-containing [tiab] OR NNRTI-therap* [tiab] OR NNRTI-treatment* [tiab] OR NNRTI-regimen* [tiab]

71. ìnon nucleoside reverse transcriptase inhibitor*î [tiab] OR ìnon-nucleoside reverse transcriptase inhibitor*î [tiab] OR ìnonnucleoside reverse transcriptase inhibitor*î [tiab]

72. ìnon nucleoside analog reverse transcriptase inhibitor*î [tiab] OR ìnon-nucleoside analog reverse transcriptase inhibitor*î [tiab] OR ìnonnucleoside analog reverse transcriptase inhibitor*î [tiab]

73. NtRTI [tiab] OR NtRTIs [tiab] OR NtRTI-based [tiab] OR NtRTI-containing [tiab] OR NtRTI-therap* [tiab] OR NtRTI-treatment* [tiab] OR NtRTI-regimen* [tiab] OR ìnucleotide reverse transcriptase inhibitor*î [tiab] OR ìnucleotide analog reverse transcriptase inhibitor*î [tiab]

74. ìfusion inhibitor*î [tiab] OR ìCCR5 receptor antagonist*î [tiab] OR ìintegrase inhibitor*î [tiab] OR ìmaturation inhibitor*î [tiab] OR ìentry inhibitor*î [tiab]

75. Abacavir [tiab] OR ABC [tiab] OR Didanosine [tiab] OR ddI [tiab] OR Emtricitabine [tiab] OR FTC [tiab] OR Lamivudine [tiab] OR 3TC [tiab] OR Stavudine [tiab] OR d4T [tiab] OR Tenofovir [tiab] OR TFV [tiab] OR TDF [tiab]

76. Zidovudine [tiab] OR AZT [tiab] OR ZDV [tiab] OR Delavirdine [tiab] OR DLV [tiab] OR Efavirenz [tiab] OR EFV [tiab] OR Etravirine [tiab] OR ETR [tiab] OR Nevirapine [tiab] OR NVP [tiab] OR Rilpivirine [tiab] OR RPV [tiab]

77. Atazanavir [tiab] OR ATV [tiab] OR Atazanavir/Ritonavir [tiab] OR ATV/r [tiab] OR Darunavir [tiab] OR DRV [tiab] OR Darunavir/Ritonavir [tiab] OR DRV/r [tiab] OR Fosamprenavir [tiab] OR FPV [tiab] OR Fosamprenavir/Ritonavir [tiab] OR FPV/r [tiab]

78. Indinavir [tiab] OR IDV [tiab] OR Indinavir/Ritonavir [tiab] OR IDV/r [tiab] OR Lopinavir [tiab] OR LPV [tiab] OR Lopinavir/Ritonavir [tiab] OR LPV/r [tiab] OR Nelfinavir [tiab] OR NFV [tiab] OR Nelfinavir/Ritonavir [tiab] OR NFV/r [tiab]

79. Ritonavir [tiab] OR RTV [tiab] OR Saquinavir [tiab] OR SQV [tiab] OR Saquinavir/Ritonavir [tiab] OR SQV/r [tiab] OR Tipranavir [tiab] OR TPV [tiab] OR Tipranavir/Ritonavir [tiab] OR TPV/r [tiab] OR Enfuvirtide [tiab] OR T-20 [tiab]

80. Maraviroc [tiab] OR MVC [tiab] OR Rategravir [tiab] OR RAL [tiab] OR Elvitegravir [tiab] OR EVG [tiab] OR Zalcitabine [tiab] OR ddC [tiab] OR Combivir [tiab] OR Trizivir [tiab] OR Kaletra [tiab] OR Epzicom [tiab] OR Kivexa [tiab] OR Truvada [tiab] OR Atripla [tiab]

81. #50 or #51 or #52 or #53 or #54 or #55 or #56 or #57 or #58 or #59 or #60 or #61 or #62 or #63 or #64 or #65 or #66 or #67 or #68 or #69 or #70 or #71 or #72 or #73 or #74 or #75 or #76 or #77 or #78 or #79 or #80

82. #49 OR #81

83. #41 AND #82

84. 2018/04/29:2020/04/20 [dp]

85. #83 AND #84

Search strategy for “specific perinatal outcomes AND HIV OR antiretroviral therapy”

Database and platform: PubMed (via https://www.ncbi.nlm.nih.gov/pubmed)

Latest search date: 20 April 2020.

1. Premature Birth [Mesh] OR Fetal Membranes, Premature Rupture [Mesh] OR Obstetric Labor, Premature [Mesh] OR Infant, Extremely Premature [Mesh] OR Infant, Premature [Mesh]

2. prematurity [tiab] OR ìgestational age at birthî [tiab] OR ìgestational age at deliveryî [tiab] OR PTB [tiab] OR PTBs [tiab] OR VPTB [tiab] OR VPTBs [tiab]

3. pre-terms [tiab] OR preterms [tiab] OR pre-term birth* [tiab] OR ìpreterm birth*î [tiab] OR ìpremature birth*î [tiab] OR PTL [tiab] OR PTLs [tiab] OR VPTL [tiab] OR VPTLs [tiab]

4. ìpre-term labor*î [tiab] OR ìpre-term labour*î [tiab] OR ìpre-term obstetric labor*î [tiab] OR ìpre-term obstetric labour*î [tiab] OR ìpreterm labor*î [tiab] OR ìpreterm labour*î [tiab] OR preterm obstetric labor* [tiab] OR ìpreterm obstetric labour*î [tiab] OR ìpremature labor*î [tiab] OR ìpremature labour*î [tiab] OR premature obstetric labor* [tiab] OR ìpremature obstetric labour*î [tiab]

5. PTD [tiab] OR PTDs [tiab] OR VPTD [tiab] OR VPTDs [tiab] OR pre-term deliver* [tiab] OR preterm deliver* [tiab] OR ìpre-term infant*î [tiab] OR ìpreterm infant*î [tiab] OR premature deliver* [tiab] OR ìpremature infant*î [tiab] OR PROM [tiab] OR PPROM [tiab]

6. ìpreterm rupture of membranesî [tiab] OR preterm rupture of fetal membrane* [tiab] OR preterm rupture of foetal membrane* [tiab] OR pre-term rupture of membrane* [tiab] OR pre-term rupture of fetal membrane* [tiab] OR pre-term rupture of foetal membrane* [tiab] OR ìpremature rupture of membranesî [tiab] OR ìpremature rupture of fetal membranesî [tiab] OR ìpremature rupture of foetal membranesî [tiab]

7. #1 or #2 or #3 or #4 or #5 or #6

8. Fetal Growth Retardation [Mesh] OR Infant, Low Birth Weight [Mesh] OR Infant, Very Low Birth Weight [Mesh] OR Infant, Extremely Low Birth Weight [Mesh] OR Infant, Small for Gestational Age [Mesh]

9. IUGR [tiab] OR FGR [tiab] OR ìintrauterine growth restrictionî [tiab] OR ìintra-uterine growth restrictionî [tiab] OR ìintrauterine growth restrictedî [tiab] OR ìintra-uterine growth restrictedî [tiab] OR ìintrauterine growth retardationî [tiab] OR ìintra-uterine growth retardationî [tiab]

10. ìfetal growth restrictionî [tiab] OR ìfoetal growth restrictionî [tiab] OR ìfetal growth restrictedî [tiab] OR ìfoetal growth restrictedî [tiab] OR ìfetal growth retardationî [tiab] OR ìfoetal growth retardationî [tiab] OR SGA [tiab] OR SFGA [tiab] OR ìsmall for gestational ageî [tiab] OR ìsmall-for-gestational-ageî [tiab] OR ìsmall-for-gestational ageî [tiab] OR ìsmall for gestationî [tiab] OR ìsmall-for-gestationî [tiab]

11. VSGA [tiab] OR ìvery-small-for-gestational-ageî [tiab] OR ìvery-small-for-gestational ageî [tiab] OR SFD [tiab] OR ìsmall for datesî [tiab] OR ìsmall-for-datesî [tiab] OR ìweight for datesî [tiab] OR ìweight for gestational ageî [tiab] OR ìweight for age at deliveryî [tiab] OR ìweight at deliveryî [tiab]

12. ìbirthweight for datesî [tiab] OR ìbirthweight for gestational ageî [tiab] OR ìbirthweight for age at deliveryî [tiab] OR ìbirth weight for datesî [tiab] OR ìbirth weight for gestational ageî [tiab] OR ìbirth weight for age at deliveryî [tiab] OR ìbirth-weight for datesî [tiab] OR ìbirth-weight for gestational ageî [tiab] OR ìbirth-weight for age at deliveryî [tiab]

13. LBW [tiab] OR ìlow BWî [tiab] OR ìlow birth weightî [tiab] OR ìlow birth-weightî [tiab] OR ìlow-birth weightî [tiab] OR ìlow-birth-weightî [tiab] OR ìlow birthweightî [tiab] OR ìlow-birthweightî [tiab] OR ìlower BWî [tiab] OR ìlower birth weightî [tiab] OR ìlower birth-weightî [tiab] OR ìlower-birth weightî [tiab] OR ìlower-birth-weightî [tiab] OR ìlower birthweightî [tiab] OR ìlower-birthweightî [tiab]

14. ìreduced birth weightî [tiab] OR ìreduced birthweightî [tiab] OR ìreduced birth-weightî [tiab] OR VLBW [tiab] OR ìvery-low birthweightî [tiab] OR ìvery-low birth weightî [tiab] OR ìvery-low birth-weightî [tiab] OR ìvery-low-birthweightî [tiab] OR ìvery-low-birth-weightî [tiab] OR ELBW [tiab] OR ìextremely-low birthweightî [tiab] OR ìextremely-low birth weightî [tiab] OR ìextremely-low birth-weightî [tiab] OR ìextremely-low-birthweightî [tiab] OR ìextremely-low-birth-weightî [tiab]

15. #8 or #9 or #10 or #11 or #12 or #13 or #14

16. Stillbirth [Mesh] OR stillbirth* [tiab] OR still birth* [tiab] OR stillborn* [tiab] OR still born* [tiab] OR abortion* [tiab] OR miscarriage* [tiab] OR pregnancy death* [tiab] OR pregnancy loss* [tiab] OR pregnancy demise* [tiab] OR pregnancy mortalit* [tiab] OR gestational death* [tiab] OR gestational loss* [tiab] OR gestational demise* [tiab] OR "gestational mortalit*" [tiab]

17. Fetal Death [Mesh] OR "fetal death*" [tiab] OR "fetal loss*" [tiab] OR fetal demise* [tiab] OR fetal mortalit* [tiab] OR "foetal death*" [tiab] OR "foetal loss*" [tiab] OR foetal demise* [tiab] OR foetal mortalit* [tiab] OR "obstetric death*" [tiab] OR obstetric loss* [tiab] OR obstetric demise* [tiab] OR obstetric mortalit* [tiab] OR obstetrical death* [tiab] OR obstetrical loss* [tiab] OR obstetrical demise* [tiab] OR obstetrical mortalit* [tiab]

18. "labor death*" [tiab] OR labor loss* [tiab] OR labor demise* [tiab] OR labor mortalit* [tiab] OR labour death* [tiab] OR labour loss* [tiab] OR labour demise* [tiab] OR labour mortalit* [tiab] OR birth death* [tiab] OR birth loss* [tiab] OR birth demise* [tiab] OR birth mortalit* [tiab]

19. delivery death* [tiab] OR delivery loss* [tiab] OR delivery demise* [tiab] OR delivery mortalit* [tiab] OR neonate death* [tiab] OR neonate loss* [tiab] OR neonate demise* [tiab] OR neonate mortalit* [tiab] OR "newborn death*" [tiab] OR "newborn loss*" [tiab] OR "newborn demise*" [tiab] OR newborn mortalit* [tiab]

20. new-born death* [tiab] OR new-born loss* [tiab] OR new-born demise* [tiab] OR new-born mortalit* [tiab] OR "new born death*" [tiab] OR "new born loss*" [tiab] OR "new born demise*" [tiab] OR "new born mortalit*" [tiab] OR "infant death*" [tiab] OR infant loss* [tiab] OR infant demise* [tiab] OR infant mortalit* [tiab] OR reproductive death* [tiab] OR "reproductive loss*" [tiab] OR reproductive demise* [tiab] OR reproductive mortalit* [tiab]

21. prelabour death* [tiab] OR prelabour loss* [tiab] OR prelabour demise* [tiab] OR prelabour mortalit* [tiab] OR prelabor death* [tiab] OR prelabor loss* [tiab] OR prelabor demise* [tiab] OR prelabor mortalit* [tiab] OR pre-labour death* [tiab] OR pre-labour loss* [tiab] OR pre-labour demise* [tiab] OR pre-labour mortalit* [tiab] OR pre-labor death* [tiab] OR pre-labor loss* [tiab] OR pre-labor demise* [tiab] OR pre-labor mortalit* [tiab]

22. "intrauterine death*" [tiab] OR intrauterine loss* [tiab] OR intrauterine demise* [tiab] OR intrauterine mortalit* [tiab] OR intra-uterine death* [tiab] OR intra-uterine loss* [tiab] OR intra-uterine demise* [tiab] OR intra-uterine mortalit* [tiab] OR antenatal death* [tiab] OR antenatal loss* [tiab] OR antenatal demise* [tiab] OR antenatal mortalit* [tiab] OR ante-natal death* [tiab] OR ante-natal loss* [tiab] OR ante-natal demise* [tiab] OR ante-natal mortalit* [tiab]

23. prenatal death* [tiab] OR prenatal loss* [tiab] OR prenatal demise* [tiab] OR prenatal mortalit* [tiab] OR pre-natal death* [tiab] OR pre-natal loss* [tiab] OR pre-natal demise* [tiab] OR pre-natal mortalit* [tiab] OR "perinatal death" [tiab] OR "perinatal deaths" [tiab] OR perinatal loss* [tiab] OR perinatal demise* [tiab] OR "perinatal mortality" [tiab] OR "perinatal mortalities" [tiab] OR peri-natal death* [tiab] OR peri-natal loss* [tiab] OR peri-natal demise* [tiab] OR peri-natal mortalit* [tiab]

24. "neonatal death" [tiab] OR "neonatal deaths" [tiab] OR neonatal loss* [tiab] OR neonatal demise* [tiab] OR "neonatal mortality" [tiab] OR "neonatal mortalities" [tiab] OR neo-natal death* [tiab] OR neo-natal loss* [tiab] OR neo-natal demise* [tiab] OR neo-natal mortalit* [tiab] OR postnatal death* [tiab] OR postnatal loss* [tiab] OR postnatal demise* [tiab] OR postnatal mortalit* [tiab] OR post-

natal death* [tiab] OR post-natal loss* [tiab] OR post-natal demise* [tiab] OR post-natal mortalit* [tiab]

25. antepartum death* [tiab] OR antepartum loss* [tiab] OR antepartum demise* [tiab] OR antepartum mortalit* [tiab] OR ante-partum death* [tiab] OR ante-partum loss* [tiab] OR ante-partum demise* [tiab] OR ante-partum mortalit* [tiab] OR intrapartum death* [tiab] OR intrapartum loss* [tiab] OR intrapartum demise* [tiab] OR intrapartum mortalit* [tiab] OR intra-partum death* [tiab] OR intra-partum loss* [tiab] OR intra-partum demise* [tiab] OR intra-partum mortalit* [tiab]

26. peripartum death* [tiab] OR peripartum loss* [tiab] OR peripartum demise* [tiab] OR peripartum mortalit* [tiab] OR peri-partum death* [tiab] OR peri-partum loss* [tiab] OR peri-partum demise* [tiab] OR peri-partum mortalit* [tiab] OR postpartum death* [tiab] OR postpartum loss* [tiab] OR postpartum demise* [tiab] OR postpartum mortalit* [tiab] OR post-partum death* [tiab] OR post-partum loss* [tiab] OR post-partum demise* [tiab] OR post-partum mortalit* [tiab]

27. #16 or #17 or #18 or #19 or #20 or #21 or #22 or #23 or #24 or #25 or #26

28. HIV [Mesh] OR HIV Seropositivity [Mesh] OR HIV Infections [Mesh] OR HIV-2 [Mesh] OR HIV-1 [Mesh] OR AIDS Serodiagnosis [Mesh] OR Acquired Immunodeficiency Syndrome [Mesh] OR AIDS Arteritis, Central Nervous System [Mesh] OR AIDS-Associated Nephropathy [Mesh] OR AIDS Dementia Complex [Mesh] OR AIDS-Related Opportunistic Infections [Mesh] OR Lymphoma, AIDS-Related [Mesh]

29. HIV [tiab] OR HIV-1 [tiab] OR HIV-type-1 [tiab] OR ìHTLV IIIî [tiab] OR HTLV-III [tiab] OR ìHTLV type IIIî [tiab] OR HTLV-type-III [tiab] OR LAV [tiab] OR HTLV-III-LAV [tiab] OR LAV-HTLV-III [tiab] OR HIV-2 [tiab] OR HIV-type-2 [tiab] OR HIV-II [tiab] OR HTLV-IV [tiab] OR LAV-2 [tiab]

30. HIV-positive [tiab] OR HIV-1-positive [tiab] OR HIV-2-positive [tiab] OR HIV-infected [tiab] OR HIV-1-infected [tiab] OR HIV-type-1-infected [tiab] OR ìHTLV III-infectedî [tiab] OR HTLV-III-infected [tiab] OR ìHTLV type III-infectedî [tiab] OR HTLV-type-III-infected [tiab] OR LAV-infected [tiab]

31. HTLV-III-LAV-infected [tiab] OR LAV-HTLV-III-infected [tiab] OR HIV-2-infected [tiab] OR HIV-type-2-infected [tiab] OR HIV-II-infected [tiab] OR HTLV-IV-infected [tiab] OR LAV-2-infected [tiab] OR HIV-infection* [tiab] OR HIV-1-infection* [tiab] OR HIV-type-1-infection* [tiab] OR ìHTLV III-infection*î [tiab] OR HTLV-III-infection* [tiab] OR HTLV type III-infection* [tiab] OR HTLV-type-III-infection* [tiab]

32. LAV-infection* [tiab] OR HTLV-III-LAV-infection* [tiab] OR LAV-HTLV-III-infection* [tiab] OR HIV-2-infection* [tiab] OR HIV-type-2-infection* [tiab] OR HIV-II-infection* [tiab] OR HTLV-IV-infection* [tiab] OR LAV-2-infection* [tiab] OR Human Immunodeficiency Virus* [tiab] OR ìHuman Immune Deficiency Virus*î [tiab]

33. ìHuman T Cell Lymphotropic Virus Type IIIî [tiab] OR ìHuman T-Cell Lymphotropic Virus Type IIIî [tiab] OR ìHuman T Lymphotropic Virus Type IIIî [tiab] OR ìHuman T-Lymphotropic Virus Type IIIî [tiab] OR ìHuman T Lymphotropic Virus Type IVî [tiab] OR ìHuman T-Lymphotropic Virus Type IVî [tiab] OR ìHuman T Cell Leukemia Virus Type IIIî [tiab] OR ìHuman T-Cell Leukemia Virus Type IIIî [tiab]

34. ìLymphadenopathy-Associated Virus*î [tiab] OR ìLymphadenopathy Associated Virus*î [tiab] OR AIDS [tiab] OR ìAcquired Immune Deficiency Syndromeî [tiab] OR ìAcquired Immunodeficiency Syndromeî [tiab]

35. #28 or #29 or #30 or #31 or #32 or #33 or #34

36. Anti-HIV Agents [Mesh] OR HIV Fusion Inhibitors [Mesh] OR HIV Integrase Inhibitors [Mesh] OR HIV Protease Inhibitors [Mesh]

37. antiretrovirals [tiab] OR ìantiretroviral treatment*î [tiab] OR ìantiretroviral therapyî [tiab] OR ìantiretroviral therapiesî [tiab] OR ìantiretroviral regimen*î [tiab] OR ìantiretroviral drug*î [tiab] OR ìantiretroviral agent*î [tiab]

38. anti-retrovirals [tiab] OR ìanti-retroviral treatment*î [tiab] OR ìanti-retroviral therapyî [tiab] OR ìanti-retroviral therapiesî [tiab] OR ìanti-retroviral regimen*î [tiab] OR ìanti-retroviral drug*î [tiab] OR ìanti-retroviral agent*î [tiab]

39. antivirals [tiab] OR ìantiviral treatment*î [tiab] OR ìantiviral therapyî [tiab] OR ìantiviral therapiesî [tiab] OR ìantiviral regimen*î [tiab] OR ìantiviral drug*î [tiab] OR ìantiviral agent*î [tiab]

40. anti-virals [tiab] OR ìanti-viral treatment*î [tiab] OR ìanti-viral therapyî [tiab] OR ìanti-viral therapiesî [tiab] OR ìanti-viral regimen*î [tiab] OR ìanti-viral drug*î [tiab] OR ìanti-viral agent*î [tiab]

41. ìanti-HIV treatment*î [tiab] OR ìanti-HIV therapyî [tiab] OR ìanti-HIV therapiesî [tiab] OR ìanti-HIV regimen*î [tiab] OR ìanti-HIV drug*î [tiab] OR ìanti-HIV agent*î [tiab]

42. ìHIV treatment*î [tiab] OR ìHIV therapyî [tiab] OR ìHIV therapiesî [tiab] OR ìHIV regimenî [tiab] OR ìHIV regimensî [tiab] OR ìHIV drug*î [tiab] OR ìHIV agent*î [tiab]

43. ìanti-HIV-1 treatment*î [tiab] OR ìanti-HIV-1 therapyî [tiab] OR ìanti-HIV-1 therapiesî [tiab] OR ìanti-HIV-1 regimenî [tiab] OR ìanti-HIV-1 regimensî [tiab] OR ìanti-HIV-1 drug*î [tiab] OR ìanti-HIV-1 agent*î [tiab]

44. ìHIV-1 treatment*î [tiab] OR ìHIV-1 therapyî [tiab] OR ìHIV-1 therapiesî [tiab] OR ìHIV-1 regimen*î [tiab] OR ìHIV-1 drug*î [tiab] OR ìHIV-1 agentî [tiab] OR ìHIV-1 agentsî [tiab]

45. ìanti-HIV-2 treatment*î [tiab] OR ìanti-HIV-2 therapyî [tiab] OR ìanti-HIV-2 therapiesî [tiab] OR ìanti-HIV-2 regimen*î [tiab] OR ìanti-HIV-2 drug*î [tiab] OR ìanti-HIV-2 agent*î [tiab] OR ìHIV-2 treatment*î [tiab] OR ìHIV-2 therapyî [tiab] OR ìHIV-2 therapiesî [tiab] OR HIV-2 regimen* [tiab] OR ìHIV-2 drug*î [tiab] OR HIV-2 agent* [tiab]

46. anti-AIDS treatment* [tiab] OR ìanti-AIDS therapyî [tiab] OR ìanti-AIDS therapiesî [tiab] OR anti-AIDS regimen* [tiab] OR ìanti-AIDS drug*î [tiab] OR ìanti-AIDS agent*î [tiab]

47. ìAIDS treatment*î [tiab] OR ìAIDS therapyî [tiab] OR ìAIDS therapiesî [tiab] OR ìAIDS regimen*î [tiab] OR ìAIDS drug*î [tiab] OR ìAIDS agent*î [tiab] OR HAART [tiab] OR HAART-exposed [tiab] OR HAART-treated [tiab] OR Mega-HAART [tiab]

48. ARV [tiab] OR ARVs [tiab] OR cARV [tiab] OR cARVs [tiab] OR ARV-exposed [tiab] OR ARV-treated [tiab] OR combination-ARV [tiab] OR combination-ARVs [tiab] OR combined-ARV [tiab] OR combined-ARVs [tiab]

49. ART [tiab] OR Multi-ART [tiab] OR Triple-ART [tiab] OR cART [tiab] OR ART-exposed [tiab] OR ART-treated [tiab] OR combination-ART [tiab] OR combined-ART [tiab] OR sc-ART [tiab]

50. ìshort-course-antiretroviral therapyî [tiab] OR ìshort-course-antiretroviral therapiesî [tiab] OR ìshort-course-anti-retroviral therapyî [tiab] OR ìshort-course-anti-retroviral therapiesî [tiab]

51. ìcombination treatment*î [tiab] OR ìcombination therapyî [tiab] OR ìcombination therapiesî [tiab] OR ìcombination regimen*î [tiab] OR ìcombination drug*î [tiab] OR ìcombination agent*î [tiab]

52. ìcombined treatment*î [tiab] OR ìcombined therapyî [tiab] OR ìcombined therapiesî [tiab] OR ìcombined regimen*î [tiab] OR ìcombined drug*î [tiab] OR ìcombined agent*î [tiab]

53. monotherapy [tiab] OR monotherapies [tiab] OR mono-therapy [tiab] OR mono-therapies [tiab] OR ìdual therapyî [tiab] OR ìdual therapiesî [tiab] OR ìdual drug therapyî [tiab] OR ìdual drug therapiesî [tiab] OR bitherap* [tiab]

54. PI [tiab] OR PIs [tiab] OR PI-based [tiab] OR PI-boosted [tiab] OR PI-containing [tiab] OR PI-therap* [tiab] OR PI-treatment* [tiab] OR PI-regimen* [tiab] OR Ritonavir-boosted [tiab] OR ìprotease inhibitor*î [tiab] OR NRTI [tiab] OR NRTIs [tiab] OR NRTI-based [tiab]

55. NRTI-containing [tiab] OR NRTI-therap* [tiab] OR NRTI-treatment* [tiab] OR NRTI-regimen* [tiab] OR ìnucleoside reverse transcriptase inhibitor*î [tiab] OR ìnucleoside analog reverse transcriptase inhibitor*î [tiab]

56. NNRTI [tiab] OR NNRTIs [tiab] OR NNRTI-based [tiab] OR NNRTI-containing [tiab] OR NNRTI-therap* [tiab] OR NNRTI-treatment* [tiab] OR NNRTI-regimen* [tiab]

57. ìnon nucleoside reverse transcriptase inhibitor*î [tiab] OR ìnon-nucleoside reverse transcriptase inhibitor*î [tiab] OR ìnonnucleoside reverse transcriptase inhibitor*î [tiab]

58. ìnon nucleoside analog reverse transcriptase inhibitor*î [tiab] OR ìnon-nucleoside analog reverse transcriptase inhibitor*î [tiab] OR ìnonnucleoside analog reverse transcriptase inhibitor*î [tiab]

59. NtRTI [tiab] OR NtRTIs [tiab] OR NtRTI-based [tiab] OR NtRTI-containing [tiab] OR NtRTI-therap* [tiab] OR NtRTI-treatment* [tiab] OR NtRTI-regimen* [tiab] OR ìnucleotide reverse transcriptase inhibitor*î [tiab] OR ìnucleotide analog reverse transcriptase inhibitor*î [tiab]

60. ìfusion inhibitor*î [tiab] OR ìCCR5 receptor antagonist*î [tiab] OR ìintegrase inhibitor*î [tiab] OR ìmaturation inhibitor*î [tiab] OR ìentry inhibitor*î [tiab]

61. Abacavir [tiab] OR ABC [tiab] OR Didanosine [tiab] OR ddI [tiab] OR Emtricitabine [tiab] OR FTC [tiab] OR Lamivudine [tiab] OR 3TC [tiab] OR Stavudine [tiab] OR d4T [tiab] OR Tenofovir [tiab] OR TFV [tiab] OR TDF [tiab]

62. Zidovudine [tiab] OR AZT [tiab] OR ZDV [tiab] OR Delavirdine [tiab] OR DLV [tiab] OR Efavirenz [tiab] OR EFV [tiab] OR Etravirine [tiab] OR ETR [tiab] OR Nevirapine [tiab] OR NVP [tiab] OR Rilpivirine [tiab] OR RPV [tiab]

63. Atazanavir [tiab] OR ATV [tiab] OR Atazanavir/Ritonavir [tiab] OR ATV/r [tiab] OR Darunavir [tiab] OR DRV [tiab] OR Darunavir/Ritonavir [tiab] OR DRV/r [tiab] OR Fosamprenavir [tiab] OR FPV [tiab] OR Fosamprenavir/Ritonavir [tiab] OR FPV/r [tiab]

64. Indinavir [tiab] OR IDV [tiab] OR Indinavir/Ritonavir [tiab] OR IDV/r [tiab] OR Lopinavir [tiab] OR LPV [tiab] OR Lopinavir/Ritonavir [tiab] OR LPV/r [tiab] OR Nelfinavir [tiab] OR NFV [tiab] OR Nelfinavir/Ritonavir [tiab] OR NFV/r [tiab]

65. Ritonavir [tiab] OR RTV [tiab] OR Saquinavir [tiab] OR SQV [tiab] OR Saquinavir/Ritonavir [tiab] OR SQV/r [tiab] OR Tipranavir [tiab] OR TPV [tiab] OR Tipranavir/Ritonavir [tiab] OR TPV/r [tiab] OR Enfuvirtide [tiab] OR T-20 [tiab]

66. Maraviroc [tiab] OR MVC [tiab] OR Raltegravir [tiab] OR RAL [tiab] OR Elvitegravir [tiab] OR EVG [tiab] OR Zalcitabine [tiab] OR ddC [tiab] OR Combivir [tiab] OR Trizivir [tiab] OR Kaletra [tiab] OR Epzicom [tiab] OR Kivexa [tiab] OR Truvada [tiab] OR Atripla [tiab]

67. #36 or #37 or #38 or #39 or #40 or #41 or #42 or #43 or #44 or #45 or #46 or #47 or #48 or #49 or #50 or #51 or #52 or #53 or #54 or #55 or #56 or #57 or #58 or #59 or #60 or #61 or #62 or #63 or #64 or #65 or #66

68. #7 OR #15 OR #27

69. #35 OR #67

70. #68 AND #69

71. 2018/04/29:2020/04/20 [dp]

72. #70 AND #71

Supplementary literature search for “pregnancy outcome OR specific perinatal outcomes AND new antiretroviral therapy”

Database and platform: Pubmed (via https://www.ncbi.nlm.nih.gov/pubmed/)

Latest search date: 20 April 2020.

1. Pregnancy Outcome [Mesh] OR Pregnancy Complications, Infectious [Mesh] OR "pregnancy outcome*" [tiab] OR "pregnancy complication*" [tiab] OR pregnancy consequence* [tiab] OR "pregnancy characteristic" [tiab] OR "pregnancy characteristics" [tiab] OR pregnancy event* [tiab] OR pregnancy result* [tiab] OR pregnancy problem* [tiab] OR pregnancy morbidit* [tiab] OR pregnancy sequelae [tiab]

2. gestational outcome* [tiab] OR gestational complication* [tiab] OR gestational consequence* [tiab] OR gestational characteristic* [tiab] OR gestational event* [tiab] OR gestational result* [tiab] OR gestational problem* [tiab] OR gestational morbidit* [tiab] OR gestational sequelae [tiab]

3. "fetal outcome" [tiab] OR "fetal outcomes" [tiab] OR "fetal complication" [tiab] OR "fetal complications" [tiab] OR fetal consequence* [tiab] OR fetal characteristic* [tiab] OR fetal event* [tiab] OR fetal result* [tiab] OR fetal problem* [tiab] OR fetal morbidit* [tiab] OR fetal sequelae [tiab]

4. foetal outcome* [tiab] OR foetal complication* [tiab] OR foetal consequence* [tiab] OR foetal characteristic* [tiab] OR foetal event* [tiab] OR foetal result* [tiab] OR foetal problem* [tiab] OR foetal morbidit* [tiab] OR foetal sequelae [tiab]

5. "obstetric outcome" [tiab] OR "obstetric outcomes" [tiab] OR "obstetric complication" [tiab] OR "obstetric complications" [tiab] OR obstetric consequence* [tiab] OR obstetric characteristic* [tiab] OR obstetric event* [tiab] OR obstetric result* [tiab] OR "obstetric problem" [tiab] OR "obstetric problems" [tiab] OR obstetric morbidit* [tiab] OR obstetric sequelae [tiab]

6. obstetrical outcome* [tiab] OR "obstetrical complication" [tiab] OR "obstetrical complications" [tiab] OR obstetrical consequence* [tiab] OR obstetrical characteristic* [tiab] OR obstetrical event* [tiab] OR obstetrical result* [tiab] OR "obstetrical problem" [tiab] OR "obstetrical problems" [tiab] OR obstetrical morbidit* [tiab] OR obstetrical sequelae [tiab]

7. "labor outcome" [tiab] OR "labor outcomes" [tiab] OR "labor complication" [tiab] OR "labor complications" [tiab] OR "labor consequence" [tiab] OR "labor consequences" [tiab] OR labor characteristic* [tiab] OR labor event* [tiab] OR labor result* [tiab] OR labor problem* [tiab] OR labor morbidit* [tiab] OR labor sequelae [tiab]

8. labour outcome* [tiab] OR labour complication* [tiab] OR "labour consequence" [tiab] OR "labour consequences" [tiab] OR labour characteristic* [tiab] OR labour event* [tiab] OR labour result* [tiab] OR labour problem* [tiab] OR labour morbidit* [tiab] OR labour sequelae [tiab]

9. "birth outcome" [tiab] OR "birth outcomes" [tiab] OR birth complication* [tiab] OR birth consequence* [tiab] OR birth characteristic* [tiab] OR birth event* [tiab] OR birth result* [tiab] OR birth problem* [tiab] OR birth morbidit* [tiab] OR birth sequelae [tiab]

10. delivery outcome* [tiab] OR delivery complication* [tiab] OR delivery consequence* [tiab] OR delivery characteristic* [tiab] OR delivery event* [tiab] OR delivery result* [tiab] OR delivery problem* [tiab] OR delivery morbidit* [tiab] OR delivery sequelae [tiab]

11. neonate outcome* [tiab] OR neonate complication* [tiab] OR neonate consequence* [tiab] OR neonate characteristic* [tiab] OR neonate event* [tiab] OR neonate result* [tiab] OR neonate problem* [tiab] OR neonate morbidit* [tiab] OR neonate sequelae [tiab]

12. newborn outcome* [tiab] OR newborn complication* [tiab] OR newborn consequence* [tiab] OR newborn characteristic* [tiab] OR newborn event* [tiab] OR newborn result* [tiab] OR newborn problem* [tiab] OR newborn morbidit* [tiab] OR newborn sequelae [tiab]

13. new-born outcome* [tiab] OR new-born complication* [tiab] OR new-born consequence* [tiab] OR new-born characteristic* [tiab] OR new-born event* [tiab] OR new-born result* [tiab] OR new-born problem* [tiab] OR new-born morbidit* [tiab] OR new-born sequelae [tiab]

14. new born outcome* [tiab] OR new born complication* [tiab] OR new born consequence* [tiab] OR new born characteristic* [tiab] OR new born event* [tiab] OR new born result* [tiab] OR new born problem* [tiab] OR new born morbidit* [tiab] OR new born sequelae [tiab]

15. infant outcome* [tiab] OR infant complication* [tiab] OR infant consequence* [tiab] OR infant characteristic* [tiab] OR infant event* [tiab] OR infant result* [tiab] OR infant problem* [tiab] OR infant morbidit* [tiab] OR infant sequelae [tiab]

16. reproductive outcome* [tiab] OR reproductive complication* [tiab] OR reproductive consequence* [tiab] OR reproductive characteristic* [tiab] OR reproductive event* [tiab] OR reproductive result* [tiab] OR reproductive problem* [tiab] OR reproductive morbidit* [tiab] OR reproductive sequelae [tiab]

17. prelabour outcome* [tiab] OR prelabour complication* [tiab] OR prelabour consequence* [tiab] OR prelabour characteristic* [tiab] OR prelabour event* [tiab] OR prelabour result* [tiab] OR prelabour problem* [tiab] OR prelabour morbidit* [tiab] OR prelabour sequelae [tiab]

18. prelabor outcome* [tiab] OR prelabor complication* [tiab] OR prelabor consequence* [tiab] OR prelabor characteristic* [tiab] OR prelabor event* [tiab] OR prelabor result* [tiab] OR prelabor problem* [tiab] OR prelabor morbidit* [tiab] OR prelabor sequelae [tiab]

19. pre-labour outcome* [tiab] OR pre-labour complication* [tiab] OR pre-labour consequence* [tiab] OR pre-labour characteristic* [tiab] OR pre-labour event* [tiab] OR pre-labour result* [tiab] OR pre-labour problem* [tiab] OR pre-labour morbidit* [tiab] OR pre-labour sequelae [tiab]

20. pre-labor outcome* [tiab] OR pre-labor complication* [tiab] OR pre-labor consequence* [tiab] OR pre-labor characteristic* [tiab] OR pre-labor event* [tiab] OR pre-labor result* [tiab] OR pre-labor problem* [tiab] OR pre-labor morbidit* [tiab] OR pre-labor sequelae [tiab]

21. intrauterine outcome* [tiab] OR intrauterine complication* [tiab] OR intrauterine consequence* [tiab] OR intrauterine characteristic* [tiab] OR intrauterine event* [tiab] OR intrauterine result* [tiab] OR intrauterine problem* [tiab] OR intrauterine morbidit* [tiab] OR intrauterine sequelae [tiab]

22. intra-uterine outcome* [tiab] OR intra-uterine complication* [tiab] OR intra-uterine consequence* [tiab] OR intra-uterine characteristic* [tiab] OR intra-uterine event* [tiab] OR intra-uterine result* [tiab] OR intra-uterine problem* [tiab] OR intra-uterine morbidit* [tiab] OR intra-uterine sequelae [tiab]

23. antenatal outcome* [tiab] OR antenatal complication* [tiab] OR antenatal consequence* [tiab] OR antenatal characteristic* [tiab] OR antenatal event* [tiab] OR antenatal result* [tiab] OR antenatal problem* [tiab] OR antenatal morbidit* [tiab] OR antenatal sequelae [tiab]

24. ante-natal outcome* [tiab] OR ante-natal complication* [tiab] OR ante-natal consequence* [tiab] OR ante-natal characteristic* [tiab] OR ante-natal event* [tiab] OR ante-natal result* [tiab] OR

ante-natal problem* [tiab] OR ante-natal morbidit* [tiab] OR ante-natal sequelae [tiab]

25. prenatal outcome* [tiab] OR prenatal complication* [tiab] OR prenatal consequence* [tiab] OR prenatal characteristic* [tiab] OR prenatal event* [tiab] OR prenatal result* [tiab] OR prenatal problem* [tiab] OR prenatal morbidit* [tiab] OR prenatal sequelae [tiab]

26. pre-natal outcome* [tiab] OR pre-natal complication* [tiab] OR pre-natal consequence* [tiab] OR pre-natal characteristic* [tiab] OR pre-natal event* [tiab] OR pre-natal result* [tiab] OR pre-natal problem* [tiab] OR pre-natal morbidit* [tiab] OR pre-natal sequelae [tiab]

27. perinatal outcome* [tiab] OR perinatal complication* [tiab] OR perinatal consequence* [tiab] OR perinatal characteristic* [tiab] OR perinatal event* [tiab] OR perinatal result* [tiab] OR perinatal problem* [tiab] OR perinatal morbidit* [tiab] OR perinatal sequelae [tiab]

28. peri-natal outcome* [tiab] OR peri-natal complication* [tiab] OR peri-natal consequence* [tiab] OR peri-natal characteristic* [tiab] OR peri-natal event* [tiab] OR peri-natal result* [tiab] OR peri-natal problem* [tiab] OR peri-natal morbidit* [tiab] OR peri-natal sequelae [tiab]

29. neonatal outcome* [tiab] OR neonatal complication* [tiab] OR neonatal consequence* [tiab] OR neonatal characteristic* [tiab] OR neonatal event* [tiab] OR neonatal result* [tiab] OR neonatal problem* [tiab] OR neonatal morbidit* [tiab] OR neonatal sequelae [tiab]

30. neo-natal outcome* [tiab] OR neo-natal complication* [tiab] OR neo-natal consequence* [tiab] OR neo-natal characteristic* [tiab] OR neo-natal event* [tiab] OR neo-natal result* [tiab] OR neo-natal problem* [tiab] OR neo-natal morbidit* [tiab] OR neo-natal sequelae [tiab]

31. postnatal outcome* [tiab] OR postnatal complication* [tiab] OR postnatal consequence* [tiab] OR postnatal characteristic* [tiab] OR postnatal event* [tiab] OR postnatal result* [tiab] OR postnatal problem* [tiab] OR postnatal morbidit* [tiab] OR postnatal sequelae [tiab]

32. post-natal outcome* [tiab] OR post-natal complication* [tiab] OR post-natal consequence* [tiab] OR post-natal characteristic* [tiab] OR post-natal event* [tiab] OR post-natal result* [tiab] OR post-natal problem* [tiab] OR post-natal morbidit* [tiab] OR post-natal sequelae [tiab]

33. antepartum outcome* [tiab] OR antepartum complication* [tiab] OR antepartum consequence* [tiab] OR antepartum characteristic* [tiab] OR antepartum event* [tiab] OR antepartum result* [tiab] OR antepartum problem* [tiab] OR antepartum morbidit* [tiab] OR antepartum sequelae [tiab]

34. ante-partum outcome* [tiab] OR ante-partum complication* [tiab] OR ante-partum consequence* [tiab] OR ante-partum characteristic* [tiab] OR ante-partum event* [tiab] OR ante-partum result* [tiab] OR ante-partum problem* [tiab] OR ante-partum morbidit* [tiab] OR ante-partum sequelae [tiab]

35. intrapartum outcome* [tiab] OR intrapartum complication* [tiab] OR intrapartum consequence* [tiab] OR intrapartum characteristic* [tiab] OR intrapartum event* [tiab] OR intrapartum result* [tiab] OR intrapartum problem* [tiab] OR intrapartum morbidit* [tiab] OR intrapartum sequelae [tiab]

36. intra-partum outcome* [tiab] OR intra-partum complication* [tiab] OR intra-partum consequence* [tiab] OR intra-partum characteristic* [tiab] OR intra-partum event* [tiab] OR intra-partum result* [tiab] OR intra-partum problem* [tiab] OR intra-partum morbidit* [tiab] OR intra-partum sequelae [tiab]

37. peripartum outcome* [tiab] OR peripartum complication* [tiab] OR peripartum consequence* [tiab] OR peripartum characteristic* [tiab] OR peripartum event* [tiab] OR peripartum result* [tiab] OR peripartum problem* [tiab] OR peripartum morbidit* [tiab] OR peripartum sequelae [tiab]

38. peri-partum outcome* [tiab] OR peri-partum complication* [tiab] OR peri-partum consequence* [tiab] OR peri-partum characteristic* [tiab] OR peri-partum event* [tiab] OR peri-partum result* [tiab] OR peri-partum problem* [tiab] OR peri-partum morbidit* [tiab] OR peri-partum sequelae [tiab]

39. postpartum outcome* [tiab] OR postpartum complication* [tiab] OR postpartum consequence* [tiab] OR postpartum characteristic* [tiab] OR postpartum event* [tiab] OR postpartum result* [tiab] OR postpartum problem* [tiab] OR postpartum morbidit* [tiab] OR postpartum sequelae [tiab]

40. post-partum outcome* [tiab] OR post-partum complication* [tiab] OR post-partum consequence* [tiab] OR post-partum characteristic* [tiab] OR post-partum event* [tiab] OR post-partum result* [tiab] OR post-partum problem* [tiab] OR post-partum morbidit* [tiab] OR post-partum sequelae [tiab]

41. Premature Birth [Mesh] OR Fetal Membranes, Premature Rupture [Mesh] OR Obstetric Labor, Premature [Mesh] OR Infant, Extremely Premature [Mesh] OR Infant, Premature [Mesh]

42. prematurity [tiab] OR ìgestational age at birthî [tiab] OR ìgestational age at deliveryî [tiab] OR PTB [tiab] OR PTBs [tiab] OR VPTB [tiab] OR VPTBs [tiab]

43. pre-terms [tiab] OR preterms [tiab] OR pre-term birth* [tiab] OR ìpreterm birth*î [tiab] OR ìpremature birth*î [tiab] OR PTL [tiab] OR PTLs [tiab] OR VPTL [tiab] OR VPTLs [tiab]

44. ìpre-term labor*î [tiab] OR ìpre-term labour*î [tiab] OR ìpre-term obstetric labor*î [tiab] OR ìpre-term obstetric labour*î [tiab] OR ìpreterm labor*î [tiab] OR ìpreterm labour*î [tiab] OR preterm obstetric labor* [tiab] OR ìpreterm obstetric labour*î [tiab] OR ìpremature labor*î [tiab] OR ìpremature labour*î [tiab] OR premature obstetric labor* [tiab] OR ìpremature obstetric labour*î [tiab]

45. PTD [tiab] OR PTDs [tiab] OR VPTD [tiab] OR VPTDs [tiab] OR pre-term deliver* [tiab] OR preterm deliver* [tiab] OR ìpre-term infant*î [tiab] OR ìpreterm infant*î [tiab] OR premature deliver* [tiab] OR ìpremature infant*î [tiab] OR PROM [tiab] OR PPROM [tiab]

46. ìpreterm rupture of membranesî [tiab] OR preterm rupture of fetal membrane* [tiab] OR preterm rupture of foetal membrane* [tiab] OR pre-term rupture of membrane* [tiab] OR pre-term rupture of fetal membrane* [tiab] OR pre-term rupture of foetal membrane* [tiab] OR ìpremature rupture of membranesî [tiab] OR ìpremature rupture of fetal membranesî [tiab] OR ìpremature rupture of foetal membranesî [tiab]

47. Fetal Growth Retardation [Mesh] OR Infant, Low Birth Weight [Mesh] OR Infant, Very Low Birth Weight [Mesh] OR Infant, Extremely Low Birth Weight [Mesh] OR Infant, Small for Gestational Age [Mesh]

48. IUGR [tiab] OR FGR [tiab] OR ìintrauterine growth restrictionî [tiab] OR ìintra-uterine growth restrictionî [tiab] OR ìintrauterine growth restrictedî [tiab] OR ìintra-uterine growth restrictedî [tiab] OR ìintrauterine growth retardationî [tiab] OR ìintra-uterine growth retardationî [tiab]

49. ìfetal growth restrictionî [tiab] OR ìfoetal growth restrictionî [tiab] OR ìfetal growth restrictedî [tiab] OR ìfoetal growth restrictedî [tiab] OR ìfetal growth retardationî [tiab] OR ìfoetal growth retardationî [tiab] OR SGA [tiab] OR SFGA [tiab] OR ìsmall for gestational ageî [tiab] OR ìsmall-for-gestational-ageî [tiab] OR ìsmall-for-gestational ageî [tiab] OR ìsmall for gestationî [tiab] OR ìsmall-for-gestationî [tiab]

50. VSGA [tiab] OR ìvery-small-for-gestational-ageî [tiab] OR ìvery-small-for-gestational ageî [tiab] OR SFD [tiab] OR ìsmall for datesî [tiab] OR ìsmall-for-datesî [tiab] OR ìweight for datesî [tiab] OR ìweight for gestational ageî [tiab] OR ìweight for age at deliveryî [tiab] OR ìweight at deliveryî [tiab]

51. ìbirthweight for datesî [tiab] OR ìbirthweight for gestational ageî [tiab] OR ìbirthweight for age at deliveryî [tiab] OR ìbirth weight for datesî [tiab] OR ìbirth weight for gestational ageî [tiab] OR ìbirth

weight for age at deliveryî [tiab] OR ìbirth-weight for datesî [tiab] OR ìbirth-weight for gestational ageî [tiab] OR ìbirth-weight for age at deliveryî [tiab]

52. LBW [tiab] OR ìlow BWî [tiab] OR ìlow birth weightî [tiab] OR ìlow birth-weightî [tiab] OR ìlow-birth weightî [tiab] OR ìlow-birth-weightî [tiab] OR ìlow birthweightî [tiab] OR ìlow-birthweightî [tiab] OR ìlower BWî [tiab] OR ìlower birth weightî [tiab] OR ìlower birth-weightî [tiab] OR ìlower-birth weightî [tiab] OR ìlower-birth-weightî [tiab] OR ìlower birthweightî [tiab] OR ìlower-birthweightî [tiab]

53. ìreduced birth weightî [tiab] OR ìreduced birthweightî [tiab] OR ìreduced birth-weightî [tiab] OR VLBW [tiab] OR ìvery-low birthweightî [tiab] OR ìvery-low birth weightî [tiab] OR ìvery-low birth-weightî [tiab] OR ìvery-low-birthweightî [tiab] OR ìvery-low-birth-weightî [tiab] OR ELBW [tiab] OR ìextremely-low birthweightî [tiab] OR ìextremely-low birth weightî [tiab] OR ìextremely-low birth-weightî [tiab] OR ìextremely-low-birthweightî [tiab] OR ìextremely-low-birth-weightî [tiab]

54. Stillbirth [Mesh] OR stillbirth* [tiab] OR still birth* [tiab] OR stillborn* [tiab] OR still born* [tiab] OR abortion* [tiab] OR miscarriage* [tiab] OR pregnancy death* [tiab] OR pregnancy loss* [tiab] OR pregnancy demise* [tiab] OR pregnancy mortalit* [tiab] OR gestational death* [tiab] OR gestational loss* [tiab] OR gestational demise* [tiab] OR "gestational mortalit*" [tiab]

55. Fetal Death [Mesh] OR "fetal death*" [tiab] OR "fetal loss*" [tiab] OR fetal demise* [tiab] OR fetal mortalit* [tiab] OR "foetal death*" [tiab] OR "foetal loss*" [tiab] OR foetal demise* [tiab] OR foetal mortalit* [tiab] OR "obstetric death*" [tiab] OR obstetric loss* [tiab] OR obstetric demise* [tiab] OR obstetric mortalit* [tiab] OR obstetrical death* [tiab] OR obstetrical loss* [tiab] OR obstetrical demise* [tiab] OR obstetrical mortalit* [tiab]

56. "labor death*" [tiab] OR labor loss* [tiab] OR labor demise* [tiab] OR labor mortalit* [tiab] OR labour death* [tiab] OR labour loss* [tiab] OR labour demise* [tiab] OR labour mortalit* [tiab] OR birth death* [tiab] OR birth loss* [tiab] OR birth demise* [tiab] OR birth mortalit* [tiab]

57. delivery death* [tiab] OR delivery loss* [tiab] OR delivery demise* [tiab] OR delivery mortalit* [tiab] OR neonate death* [tiab] OR neonate loss* [tiab] OR neonate demise* [tiab] OR neonate mortalit* [tiab] OR "newborn death*" [tiab] OR "newborn loss*" [tiab] OR "newborn demise*" [tiab] OR newborn mortalit* [tiab]

58. new-born death* [tiab] OR new-born loss* [tiab] OR new-born demise* [tiab] OR new-born mortalit* [tiab] OR "new born death*" [tiab] OR "new born loss*" [tiab] OR "new born demise*" [tiab] OR "new born mortalit*" [tiab] OR "infant death*" [tiab] OR infant loss* [tiab] OR infant demise* [tiab] OR infant mortalit* [tiab] OR reproductive death* [tiab] OR "reproductive loss*" [tiab] OR reproductive demise* [tiab] OR reproductive mortalit* [tiab]

59. prelabour death* [tiab] OR prelabour loss* [tiab] OR prelabour demise* [tiab] OR prelabour mortalit* [tiab] OR prelabor death* [tiab] OR prelabor loss* [tiab] OR prelabor demise* [tiab] OR prelabor mortalit* [tiab] OR pre-labour death* [tiab] OR pre-labour loss* [tiab] OR pre-labour demise* [tiab] OR pre-labour mortalit* [tiab] OR pre-labor death* [tiab] OR pre-labor loss* [tiab] OR pre-labor demise* [tiab] OR pre-labor mortalit* [tiab]

60. "intrauterine death*" [tiab] OR intrauterine loss* [tiab] OR intrauterine demise* [tiab] OR intrauterine mortalit* [tiab] OR intra-uterine death* [tiab] OR intra-uterine loss* [tiab] OR intra-uterine demise* [tiab] OR intra-uterine mortalit* [tiab] OR antenatal death* [tiab] OR antenatal loss* [tiab] OR antenatal demise* [tiab] OR antenatal mortalit* [tiab] OR ante-natal death* [tiab] OR ante-natal loss* [tiab] OR ante-natal demise* [tiab] OR ante-natal mortalit* [tiab]

61. prenatal death* [tiab] OR prenatal loss* [tiab] OR prenatal demise* [tiab] OR prenatal mortalit* [tiab] OR pre-natal death* [tiab] OR pre-natal loss* [tiab] OR pre-natal demise* [tiab] OR pre-natal mortalit* [tiab] OR "perinatal death" [tiab] OR "perinatal deaths" [tiab] OR perinatal loss* [tiab] OR perinatal demise* [tiab] OR "perinatal mortality" [tiab] OR "perinatal mortalities" [tiab] OR peri-natal death* [tiab] OR peri-natal loss* [tiab] OR peri-natal demise* [tiab] OR peri-natal mortalit* [tiab]

62. "neonatal death" [tiab] OR "neonatal deaths" [tiab] OR neonatal loss* [tiab] OR neonatal demise* [tiab] OR "neonatal mortality" [tiab] OR "neonatal mortalities" [tiab] OR neo-natal death* [tiab] OR neo-natal loss* [tiab] OR neo-natal demise* [tiab] OR neo-natal mortalit* [tiab] OR postnatal death* [tiab] OR postnatal loss* [tiab] OR postnatal demise* [tiab] OR postnatal mortalit* [tiab] OR post-natal death* [tiab] OR post-natal loss* [tiab] OR post-natal demise* [tiab] OR post-natal mortalit* [tiab]

63. antepartum death* [tiab] OR antepartum loss* [tiab] OR antepartum demise* [tiab] OR antepartum mortalit* [tiab] OR ante-partum death* [tiab] OR ante-partum loss* [tiab] OR ante-partum demise* [tiab] OR ante-partum mortalit* [tiab] OR intrapartum death* [tiab] OR intrapartum loss* [tiab] OR intrapartum demise* [tiab] OR intrapartum mortalit* [tiab] OR intra-partum death* [tiab] OR intra-partum loss* [tiab] OR intra-partum demise* [tiab] OR intra-partum mortalit* [tiab]

64. peripartum death* [tiab] OR peripartum loss* [tiab] OR peripartum demise* [tiab] OR peripartum mortalit* [tiab] OR peri-partum death* [tiab] OR peri-partum loss* [tiab] OR peri-partum demise* [tiab] OR peri-partum mortalit* [tiab] OR postpartum death* [tiab] OR postpartum loss* [tiab] OR postpartum demise* [tiab] OR postpartum mortalit* [tiab] OR post-partum death* [tiab] OR post-partum loss* [tiab] OR post-partum demise* [tiab] OR post-partum mortalit* [tiab]

65. #1 or #2 or #3 or #4 or #5 or #6 or #7 or #8 or #9 or #10 or #11 or #12 or #13 or #14 or #15 or #16 or #17 or #18 or #19 or #20 or #21 or #22 or #23 or #24 or #25 or #26 or #27 or #28 or #29 or #30 or #31 or #32 or #33 or #34 or #35 or #36 or #37 or #38 or #39 or #40 or #41 or #42 or #43 or #44 or #45 or #46 or #47 or #48 or #49 or #50 or #51 or #52 or #53 or #54 or #55 or #56 or #57 or #58 or #59 or #60 or #61 or #62 or #63 or #64

66. "Integrase strand transfer inhibitor" [tiab] OR INSTI [tiab] OR Dolutegravir [tiab] OR DTG [tiab] OR Tivicay [tiab] OR Isentress [tiab] OR Vitekta [tiab]

67. "Formyl peptide receptor 1" [tiab] OR Fuzeon [tiab] OR FPR1 [tiab] OR ENF [tiab] OR Seizentry [tiab] OR Celsentri [tiab]

68. Ziagen [tiab] OR Videx [tiab] OR Emtriva [tiab] OR Coviracil [tiab] OR Zerit [tiab] OR Viread [tiab] OR Vemlidy [tiab] OR Retrovir [tiab] OR Azidothymidine [tiab]

69. "Diarylpyrimidine analogue" [tiab] OR Rescriptor [tiab] OR Sustiva [tiab] OR Intelence [tiab] OR TMC125 [tiab] OR DAPY [tiab] OR Viramune [tiab] OR Edurant [tiab] OR TMC278 [tiab]

70. Reyataz [tiab] OR APV [tiab] OR Agenerase [tiab] OR Prezista [tiab] OR Lexiva [tiab] OR Telzir [tiab] OR Crixivan [tiab] OR LPV/r [tiab] OR ABT-378 [tiab] OR Kaletra [tiab] OR Norvir [tiab] OR Viracept [tiab] OR AG1343 [tiab] OR Invirase [tiab] OR Fortovase [tiab] OR Aptivus [tiab]

71. "Rilpivirine plus dolutegravir" [tiab] OR "Raltegravir plus lamivudine" [tiab] OR "Abacavir plus lamivudine plus dolutegravir" [tiab] OR "Emtricitabine plus tenofovir alafenamide" [tiab] OR "Emtricitabine plus rilpivirine plus tenofovir alafenamide" [tiab] OR "Atazanavir plus cobicistat" [tiab] OR "Darunavir plus cobicistat" [tiab]

72. Juluca [tiab] OR Dutrebis [tiab] OR Stribild [tiab] OR Triumeq [tiab] OR Odefsey [tiab] OR Complera [tiab] OR Descovy [tiab] OR Genvoya [tiab] OR Evotaz [tiab] OR Prezcobix [tiab]

73. "Elvitegravir, Cobicistat, Emtricitabine, Tenofovir Disoproxil Fumarate Drug Combination" [mh] OR "Raltegravir Potassium" [mh] OR Zidovudine [mh] OR Delavirdine [mh] OR "Atazanavir Sulfate" [mh] OR "Emtricitabine, Rilpivirine, Tenofovir Drug Combination" [mh]

74. #66 or #67 or #68 or #69 or #70 or #71 or #72 or #73

75. #65 AND #74

76. 2018/04:2020/04 [dp]

77. #75 AND #76

Supplementary literature search for "pregnancy outcome OR specific perinatal outcomes AND new new antiretroviral therapy"

Database and platform: Pubmed (via https://www.ncbi.nlm.nih.gov/pubmed/)

Latest search date: 30 June 2020

1. Pregnancy Outcome [Mesh] OR Pregnancy Complications, Infectious [Mesh] OR "pregnancy outcome*" [tiab] OR "pregnancy complication*" [tiab] OR pregnancy consequence* [tiab] OR "pregnancy characteristic" [tiab] OR "pregnancy characteristics" [tiab] OR pregnancy event* [tiab] OR pregnancy result* [tiab] OR pregnancy problem* [tiab] OR pregnancy morbidit* [tiab] OR pregnancy sequelae [tiab]

2. gestational outcome* [tiab] OR gestational complication* [tiab] OR gestational consequence* [tiab] OR gestational characteristic* [tiab] OR gestational event* [tiab] OR gestational result* [tiab] OR gestational problem* [tiab] OR gestational morbidit* [tiab] OR gestational sequelae [tiab]

3. "fetal outcome" [tiab] OR "fetal outcomes" [tiab] OR "fetal complication" [tiab] OR "fetal complications" [tiab] OR fetal consequence* [tiab] OR fetal characteristic* [tiab] OR fetal event* [tiab] OR fetal result* [tiab] OR fetal problem* [tiab] OR fetal morbidit* [tiab] OR fetal sequelae [tiab]

4. foetal outcome* [tiab] OR foetal complication* [tiab] OR foetal consequence* [tiab] OR foetal characteristic* [tiab] OR foetal event* [tiab] OR foetal result* [tiab] OR foetal problem* [tiab] OR foetal morbidit* [tiab] OR foetal sequelae [tiab]

5. "obstetric outcome" [tiab] OR "obstetric outcomes" [tiab] OR "obstetric complication" [tiab] OR "obstetric complications" [tiab] OR obstetric consequence* [tiab] OR obstetric characteristic* [tiab] OR obstetric event* [tiab] OR obstetric result* [tiab] OR "obstetric problem" [tiab] OR "obstetric problems" [tiab] OR obstetric morbidit* [tiab] OR obstetric sequelae [tiab]

6. obstetrical outcome* [tiab] OR "obstetrical complication" [tiab] OR "obstetrical complications" [tiab] OR obstetrical consequence* [tiab] OR obstetrical characteristic* [tiab] OR obstetrical event* [tiab] OR obstetrical result* [tiab] OR "obstetrical problem" [tiab] OR "obstetrical problems" [tiab] OR obstetrical morbidit* [tiab] OR obstetrical sequelae [tiab]

7. "labor outcome" [tiab] OR "labor outcomes" [tiab] OR "labor complication" [tiab] OR "labor complications" [tiab] OR "labor consequence" [tiab] OR "labor consequences" [tiab] OR labor characteristic* [tiab] OR labor event* [tiab] OR labor result* [tiab] OR labor problem* [tiab] OR labor morbidit* [tiab] OR labor sequelae [tiab]

8. labour outcome* [tiab] OR labour complication* [tiab] OR "labour consequence" [tiab] OR "labour consequences" [tiab] OR labour characteristic* [tiab] OR labour event* [tiab] OR labour result* [tiab] OR labour problem* [tiab] OR labour morbidit* [tiab] OR labour sequelae [tiab]

9. "birth outcome" [tiab] OR "birth outcomes" [tiab] OR birth complication* [tiab] OR birth consequence* [tiab] OR birth characteristic* [tiab] OR birth event* [tiab] OR birth result* [tiab] OR birth problem* [tiab] OR birth morbidit* [tiab] OR birth sequelae [tiab]

10. delivery outcome* [tiab] OR delivery complication* [tiab] OR delivery consequence* [tiab] OR delivery characteristic* [tiab] OR delivery event* [tiab] OR delivery result* [tiab] OR delivery problem* [tiab] OR delivery morbidit* [tiab] OR delivery sequelae [tiab]

11. neonate outcome* [tiab] OR neonate complication* [tiab] OR neonate consequence* [tiab] OR neonate characteristic* [tiab] OR neonate event* [tiab] OR neonate result* [tiab] OR neonate problem* [tiab] OR neonate morbidit* [tiab] OR neonate sequelae [tiab]

12. newborn outcome* [tiab] OR newborn complication* [tiab] OR newborn consequence* [tiab] OR newborn characteristic* [tiab] OR newborn event* [tiab] OR newborn result* [tiab] OR newborn problem* [tiab] OR newborn morbidit* [tiab] OR newborn sequelae [tiab]

13. new-born outcome* [tiab] OR new-born complication* [tiab] OR new-born consequence* [tiab] OR new-born characteristic* [tiab] OR new-born event* [tiab] OR new-born result* [tiab] OR new-born problem* [tiab] OR new-born morbidit* [tiab] OR new-born sequelae [tiab]

14. new born outcome* [tiab] OR new born complication* [tiab] OR new born consequence* [tiab] OR new born characteristic* [tiab] OR new born event* [tiab] OR new born result* [tiab] OR new born problem* [tiab] OR new born morbidit* [tiab] OR new born sequelae [tiab]

15. infant outcome* [tiab] OR infant complication* [tiab] OR infant consequence* [tiab] OR infant characteristic* [tiab] OR infant event* [tiab] OR infant result* [tiab] OR infant problem* [tiab] OR infant morbidit* [tiab] OR infant sequelae [tiab]

16. reproductive outcome* [tiab] OR reproductive complication* [tiab] OR reproductive consequence* [tiab] OR reproductive characteristic* [tiab] OR reproductive event* [tiab] OR reproductive result* [tiab] OR reproductive problem* [tiab] OR reproductive morbidit* [tiab] OR reproductive sequelae [tiab]

17. prelabour outcome* [tiab] OR prelabour complication* [tiab] OR prelabour consequence* [tiab] OR prelabour characteristic* [tiab] OR prelabour event* [tiab] OR prelabour result* [tiab] OR prelabour problem* [tiab] OR prelabour morbidit* [tiab] OR prelabour sequelae [tiab]

18. prelabor outcome* [tiab] OR prelabor complication* [tiab] OR prelabor consequence* [tiab] OR prelabor characteristic* [tiab] OR prelabor event* [tiab] OR prelabor result* [tiab] OR prelabor problem* [tiab] OR prelabor morbidit* [tiab] OR prelabor sequelae [tiab]

19. pre-labour outcome* [tiab] OR pre-labour complication* [tiab] OR pre-labour consequence* [tiab] OR pre-labour characteristic* [tiab] OR pre-labour event* [tiab] OR pre-labour result* [tiab] OR pre-labour problem* [tiab] OR pre-labour morbidit* [tiab] OR pre-labour sequelae [tiab]

20. pre-labor outcome* [tiab] OR pre-labor complication* [tiab] OR pre-labor consequence* [tiab] OR pre-labor characteristic* [tiab] OR pre-labor event* [tiab] OR pre-labor result* [tiab] OR pre-labor problem* [tiab] OR pre-labor morbidit* [tiab] OR pre-labor sequelae [tiab]

21. intrauterine outcome* [tiab] OR intrauterine complication* [tiab] OR intrauterine consequence* [tiab] OR intrauterine characteristic* [tiab] OR intrauterine event* [tiab] OR intrauterine result* [tiab] OR intrauterine problem* [tiab] OR intrauterine morbidit* [tiab] OR intrauterine sequelae [tiab]

22. intra-uterine outcome* [tiab] OR intra-uterine complication* [tiab] OR intra-uterine consequence* [tiab] OR intra-uterine characteristic* [tiab] OR intra-uterine event* [tiab] OR intra-uterine result* [tiab] OR intra-uterine problem* [tiab] OR intra-uterine morbidit* [tiab] OR intra-uterine sequelae [tiab]

23. antenatal outcome* [tiab] OR antenatal complication* [tiab] OR antenatal consequence* [tiab] OR antenatal characteristic* [tiab] OR antenatal event* [tiab] OR antenatal result* [tiab] OR antenatal problem* [tiab] OR antenatal morbidit* [tiab] OR antenatal sequelae [tiab]

24. ante-natal outcome* [tiab] OR ante-natal complication* [tiab] OR ante-natal consequence* [tiab] OR ante-natal characteristic* [tiab] OR ante-natal event* [tiab] OR ante-natal result* [tiab] OR

ante-natal problem* [tiab] OR ante-natal morbidit* [tiab] OR ante-natal sequelae [tiab]

25. prenatal outcome* [tiab] OR prenatal complication* [tiab] OR prenatal consequence* [tiab] OR prenatal characteristic* [tiab] OR prenatal event* [tiab] OR prenatal result* [tiab] OR prenatal problem* [tiab] OR prenatal morbidit* [tiab] OR prenatal sequelae [tiab]

26. pre-natal outcome* [tiab] OR pre-natal complication* [tiab] OR pre-natal consequence* [tiab] OR pre-natal characteristic* [tiab] OR pre-natal event* [tiab] OR pre-natal result* [tiab] OR pre-natal problem* [tiab] OR pre-natal morbidit* [tiab] OR pre-natal sequelae [tiab]

27. perinatal outcome* [tiab] OR perinatal complication* [tiab] OR perinatal consequence* [tiab] OR perinatal characteristic* [tiab] OR perinatal event* [tiab] OR perinatal result* [tiab] OR perinatal problem* [tiab] OR perinatal morbidit* [tiab] OR perinatal sequelae [tiab]

28. peri-natal outcome* [tiab] OR peri-natal complication* [tiab] OR peri-natal consequence* [tiab] OR peri-natal characteristic* [tiab] OR peri-natal event* [tiab] OR peri-natal result* [tiab] OR peri-natal problem* [tiab] OR peri-natal morbidit* [tiab] OR peri-natal sequelae [tiab]

29. neonatal outcome* [tiab] OR neonatal complication* [tiab] OR neonatal consequence* [tiab] OR neonatal characteristic* [tiab] OR neonatal event* [tiab] OR neonatal result* [tiab] OR neonatal problem* [tiab] OR neonatal morbidit* [tiab] OR neonatal sequelae [tiab]

30. neo-natal outcome* [tiab] OR neo-natal complication* [tiab] OR neo-natal consequence* [tiab] OR neo-natal characteristic* [tiab] OR neo-natal event* [tiab] OR neo-natal result* [tiab] OR neo-natal problem* [tiab] OR neo-natal morbidit* [tiab] OR neo-natal sequelae [tiab]

31. postnatal outcome* [tiab] OR postnatal complication* [tiab] OR postnatal consequence* [tiab] OR postnatal characteristic* [tiab] OR postnatal event* [tiab] OR postnatal result* [tiab] OR postnatal problem* [tiab] OR postnatal morbidit* [tiab] OR postnatal sequelae [tiab]

32. post-natal outcome* [tiab] OR post-natal complication* [tiab] OR post-natal consequence* [tiab] OR post-natal characteristic* [tiab] OR post-natal event* [tiab] OR post-natal result* [tiab] OR post-natal problem* [tiab] OR post-natal morbidit* [tiab] OR post-natal sequelae [tiab]

33. antepartum outcome* [tiab] OR antepartum complication* [tiab] OR antepartum consequence* [tiab] OR antepartum characteristic* [tiab] OR antepartum event* [tiab] OR antepartum result* [tiab] OR antepartum problem* [tiab] OR antepartum morbidit* [tiab] OR antepartum sequelae [tiab]

34. ante-partum outcome* [tiab] OR ante-partum complication* [tiab] OR ante-partum consequence* [tiab] OR ante-partum characteristic* [tiab] OR ante-partum event* [tiab] OR ante-partum result* [tiab] OR ante-partum problem* [tiab] OR ante-partum morbidit* [tiab] OR ante-partum sequelae [tiab]

35. intrapartum outcome* [tiab] OR intrapartum complication* [tiab] OR intrapartum consequence* [tiab] OR intrapartum characteristic* [tiab] OR intrapartum event* [tiab] OR intrapartum result* [tiab] OR intrapartum problem* [tiab] OR intrapartum morbidit* [tiab] OR intrapartum sequelae [tiab]

36. intra-partum outcome* [tiab] OR intra-partum complication* [tiab] OR intra-partum consequence* [tiab] OR intra-partum characteristic* [tiab] OR intra-partum event* [tiab] OR intra-partum result* [tiab] OR intra-partum problem* [tiab] OR intra-partum morbidit* [tiab] OR intra-partum sequelae [tiab]

37. peripartum outcome* [tiab] OR peripartum complication* [tiab] OR peripartum consequence* [tiab] OR peripartum characteristic* [tiab] OR peripartum event* [tiab] OR peripartum result* [tiab] OR peripartum problem* [tiab] OR peripartum morbidit* [tiab] OR peripartum sequelae [tiab]

38. peri-partum outcome* [tiab] OR peri-partum complication* [tiab] OR peri-partum consequence* [tiab] OR peri-partum characteristic* [tiab] OR peri-partum event* [tiab] OR peri-partum result* [tiab] OR peri-partum problem* [tiab] OR peri-partum morbidit* [tiab] OR peri-partum sequelae [tiab]

39. postpartum outcome* [tiab] OR postpartum complication* [tiab] OR postpartum consequence* [tiab] OR postpartum characteristic* [tiab] OR postpartum event* [tiab] OR postpartum result* [tiab] OR postpartum problem* [tiab] OR postpartum morbidit* [tiab] OR postpartum sequelae [tiab]

40. post-partum outcome* [tiab] OR post-partum complication* [tiab] OR post-partum consequence* [tiab] OR post-partum characteristic* [tiab] OR post-partum event* [tiab] OR post-partum result* [tiab] OR post-partum problem* [tiab] OR post-partum morbidit* [tiab] OR post-partum sequelae [tiab]

41. Premature Birth [Mesh] OR Fetal Membranes, Premature Rupture [Mesh] OR Obstetric Labor, Premature [Mesh] OR Infant, Extremely Premature [Mesh] OR Infant, Premature [Mesh]

42. prematurity [tiab] OR ìgestational age at birthî [tiab] OR ìgestational age at deliveryî [tiab] OR PTB [tiab] OR PTBs [tiab] OR VPTB [tiab] OR VPTBs [tiab]

43. pre-terms [tiab] OR preterms [tiab] OR pre-term birth* [tiab] OR ìpreterm birth*î [tiab] OR ìpremature birth*î [tiab] OR PTL [tiab] OR PTLs [tiab] OR VPTL [tiab] OR VPTLs [tiab]

44. ìpre-term labor*î [tiab] OR ìpre-term labour*î [tiab] OR ìpre-term obstetric labor*î [tiab] OR ìpre-term obstetric labour*î [tiab] OR ìpreterm labor*î [tiab] OR ìpreterm labour*î [tiab] OR preterm obstetric labor* [tiab] OR ìpreterm obstetric labour*î [tiab] OR ìpremature labor*î [tiab] OR ìpremature labour*î [tiab] OR premature obstetric labor* [tiab] OR ìpremature obstetric labour*î [tiab]

45. PTD [tiab] OR PTDs [tiab] OR VPTD [tiab] OR VPTDs [tiab] OR pre-term deliver* [tiab] OR preterm deliver* [tiab] OR ìpre-term infant*î [tiab] OR ìpreterm infant*î [tiab] OR premature deliver* [tiab] OR ìpremature infant*î [tiab] OR PROM [tiab] OR PPROM [tiab]

46. ìpreterm rupture of membranesî [tiab] OR preterm rupture of fetal membrane* [tiab] OR preterm rupture of foetal membrane* [tiab] OR pre-term rupture of membrane* [tiab] OR pre-term rupture of fetal membrane* [tiab] OR pre-term rupture of foetal membrane* [tiab] OR ìpremature rupture of membranesî [tiab] OR ìpremature rupture of fetal membranesî [tiab] OR ìpremature rupture of foetal membranesî [tiab]

47. Fetal Growth Retardation [Mesh] OR Infant, Low Birth Weight [Mesh] OR Infant, Very Low Birth Weight [Mesh] OR Infant, Extremely Low Birth Weight [Mesh] OR Infant, Small for Gestational Age [Mesh]

48. IUGR [tiab] OR FGR [tiab] OR ìintrauterine growth restrictionî [tiab] OR ìintra-uterine growth restrictionî [tiab] OR ìintrauterine growth restrictedî [tiab] OR ìintra-uterine growth restrictedî [tiab] OR ìintrauterine growth retardationî [tiab] OR ìintra-uterine growth retardationî [tiab]

49. ìfetal growth restrictionî [tiab] OR ìfoetal growth restrictionî [tiab] OR ìfetal growth restrictedî [tiab] OR ìfoetal growth restrictedî [tiab] OR ìfetal growth retardationî [tiab] OR ìfoetal growth retardationî [tiab] OR SGA [tiab] OR SFGA [tiab] OR ìsmall for gestational ageî [tiab] OR ìsmall-for-gestational-ageî [tiab] OR ìsmall-for-gestational ageî [tiab] OR ìsmall for gestationî [tiab] OR ìsmall-for-gestationî [tiab]

50. VSGA [tiab] OR ìvery-small-for-gestational-ageî [tiab] OR ìvery-small-for-gestational ageî [tiab] OR SFD [tiab] OR ìsmall for datesî [tiab] OR ìsmall-for-datesî [tiab] OR ìweight for datesî [tiab] OR ìweight for gestational ageî [tiab] OR ìweight for age at deliveryî [tiab] OR ìweight at deliveryî [tiab]

51. ìbirthweight for datesî [tiab] OR ìbirthweight for gestational ageî [tiab] OR ìbirthweight for age at deliveryî [tiab] OR ìbirth weight for datesî [tiab] OR ìbirth weight for gestational ageî [tiab] OR ìbirth

weight for age at deliveryî [tiab] OR ìbirth-weight for datesî [tiab] OR ìbirth-weight for gestational ageî [tiab] OR ìbirth-weight for age at deliveryî [tiab]

52. LBW [tiab] OR ìlow BWî [tiab] OR ìlow birth weightî [tiab] OR ìlow birth-weightî [tiab] OR ìlow-birth weightî [tiab] OR ìlow-birth-weightî [tiab] OR ìlow birthweightî [tiab] OR ìlow-birthweightî [tiab] OR ìlower BWî [tiab] OR ìlower birth weightî [tiab] OR ìlower birth-weightî [tiab] OR ìlower-birth weightî [tiab] OR ìlower-birth-weightî [tiab] OR ìlower birthweightî [tiab] OR ìlower-birthweightî [tiab]

53. ìreduced birth weightî [tiab] OR ìreduced birthweightî [tiab] OR ìreduced birth-weightî [tiab] OR VLBW [tiab] OR ìvery-low birthweightî [tiab] OR ìvery-low birth weightî [tiab] OR ìvery-low birth-weightî [tiab] OR ìvery-low-birthweightî [tiab] OR ìvery-low-birth-weightî [tiab] OR ELBW [tiab] OR ìextremely-low birthweightî [tiab] OR ìextremely-low birth weightî [tiab] OR ìextremely-low birth-weightî [tiab] OR ìextremely-low-birthweightî [tiab] OR ìextremely-low-birth-weightî [tiab]

54. Stillbirth [Mesh] OR stillbirth* [tiab] OR still birth* [tiab] OR stillborn* [tiab] OR still born* [tiab] OR abortion* [tiab] OR miscarriage* [tiab] OR pregnancy death* [tiab] OR pregnancy loss* [tiab] OR pregnancy demise* [tiab] OR pregnancy mortalit* [tiab] OR gestational death* [tiab] OR gestational loss* [tiab] OR gestational demise* [tiab] OR "gestational mortalit*" [tiab]

55. Fetal Death [Mesh] OR "fetal death*" [tiab] OR "fetal loss*" [tiab] OR fetal demise* [tiab] OR fetal mortalit* [tiab] OR "foetal death*" [tiab] OR "foetal loss*" [tiab] OR foetal demise* [tiab] OR foetal mortalit* [tiab] OR "obstetric death*" [tiab] OR obstetric loss* [tiab] OR obstetric demise* [tiab] OR obstetric mortalit* [tiab] OR obstetrical death* [tiab] OR obstetrical loss* [tiab] OR obstetrical demise* [tiab] OR obstetrical mortalit* [tiab]

56. "labor death*" [tiab] OR labor loss* [tiab] OR labor demise* [tiab] OR labor mortalit* [tiab] OR labour death* [tiab] OR labour loss* [tiab] OR labour demise* [tiab] OR labour mortalit* [tiab] OR birth death* [tiab] OR birth loss* [tiab] OR birth demise* [tiab] OR birth mortalit* [tiab]

57. delivery death* [tiab] OR delivery loss* [tiab] OR delivery demise* [tiab] OR delivery mortalit* [tiab] OR neonate death* [tiab] OR neonate loss* [tiab] OR neonate demise* [tiab] OR neonate mortalit* [tiab] OR "newborn death*" [tiab] OR "newborn loss*" [tiab] OR "newborn demise*" [tiab] OR newborn mortalit* [tiab]

58. new-born death* [tiab] OR new-born loss* [tiab] OR new-born demise* [tiab] OR new-born mortalit* [tiab] OR "new born death*" [tiab] OR "new born loss*" [tiab] OR "new born demise*" [tiab] OR "new born mortalit*" [tiab] OR "infant death*" [tiab] OR infant loss* [tiab] OR infant demise* [tiab] OR infant mortalit* [tiab] OR reproductive death* [tiab] OR "reproductive loss*" [tiab] OR reproductive demise* [tiab] OR reproductive mortalit* [tiab]

59. prelabour death* [tiab] OR prelabour loss* [tiab] OR prelabour demise* [tiab] OR prelabour mortalit* [tiab] OR prelabor death* [tiab] OR prelabor loss* [tiab] OR prelabor demise* [tiab] OR prelabor mortalit* [tiab] OR pre-labour death* [tiab] OR pre-labour loss* [tiab] OR pre-labour demise* [tiab] OR pre-labour mortalit* [tiab] OR pre-labor death* [tiab] OR pre-labor loss* [tiab] OR pre-labor demise* [tiab] OR pre-labor mortalit* [tiab]

60. "intrauterine death*" [tiab] OR intrauterine loss* [tiab] OR intrauterine demise* [tiab] OR intrauterine mortalit* [tiab] OR intra-uterine death* [tiab] OR intra-uterine loss* [tiab] OR intra-uterine demise* [tiab] OR intra-uterine mortalit* [tiab] OR antenatal death* [tiab] OR antenatal loss* [tiab] OR antenatal demise* [tiab] OR antenatal mortalit* [tiab] OR ante-natal death* [tiab] OR ante-natal loss* [tiab] OR ante-natal demise* [tiab] OR ante-natal mortalit* [tiab]

61. prenatal death* [tiab] OR prenatal loss* [tiab] OR prenatal demise* [tiab] OR prenatal mortalit* [tiab] OR pre-natal death* [tiab] OR pre-natal loss* [tiab] OR pre-natal demise* [tiab] OR pre-natal mortalit* [tiab] OR "perinatal death" [tiab] OR "perinatal deaths" [tiab] OR perinatal loss* [tiab] OR perinatal demise* [tiab] OR "perinatal mortality" [tiab] OR "perinatal mortalities" [tiab] OR peri-natal death* [tiab] OR peri-natal loss* [tiab] OR peri-natal demise* [tiab] OR peri-natal mortalit* [tiab]

62. "neonatal death" [tiab] OR "neonatal deaths" [tiab] OR neonatal loss* [tiab] OR neonatal demise* [tiab] OR "neonatal mortality" [tiab] OR "neonatal mortalities" [tiab] OR neo-natal death* [tiab] OR neo-natal loss* [tiab] OR neo-natal demise* [tiab] OR neo-natal mortalit* [tiab] OR postnatal death* [tiab] OR postnatal loss* [tiab] OR postnatal demise* [tiab] OR postnatal mortalit* [tiab] OR post-natal death* [tiab] OR post-natal loss* [tiab] OR post-natal demise* [tiab] OR post-natal mortalit* [tiab]

63. antepartum death* [tiab] OR antepartum loss* [tiab] OR antepartum demise* [tiab] OR antepartum mortalit* [tiab] OR ante-partum death* [tiab] OR ante-partum loss* [tiab] OR ante-partum demise* [tiab] OR ante-partum mortalit* [tiab] OR intrapartum death* [tiab] OR intrapartum loss* [tiab] OR intrapartum demise* [tiab] OR intrapartum mortalit* [tiab] OR intra-partum death* [tiab] OR intra-partum loss* [tiab] OR intra-partum demise* [tiab] OR intra-partum mortalit* [tiab]

64. peripartum death* [tiab] OR peripartum loss* [tiab] OR peripartum demise* [tiab] OR peripartum mortalit* [tiab] OR peri-partum death* [tiab] OR peri-partum loss* [tiab] OR peri-partum demise* [tiab] OR peri-partum mortalit* [tiab] OR postpartum death* [tiab] OR postpartum loss* [tiab] OR postpartum demise* [tiab] OR postpartum mortalit* [tiab] OR post-partum death* [tiab] OR post-partum loss* [tiab] OR post-partum demise* [tiab] OR post-partum mortalit* [tiab]

65. #1 or #2 or #3 or #4 or #5 or #6 or #7 or #8 or #9 or #10 or #11 or #12 or #13 or #14 or #15 or #16 or #17 or #18 or #19 or #20 or #21 or #22 or #23 or #24 or #25 or #26 or #27 or #28 or #29 or #30 or #31 or #32 or #33 or #34 or #35 or #36 or #37 or #38 or #39 or #40 or #41 or #42 or #43 or #44 or #45 or #46 or #47 or #48 or #49 or #50 or #51 or #52 or #53 or #54 or #55 or #56 or #57 or #58 or #59 or #60 or #61 or #62 or #63 or #64

66. Cobicistat [tiab] OR Tubost [tiab] OR COBI [tiab] OR Rezolsta [tiab] OR QUAD [tiab] OR Epivir [tiab] OR Temixys [tiab] OR Cimduo [tiab] OR Selzentry [tiab] OR Doravirine [tiab] OR DOR [tiab] OR Pifeltro [tiab] OR Ibalizumab-uiyk [tiab] OR Hu5A8 [tiab] OR IBA [tiab] OR Ibalizumab [tiab] OR "TMB-355" [tiab] OR "TNX-355" [tiab] OR Trogarzo [tiab]

67. Bictegravir [tiab] OR BIC [tiab] OR "Bictegravir, emtricitabine, and tenofovir alafenamide fumarate" [tiab] OR "bictegravir sodium/emtricitabine/tenofovir alafenamide fumarate" [tiab] OR "BIC/FTC/TAF" [tiab] OR Biktarvy [tiab] OR Darunavir [tiab] OR Emtricitabine [tiab] OR "tenofovir alafenamide fumarate" [tiab] OR "darunavir ethanolate, cobicistat, emtricitabine, tenofovir alafenamide fumarate" [tiab] OR "DRV/COBI/FTC/TAF" [tiab]

68. Symtuza [tiab] OR "Dolutegravir and lamivudine" [tiab] OR "dolutegravir sodium/lamivudine" [tiab] OR "DTG/3TC" [tiab] OR Dovato [tiab] OR "Doravirine, lamivudine, and tenofovir disoproxil fumarate" [tiab] OR "doravirine/lamivudine/tenofovir disoproxil fumarate" [tiab] OR "DOR/3TC/TDF" [tiab] OR Delstrigo [tiab] OR "Efavirenz, lamivudine, and tenofovir disoproxil fumarate" [tiab] OR "EFV/3TC/TDF" [tiab]

69. Symfi [tiab] OR "Symfi Lo" [tiab] OR "Elvitegravir, cobicistat, emtricitabine, and tenofovir alafenamide fumarate" [tiab] OR "elvitegravir/cobicistat/emtricitabine/tenofovir alafenamide fumarate" [tiab] OR "EVG/COBI/FTC/TAF" [tiab] OR Genvoya [tiab] OR "TMC-114" [tiab] OR "TMC114" [tiab] OR Dideoxyinosine [tiab] OR Racivir [tiab] OR Heptovir [tiab] OR Hepitec [tiab]

70. Zerut [tiab] OR Estavudina [tiab] OR Sanilvudine [tiab] OR Stavudine [tiab] OR Apropovir [tiab] OR Stocrin [tiab] OR Zrivada [tiab] OR Aluvia [tiab] OR Aluviran [tiab] OR Koletra [tiab] OR Pentafuside [tiab] OR Didanosine [tiab] OR Lamivudine [tiab] OR Enfuvirtide [tiab] OR Maraviroc [tiab]

71. Cobicistat [mh] OR Darunavir [mh] OR Emtricitabine [mh] OR "Elvitegravir, Cobicistat, Emtricitabine, Tenofovir Disoproxil Fumarate Drug Combination" [mh] OR Didanosine [mh] OR Lamivudine [mh] OR Stavudine [mh] OR Enfuvirtide [mh] OR Maraviroc [mh]

72. Doravirine [nm] OR Ibalizumab [nm] OR Bictegravir [nm] OR "Bictegravir, Embitcitabine, Tenofovir Alafenamide" [nm] OR Symtuza [nm]

73. "Efavirenz, Lamivudine, Tenofovir Disoproxil Fumarate drug combination" [nm] OR Genvoya [nm] OR Racivir [nm] OR "Lopinavir-Ritonavir drug combination" [nm]

74. #66 or #67 or #68 or #69 or #70 or #71 or #72 or #73

75. #65 AND #74

76. 1980:2020 [dp]

77. #75 AND #76

Appendix 2
Quality assessment of studies

Appendix 2.1
Adapted Newcastle-Ottawa quality assessment tool

A study can be awarded a maximum of one point (for items indicated with an asterisk) for each numbered criterion within the "Selection" and "Outcome" categories.

Selection (maximum 4 points)

1) Representativeness of the exposed cohort.
    - a) Truly representative of the pregnant population in the community. *
    - b) Somewhat representative of the pregnant population in the community.
    - c) Selected group of users, e.g. nurses, volunteers, teenage mothers.
    - d) No description of the derivation of the cohort.

2) Selection of the comparator cohort.
    - a) The comparator cohort is drawn from the same community as the exposed cohort. *
    - b) The comparator cohort is drawn from a different source than the exposed cohort.
    - c) No description of the derivation of the comparator cohort.

3) Ascertainment of exposure.
    - a) ART intake monitored as part of study. *
    - b) ART intake confirmed from secure medical records (e.g. hospital records). *
    - c) Structured interview-participant reported ART intake.
    - d) Written self-report.
    - e) No description.

4) Demonstration that outcome of interest was not present at start of study.
    - a) Yes. *
    - b) No.

Comparability (maximum 2 points)

1) Comparability of cohorts on the basis of the analysis. In the analysis:
    - a) Study controls for BMI, smoking, parity, and maternal age. *
    - b) Study controls for one or more additional factors: e.g. prior history of adverse pregnancy outcome, maternal hypertension, anaemia, illicit drug or alcohol use in pregnancy. *
    - c) Confounding factors not controlled for.

Outcome (maximum 3 points)

1) Ascertainment of outcome.
    - a) Outcome was confirmed following clinical observation of outcome by clinician, midwife or trained birth attendant. *
    - b) Medical records. *
    - c) Self-report.
    - d) No description.

2) Method used to assess gestational age.
    - a) Gestational age was determined according to early ultrasound (<14 weeks). *
    - b) Gestational age was determined by: late ultrasound (≥14 weeks' gestation) or last normal menstrual period or neonatal assessment, e.g. Ballard score, or a combination of these methods.
    - c) No description.

3) Follow up of cohorts

a) Complete follow up - all subjects accounted for. *

b) Subjects lost to follow up unlikely to introduce bias, i.e. < 20 % lost to follow up. *

c) Follow up rate < 80% (lost to follow-up > 20%).

d) No description.

Appendix 2.2
Classification of studies according to quality assessment

Good Quality	9 points – all requirements met
Average Quality	3 points in “Selection” and 3 points in “Outcome” sections
	≥2 points in the “Selection” and “Outcome” sections, as well as ≥1 point in the “Comparability” section.
Poor Quality	< 2 points in the “Selection” and/or “Outcome” sections.
	2 points in the “Selection” and “Outcome” sections, but no points in the “Comparability” section.

Appendix 2.3
Quality assessment of studies included in the systematic review and meta-analysis

Study	Representativeness of the exposed cohort	Selection of comparator cohort	Ascertainment of exposure	Demonstration that outcome of interest was not present at start of study	Comparability of cohorts on the basis of analysis	Ascertainment of outcome	Method used to assess gestational age	Follow up of cohorts	Total quality assessment
**Adam (2016)**[19]	Truly representative*	Same community as exposed cohort*	ART intake monitored as part of study*	No	Study controls for one or more additional confounding factors*	Clinical observation*	No description	Complete follow up*	Average
**Ai-Jie (2013)**[20]	Somewhat representative	Same community as exposed cohort*	Medical records*	No	Confounding factors not controlled for	Medical records*	No description	Complete follow up*	Poor
**Albert (2020)**[21]	Truly representative*	Same community as exposed cohort*	Medical records*	No	Study controls for one or more additional confounding factors*	Medical records*	Ultrasound in first and/or second trimester	Complete follow up*	Average
**Azria (2009)**[22]	Truly representative*	Same community as exposed cohort*	ART intake monitored as part of study*	No	Study controls for one or more additional confounding factors*	Medical records*	First day of LNMP, corrected if needed by routine first trimester ultrasound*	Complete follow up*	Average
**Bailey (2013)**[23]	Truly representative*	Same community as exposed cohort*	Medical records*	No	Confounding factors not controlled for	Medical records*	LNMP and ultrasound (unspecified)	<20% lost to follow up*	Poor
**Balogun (2018)**[24]	Somewhat representative	Same community as exposed cohort*	ART intake monitored as part of study*	Yes*	Study controls for one or more additional confounding factors*	Clinical observation*	LNMP confirmed by ultrasound (unspecified)	Complete follow up*	Average
**Bengtson (2020)**[25]	Truly representative*	Same community as exposed cohort*	ART intake monitored as part of study*	Yes*	Confounding factors not controlled for	Clinical observation*	Ultrasound (unspecified), LNMP, or symphysis-fundal height	Complete follow up*	Poor

**Boer (2006)**[26]	Truly representative*	Different source than the exposed cohort	No description	No	Study controls for one or more additional confounding factors*	No description	LMNP confirmed by first trimester ultrasound*	Complete follow up*	Poor
**Boyajian (2012)**[27]	Truly representative*	Same community as exposed cohort*	Medical records*	No	Study controls for BMI, smoking, parity, and maternal age*, and one or more additional confounding factors*	Medical records*	No description	Complete follow up*	Average
**Carceller (2009)**[28]	Truly representative*	Same community as exposed cohort*	Medical records*	No	Confounding factors not controlled for	Medical records*	No description	<20% lost to follow up*	Poor
**Chagomerana (2017)**[29]	Truly representative*	Same community as exposed cohort*	Medical records*	No	Study controls for one or more additional confounding factors*	Medical records*	LNMP	Complete follow up*	Average
**Chen (2012)**[30]	Truly representative*	Same community as exposed cohort*	Medical records*	No	Study controls for one or more additional confounding factors*	Medical records*	LNMP, symphysis-fundal height, or ultrasound (unspecified)	<20% lost to follow up*	Average
**Chibwesha (2016)**[31]	Truly representative*	Same community as exposed cohort*	Medical records*	No	Confounding factors not controlled for	Medical records*	LNMP and symphysis-fundal height	<20% lost to follow up*	Poor
**Cooper (2002)**[32]	Truly representative*	Same community as exposed cohort*	ART intake monitored as part of study*	Yes*	Study controls for one or more additional confounding factors*	Clinical observation*	LNMP, ultrasound (unspecified), symphysis-fundal height, or neonatal assessment (unspecified)	No description	Poor
**Cotter (2006)**[33]	Truly representative*	Same community as exposed cohort*	ART intake monitored as part of study*	Yes*	Study controls for one or more additional confounding factors*	No description	LNMP and/or ultrasound (unspecified)	Complete follow up*	Poor
**Dadabhai (2019)**[34]	Somewhat representative	Same community as exposed cohort*	Medical records*	Yes*	Study controls for one or more additional confounding factors*	Clinical observation*	Ballard score and LNMP	Complete follow up*	Average

**De Souza (2000)**[35]	Somewhat representative	Same community as exposed cohort*	Medical records*	No	Study controls for one or more additional confounding factors*	Medical records*	No description	<20% lost to follow up*	Average
**Djeha (2019)**[36]	Truly representative*	Same community as exposed cohort*	Medical records*	Yes*	Confounding factors not controlled for	Medical records*	First trimester ultrasound or LNMP*	Complete follow up*	Average
**Duryea (2015)**[37]	Truly representative*	Same community as exposed cohort*	Medical records*	No	Study controls for one or more additional confounding factors*	Medical records*	No description	Complete follow up*	Average
**ECS (2003)**[38]	Truly representative*	Same community as exposed cohort*	ART intake monitored as part of study*	Yes*	Study controls for one or more additional confounding factors*	Clinical observation*	LNMP or ultrasound (unspecified)	<20% lost to follow up*	Average
**Gagnon (2016)**[39]	Truly representative*	Same community as exposed cohort*	Medical records*	No	Study controls for one or more additional confounding factors*	Medical records*	First trimester ultrasound or conception date by assisted reproduction if available*	Complete follow up*	Average
**Garcia-Otero (2019)**[40]	Truly representative*	Same community as exposed cohort*	Medical records*	Yes*	Study controls for one or more additional confounding factors*	Clinical observation*	No description	Complete follow up*	Average
**Gibango (2018)**[41]	Truly representative*	Same community as exposed cohort*	Medical records*	Yes*	Confounding factors not controlled for	Medical records*	Ballard score	<20% lost to follow up*	Poor
**Goetghebuer (2019)**[42]	Truly representative*	Same community as exposed cohort*	Medical records*	Yes*	Study controls for one or more additional confounding factors*	Medical records*	Ballard score	Complete follow up*	Average
**Gonzales (2017)**[43]	Truly representative*	Same community as exposed cohort*	Self-reported	Yes*	Study controls for one or more additional confounding factors*	Clinical observation*	Ballard score, symphysis-fundal height	Complete follow up*	Average
**Habib (2008)**[44]	Truly representative*	Same community as exposed cohort*	Medical records*	No	Study controls for one or more additional confounding factors*	Medical records*	LNMP	Complete follow up*	Average

**Haeri (2009)**[45]	Truly representative*	Same community as exposed cohort*	Medical records*	No	Study controls for one or more additional confounding factors*	Medical records*	LNMP and ultrasound (unspecified)	Complete follow up*	Average
**Hernandez (2017)**[46]	Truly representative*	Same community as the exposed cohort*	No description	Yes*	Study controls for one or more additional confounding factors*	Clinical observation*	No description	Complete follow up*	Average
**Hofer (2016)**[47]	Truly representative*	Same community as exposed cohort*	Medical records*	Yes*	Study controls for one or more additional confounding factors*	Medical records*	No description	Complete follow up*	Average
**Hu (2019)**[48]	Truly representative*	Same community as exposed cohort*	Medical records*	Yes*	Study controls for one or more additional confounding factors*	Clinical observation*	First or second trimester ultrasound, in the absence of ultrasound LNMP used	Complete follow up*	Average
**Joseph (2011)**[49]	Somewhat representative	Same community as exposed cohort*	Medical records*	No	Study controls for one or more additional confounding factors*	Clinical observation*	Not described	Complete follow up*	Average
**Jumare (2019)**[50]	Truly representative*	Same community as exposed cohort*	ART intake monitored as part of study*	Yes*	Study controls for one or more additional confounding factors*	Clinical observation*	LNMP	<20% lost to follow up*	Average
**Kakkar (2015)**[51]	Truly representative*	Same community as exposed cohort*	Medical records*	Yes*	Study controls for one or more additional confounding factors*	Medical records*	Other method: LNMP and ultrasound (unspecified)	<20% lost to follow up*	Average
**Kowalska (2003)**[52]	No description	Same community as exposed cohort*	ART intake monitored as part of study*	Yes*	Study controls for one or more additional confounding factors*	No description	LNMP	Complete follow up*	Poor
**Li (2016)**[10]	Truly representative*	Same community as exposed cohort*	ART intake monitored as part of study*	Yes*	Study controls for one or more additional confounding factors*	No description	LNMP and symphysis-fundal height	Complete follow up*	Poor
**Li (2020)**[53]	Truly representative*	Same community as exposed cohort*	ART intake monitored as part of study*	Yes*	Study controls for one or more additional confounding factors*	Clinical observation*	LNMP or ultrasound (unspecified)	<20% lost to follow up*	Average

**Liff (2020)**[54]	Truly representative*	Same community as exposed cohort*	Medical records*	Yes*	Study controls for one or more additional confounding factors*	Medical records*	Second trimester ultrasound	>20% lost to follow up	Poor
**Lopez (2012)**[55]	Somewhat representative	Same community as exposed cohort*	No description	No	Study controls for one or more additional confounding factors*	No description	Second trimester ultrasound	Complete follow up*	Poor
**Malaba (2017)**[56]	Truly representative*	Same community as exposed cohort*	Self-reported	Yes*	Study controls for one or more additional confounding factors*	Medical records*	LNMP and symphysis-fundal height	<20% lost to follow up*	Average
**Malaba (2018)**[57]	Truly representative*	Same community as exposed cohort*	ART intake monitored as part of study*	Yes*	Study controls for one or more additional confounding factors*	Clinical observation*	LNMP and symphysis-fundal height	Complete follow up*	Average
**Mandelbrot (1998)**[58]	Truly representative*	Same community as exposed cohort*	ART intake monitored as part of study*	No	Confounding factors not controlled for	No description	LNMP, confirmed by first trimester ultrasound*	<20% lost to follow up*	Poor
**Marazzi (2011)**[59]	Somewhat representative	Same community as exposed cohort*	Medical records*	No	Study controls for one or more additional confounding factors*	Medical records*	LNMP and clinical exam (unspecified)	<20% lost to follow up*	Average
**Marti (2007)**[60]	Truly representative*	Same community as exposed cohort*	ART intake monitored as part of study*	Yes*	Confounding factors not controlled for	No description	No description	Complete follow up*	Poor
**Matheson (1995)**[61]	Truly representative*	Same community as exposed cohort*	Medical records*	Yes*	Study controls for one or more additional confounding factors*	Clinical observation*	Ballard score	Complete follow up*	Average
**Mehta (2019)**[62]	Truly representative*	Same community as exposed cohort*	Medical records*	No	Study controls for one or more additional confounding factors*	Clinical observation*	LNMP, ultrasound (unspecified)	Complete follow up*	Average
**Moodley (2016)**[63]	Truly representative*	Same community as exposed cohort*	Medical records*	No	Study controls for one or more additional confounding factors*	Medical records*	LNMP and/or ultrasound (unspecified)	<20% lost to follow up*	Average

**Moseholm (2019)**[64]	Truly representative*	Same community as exposed cohort*	Medical records*	No	Study controls for one or more additional confounding factors*	Medical records *	No description	Complete follow up*	Average
**Olagbuji (2010)**[65]	Somewhat representative	Same community as exposed cohort*	No description	Yes*	Study controls for one or more additional confounding factors*	No description	No description	No description	Poor
**Orloff (2001)**[66]	Truly representative*	Same community as exposed cohort*	Medical records*	No	Confounding factors not controlled for	Medical records*	No description	<20% lost to follow up*	Poor
**Phiri (2015)**[67]	Somewhat representative	Same community as exposed cohort*	Medical records*	No	Study controls for one or more additional confounding factors*	Medical records*	LNMP, ultrasound (unspecified), and clinical assessment (unspecified)	>20% lost to follow up	Poor
**Ramokolo (2017)**[68]	Truly representative*	Same community as exposed cohort*	Self-reported	No	Study controls for one or more additional confounding factors*	Medical records*	LNMP	Complete follow up*	Average
**Rempis (2017)**[69]	Somewhat representative	Same community as exposed cohort*	Self-reported	No	Study controls for one or more additional confounding factors*	Medical records*	No description	<20% lost to follow up*	Poor
**Rudin (2011)**[70]	Truly representative*	Same community as exposed cohort*	ART intake monitored as part of study*	Yes*	Confounding factors not controlled for	No description	No description	No description	Poor
**Santosa (2019)**[71]	Truly representative*	Same community as exposed cohort*	Medical records*	Yes*	Study controls for BMI, smoking, parity, and maternal age*, and one or more additional confounding factors*	Clinical observation*	Ultrasound <14 weeks*	Complete follow up*	Good
**Saums (2019)**[72]	Truly representative*	Same community as exposed cohort*	Medical records*	No	Study controls for one or more additional confounding factors*	Medical records*	No description	Complete follow up*	Average

**Schulte (2007)**[73]	Somewhat representative	Same community as exposed cohort*	Medical records*	No	Study controls for one or more additional confounding factors*	Medical records*	LNMP, ultrasound (unspecified), neonatal assessment (unspecified)	>20% lost to follow up	Poor
**Sebitloane (2017)**[74]	Truly representative*	Same community as exposed cohort*	Medical records*	No	Confounding factors not controlled for	Medical records*	No description	Complete follow up*	Poor
**Short (2014)**[75]	Truly representative*	Same community as exposed cohort*	Medical records*	No	Confounding factors not controlled for	Medical records*	No description	Complete follow up*	Poor
**Silverman (2010)**[76]	No description	Different source than the exposed cohort	No description	No	Study controls for one or more additional confounding factors*	No description	No description	<20% lost to follow up*	Poor
**Simonds (1998)**[77]	Truly representative*	Same community as exposed cohort*	Medical records*	No	Confounding factors not controlled for	Medical records*	Ballard score	Complete follow up*	Poor
**Snijdewind (2018)**[78]	Truly representative*	Same community as exposed cohort*	Medical records*	No	Study controls for one or more additional confounding factors*	Medical records*	Early ultrasound or LNMP	Complete follow up*	Average
**Tiam (2019)**[79]	Truly representative*	Same community as exposed cohort*	Medical records*	Yes*	Confounding factors not controlled for	Structured interviews with nurses and medical records*	LNMP	<20% lost to follow up*	Poor
**Townsend ECS (2010)**[80]	Truly representative*	Same community as exposed cohort*	ART intake monitored as part of study*	Yes*	Study controls for one or more additional confounding factors*	Clinical observation*	LNMP and/or ultrasound (unspecified)	No description	Poor
**Townsend NSHPC (2010)**[80]	Truly representative*	Same community as exposed cohort*	No description	Yes*	Study controls for one or more additional confounding factors*	No description	No description	No description	Poor
**Tuomala (2002)**[81]	Truly representative*	Same community as exposed cohort*	Medical records*	No	Study controls for one or more additional confounding factors*	Medical records*	LNMP and/or ultrasound (unspecified), or neonatal	Complete follow up*	Average

							assessment (unspecified)		
**Van der Merwe (2011)**[82]	Somewhat representative	Different source than the exposed cohort	Medical records*	No	Study controls for one or more additional confounding factors*	Medical records*	LNMP, ultrasound (unspecified), symphysis-fundal height, neonatal assessment (unspecified)	Complete follow up*	Poor
**Von Linstow (2010)**[83]	Truly representative*	Same community as exposed cohort*	Medical records*	No	Confounding factors not controlled for	Medical records*	Late ultrasound at 18-20 weeks	Complete follow up*	Poor
**Watts (2013)**[84]	No description	Same community as exposed cohort*	Medical records*	No	Study controls for one or more additional confounding factors*	Medical records*	Clinical method (unspecified) and ultrasound (unspecified)	<20% lost to follow up*	Average
**Wedderburn (2019)**[85]	Truly representative*	Same community as exposed cohort*	Medical records*	Yes*	Study controls for one or more additional confounding factors*	Clinical observation*	Ultrasound (unspecified), LNMP and symphysis-fundal height	<20% lost to follow up*	Average
**Wilkinson (2015)**[86]	Truly representative*	Same community as exposed cohort*	ART intake monitored as part of study*	Yes*	Study controls for one or more additional confounding factors*	Clinical observation*	LNMP, or symphysis-fundal height	Complete follow up*	Average
**Yu (2012)**[87]	Truly representative*	Same community as exposed cohort*	ART intake monitored as part of study*	No	Study controls for one or more additional confounding factors*	No description	No description	Complete follow up*	Poor
**Zash (2018)**[88]	Truly representative*	Same community as exposed cohort*	Medical records*	No	Study controls for one or more additional confounding factors*	Medical records*	LNMP and/or ultrasound (unspecified), or symphysis-fundal height	Complete follow up*	Average
**Ziske (2013)**[89]	Truly representative*	Same community as exposed cohort*	ART intake monitored as part of study*	Yes*	Study controls for one or more additional confounding factors*	Clinical observation*	No description	>20% lost to follow up	Poor

Abbreviations: ART= antiretroviral therapy, BMI= body mass index, LNMP= last normal menstrual period.

Appendix 2.4
Confounding factors adjusted for in included studies

Study	Methods to assess for confounding factors	**Regression analysis** Confounders corrected for:	**Risk factor analysis** Risk factors not significantly different between groups:	Matching
**Adam (2016)**[19]	Risk factor analysis	-	Maternal age, urban residence, anaemia	-
**Albert (2020)**[21]	Risk factor analysis	-	Maternal age, gravidity, previous PTB, time since HIV diagnosis	-
**Azria (2009)**[22]	Risk factor analysis, matching	-	History of PTB, tobacco smoking during pregnancy, history of illicit drug injection	For each HIV+ infected woman included in the study, two non-referred controls with confirmed HIV- tests, singleton pregnancy, delivered during the same period in the same department and matched by maternal age (+/- 1 year), parity (nulliparous or multiparous) and geographical origin.
**Balogun (2018)**[24]	Risk factor analysis, matching	-	Maternal age, pre-pregnancy BMI, race, parity	Matching between HIV+ and HIV– women was performed on the basis of race, maternal age (±5 years), parity (0, 1, or >1), and body mass index (BMI) (<25 or >25 kg/m2)
**Boer (2006)**[26]	Regression analysis, matching	Maternal age, mode of delivery, parity, cART use in first trimester, maternal CD4 count nadir only	-	Matching HIV- to HIV+: date of expected delivery within 0.5 year, maternal age (±3 years, in most cases ±1 year), parity (nulliparous or multiparous), ethnicity (black, including creole, white or other), singleton or twin
**Boyajian (2012)**[27]	Regression analysis, risk factor analysis, matching	Race, cocaine use, smoking status	Mean maternal age, multiple pregnancy, primiparity, chronic hypertension, renal disease, diabetes mellitus, UTI, history of preeclampsia, maternal obesity, mean BMI, smoker, other drug use during pregnancy	Each HIV+ woman was electronically matched to 3 HIV- women based on year of birth (same year), parity (identical) maternal age (+/- 3 years), and number of foetuses (single or multiple)
**Chagomerana (2017)**[29]	Regression analysis	Maternal age, parity	-	-
**Chen (2012)**[30]	Regression analysis, risk factor analysis	CD4 count in pregnancy, advanced maternal age, nulliparity, maternal hypertension in pregnancy and anaemia	Nationality, education, parity, antenatal care received, syphilis, alcohol, smoking, CD4 count	-

**Cooper (2002)**[32]	Risk factor analysis	-	Maternal age at delivery, duration of membrane rupture, mean maternal CD4 count during pregnancy	-
**Cotter (2006)**[33]	Regression analysis, risk factor analysis	Year of delivery, race, prior PTB, lowest CD4 cell count, CDC stage of disease, weeks receiving ART, pre-pregnancy ART, IDU, alcohol consumption, STD, cigarette smoking, mode of delivery	Maternal age, prior PTB, any sexually transmitted disease during pregnancy	-
**Dadabhai (2019)**[34]	Regression analysis	Maternal age, gravidity, previous pregnancy losses and adverse outcomes, maternal education (years of schooling), BMI at enrolment, anaemia, electricity	-	-
**De Souza (2000)**[35]	Risk factor analysis	-	Race, mode of delivery	-
**Duryea (2015)**[37]	Regression analysis, risk factor analysis	Ethnicity, age, duration of diagnosis, viral load and CD4 count at presentation, delivery	Ethnicity, CD4 count at delivery	-
**ECS (2003)**[38]	Regression analysis, risk factor analysis	IDU, CD4 count, and maternal age	CD4 count	-
**Gagnon (2016)**[39]	Regression analysis, risk factor analysis	Ethnicity, previous PTB, medical history of severe illness	Maternal age, smoking, alcohol, IDU, STD	-
**Garcia-Otero (2019)**[40]	Risk factor analysis	-	Black ethnicity, low socioeconomic status, smoking, HCV infection	-
**Goetghebuer (2019)**[42]	Risk factor analysis	-	Education, literacy, occupation, smoking, alcohol, GBS positive swab	-
**Gonzales (2017)**[43]	Risk factor analysis	-	Maternal weight, maternal height, MUAC, malnutrition, presenting in third trimester	-
**Habib (2008)**[44]	Regression analysis	Year of birth, maternal residence, maternal occupation, paternal tribe	-	-
**Haeri (2009)**[45]	Regression analysis, risk factor analysis, matching	Smoking, cocaine use	Age, race, chronic hypertension, pregestational diabetes	Matched HIV-positive woman on cART with HIV-negative women (1:2): maternal age, race, parity, care location, insurance type, year, and mode of delivery
**Hernandez (2017)**[46]	Risk factor analysis, matching	-	Maternal age, race, HCV infection	Age
**Hofer (2016)**[47]	Risk factor analysis	-	Family income, infant gender, year of birth	-

**Hu (2019)**[48]	Regression analysis, risk factor analysis	Maternal education, ethnicity, household registration, parity, gestational age at first ANC, number of ANC visits, mode of delivery	Maternal age, marital status	-
**Joseph (2011)**[49]	Risk factor analysis	-	Maternal age and parity	-
**Jumare (2019)**[50]	Risk factor analysis	-	Employment, marriage, maternal weight	-
**Kakkar (2015)**[51]	Regression analysis, risk factor analysis	Maternal age, CD4 count, parity, HCV infection, race	CD4 count	-
**Kowalska (2003)**[52]	Risk factor analysis	-	Maternal age, CD4 count, viral load, hard drug use	-
**Li (2016)**[10]	Risk factor analysis	-	Maternal age, year of delivery, diarrhoea, AIDS-defining illness, tuberculosis history	-
**Li (2020)**[53]	Regression analysis, risk factor analysis	Maternal age, residence, ethnicity, education level, occupation, gravidity, parity, severe anaemia, pregnant syphilis, HBV infection	Maternal age, urban/non-urban, ethnicity, parity, syphilis, HBV infection	-
**Liff (2020)**[54]	Risk factor analysis	-	Employment, language spoken at home, alcohol use, malaria, tuberculosis, other chronic disease, history of PTB, history of miscarriage or termination, history of cervical surgery, timing of initiation of ANC	-
**Lopez (2012)**[55]	Regression analysis, risk factor analysis, matching	Previous history of prematurity, nulliparity	Maternal age, black ethnicity, low educational level, smoking, regular cocaine or amphetamines use	Ethnicity (black vs. nonblack), smoking (non-smoking vs. 1 cigarette), maternal age (2 years), educational level (less vs. more than 8 years of education)
**Malaba (2017)**[56]	Regression analysis, risk factor analysis	Maternal age, maternal height, parity, previous PTB, CD4 count, viral load	Socio-economic status, height, CD4 count	-
**Malaba (2018)**[57]	Regression analysis	Maternal age, parity, BMI, previous PTB	-	-
**Marazzi (2011)**[59]	Regression analysis	Baseline viral load, baseline CD4 cell count, baseline haemoglobin, baseline BMI, predelivery days in care, gestational age at the beginning of cART	-	-
**Matheson (1995)**[61]	Risk factor analysis	-	Race, IDU, sex partner, other HIV risk factors	-

**Mehta (2019)**[62]	Risk factor analysis	-	Education, previous adverse pregnancy outcome, pre-existing diabetes, epilepsy status	-
**Moodley (2016)**[63]	Regression analysis, risk factor analysis	Year age group, mode of delivery, HIV status	Maternal age, year, mode of delivery, CD4 count	-
**Moseholm (2019)**[64]	Risk factor analysis, matching	-	Place of birth, BMI, nulliparity	Children were matched by child sex, parity and maternal place of birth to 5 singleton controls born to mothers without HIV
**Olagbuji (2010)**[65]	Risk factor analysis	-	Maternal age (mean and age distribution), marital status	-
**Phiri (2015)**[67]	Regression analysis	Non-ART medication during pregnancy, smoking, alcohol, birth year cohort, HIV-related maternal illness during pregnancy	-	-
**Ramokolo (2017)**[68]	Risk factor analysis	-	Syphilis serology, tuberculosis, maternal age, parity, household food insecurity, infant gender, maternal education	-
**Rempis (2017)**[69]	Risk factor analysis	-	Maternal age, no of persons in household, income, socio-economic status, travel distance to hospital, referral, grand multiparity, hypertension, MIP reported, anaemia, ANC attendance, number of ANC visits	-
**Santosa (2019)**[71]	Regression analysis, risk factor analysis	Maternal age, smoking, alcohol consumption, pre-pregnancy BMI, parity, history of adverse perinatal outcomes	Married/cohabiting, occupation, smoked during pregnancy, alcohol during pregnancy, pre-pregnancy BMI, history of prior adverse perinatal outcomes, gestational age at enrolment	-
**Saums (2019)**[72]	Risk factor analysis	-	Parity, history of diabetes, hypertension, diabetes, obesity, IDU	-
**Schulte (2007)**[73]	Regression analysis	IDU, unknown HIV/AIDS before delivery, symptomatic HIV, no treatment, race, infant gender, gestational age, HIV-status of infant	-	-
**Silverman (2010)**[76]	Risk factor analysis	-	CD4 count, anaemia, multiple birth, febrile illness, hypertension, diabetes, malaria	-

**Snijdewind (2018)**[78]	Risk factor analysis	-	Region of origin, smoking, alcohol, IDU, mode of delivery	-
**Townsend ECS (2010)**[80]	Regression analysis	Race/ethnicity, region of birth, IDU, clinical HIV status, year of delivery, study site	-	-
**Townsend NSHPC (2010)**[80]	Regression analysis	Race, region of birth, IDU, clinical HIV status, year of delivery, study site	-	-
**Tuomala (2002)**[81]	Regression analysis, risk factor analysis	CD4+ cell count, maternal age, race or ethnic group, tobacco use, alcohol use, IDU, prior history of premature delivery, year of delivery	Maternal age, race or ethnic group, prior premature delivery	-
**Van der Merwe (2011)**[82]	Regression analysis, risk factor analysis	CD4 cell count, maternal age, hypertension, infant PCR	Race, smoking, alcohol, WHO stage, hypertension, diabetes, syphilis, gravidity, previous miscarriage, mode of delivery, infant gender	-
**Watts (2013)**[84]	Regression analysis	Black or African American race, annual household income <$20 000/year, cigarette smoking, maternal CD4+ T-cell count of <200 cells/mm^3 at delivery	-	-
**Wedderburn (2019)**[85]	Risk factor analysis	-	Infant gender, income, education, employment, married/cohabiting, smoking, alcohol, depression	-
**Wilkinson (2015)**[86]	Risk factor analysis	-	Maternal age, primiparity, MUAC, triceps skinfold thickness, weight, height, BMI	-
**Yu (2012)**[87]	Risk factor analysis	-	Age, nationality, weight, gravidity, HIV transmission route, parity, mode of delivery, viral load, cotrimoxazole, haemoglobin, CD4 count	-
**Zash (2018)**[88]	Regression analysis	Maternal age, gravidity, education	-	-
**Ziske (2013)**[89]	Risk factor analysis	-	CD4 count at delivery, mode of delivery, infant gender	-

Abbreviations: AIDS= acquired immunodeficiency syndrome, ANC=antenatal clinic, ART= antiretroviral therapy, BMI= body mass index, cART= combination ART, CDC= Centers for Disease Control and Prevention, GBS= group B streptococcus, HBV= hepatitis B virus, HCV= hepatitis C virus, HIV= human immunodeficiency virus, IDU= illicit drug use, MIP=malaria in pregnancy, MUAC=mid-upper arm circumference, PTB=preterm birth, STD= sexually transmitted disease, UTI= urinary tract infection.

Appendix 3
Random-effects meta-analyses of risk of perinatal outcomes associated with pregnancies in women living with HIV receiving ART.

Forest plots showing random-effects meta-analyses of risk of perinatal outcomes associated with pregnancies in women living with HIV receiving ART vs women living with HIV without ART, and in women living with HIV receiving ART vs HIV-negative women. Relative risk (RR) and 95% confidence intervals (CIs).

Appendix 3.1
Women living with HIV receiving ART vs women living with HIV without ART

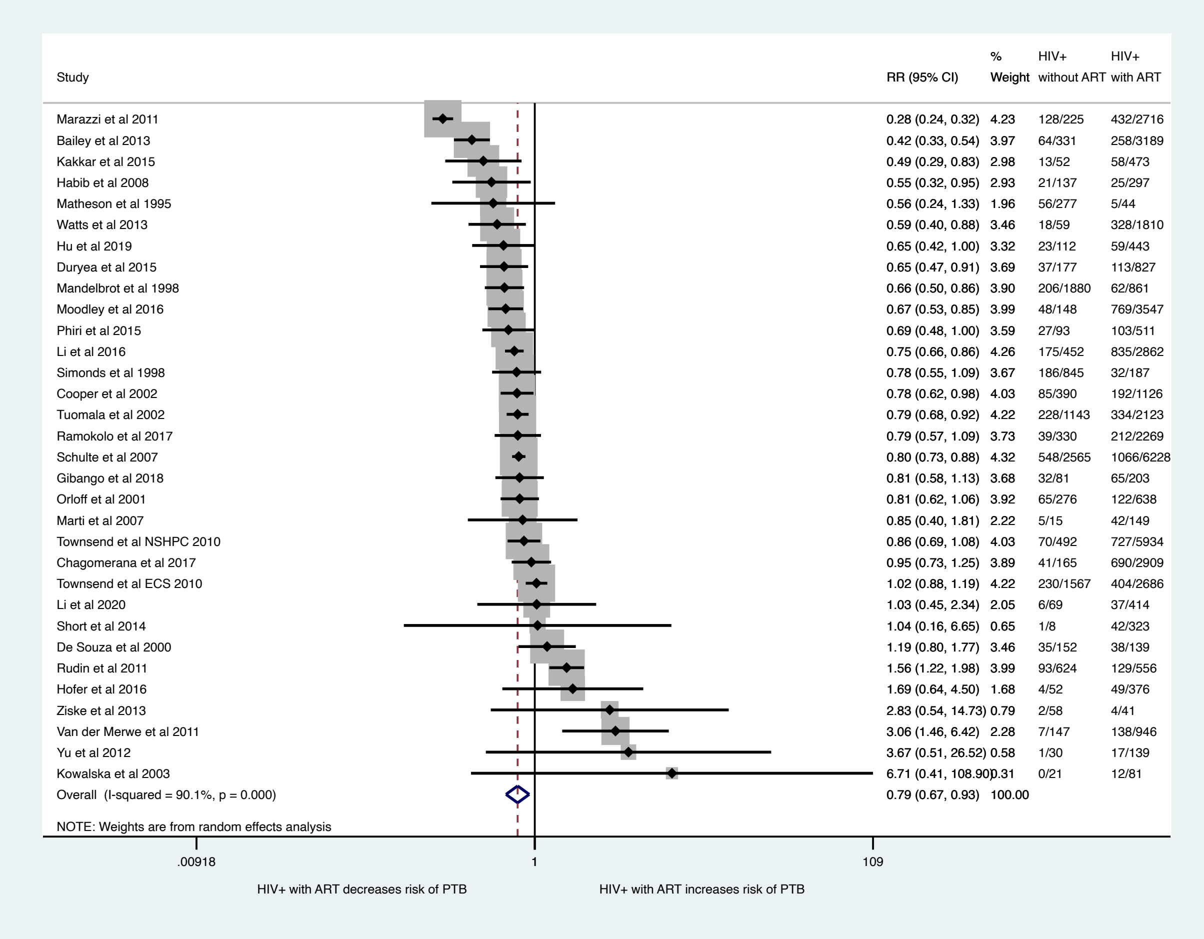

Figure 3.1.1 Preterm birth (PTB) in women living with HIV receiving ART vs women living with HIV without ART.

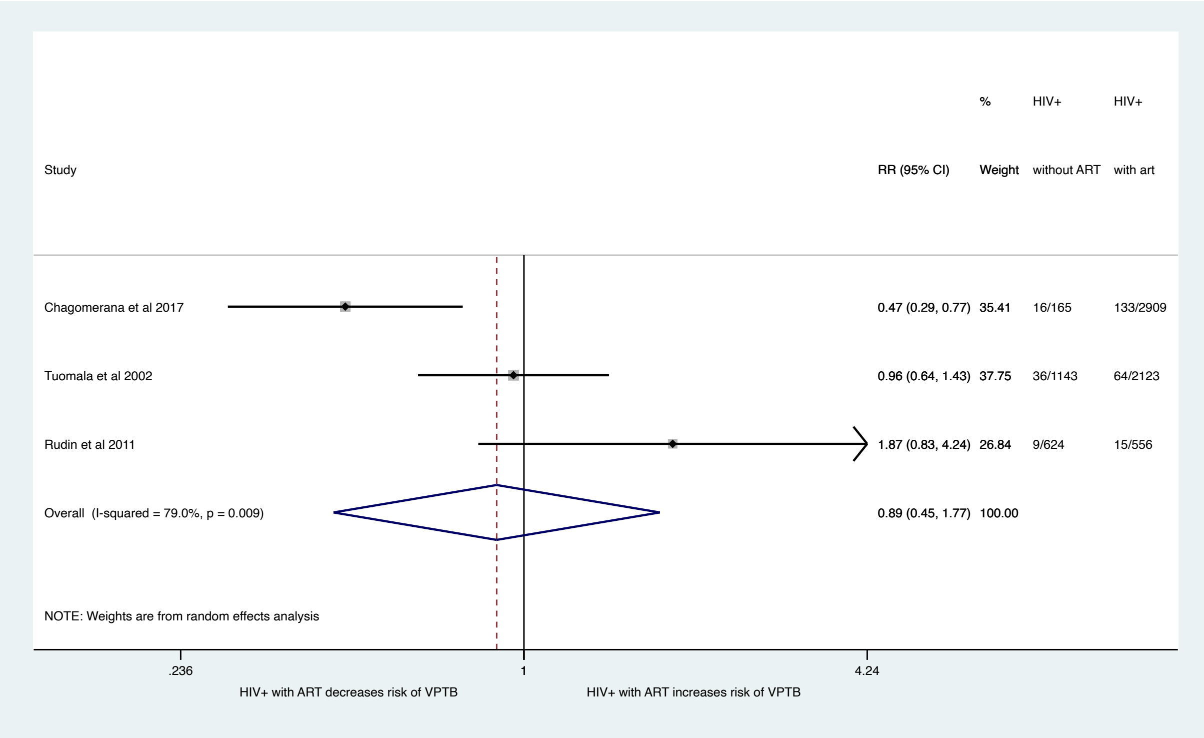

Figure 3.1.2 Very preterm birth (VPTB) in women living with HIV receiving ART vs women living with HIV without ART.

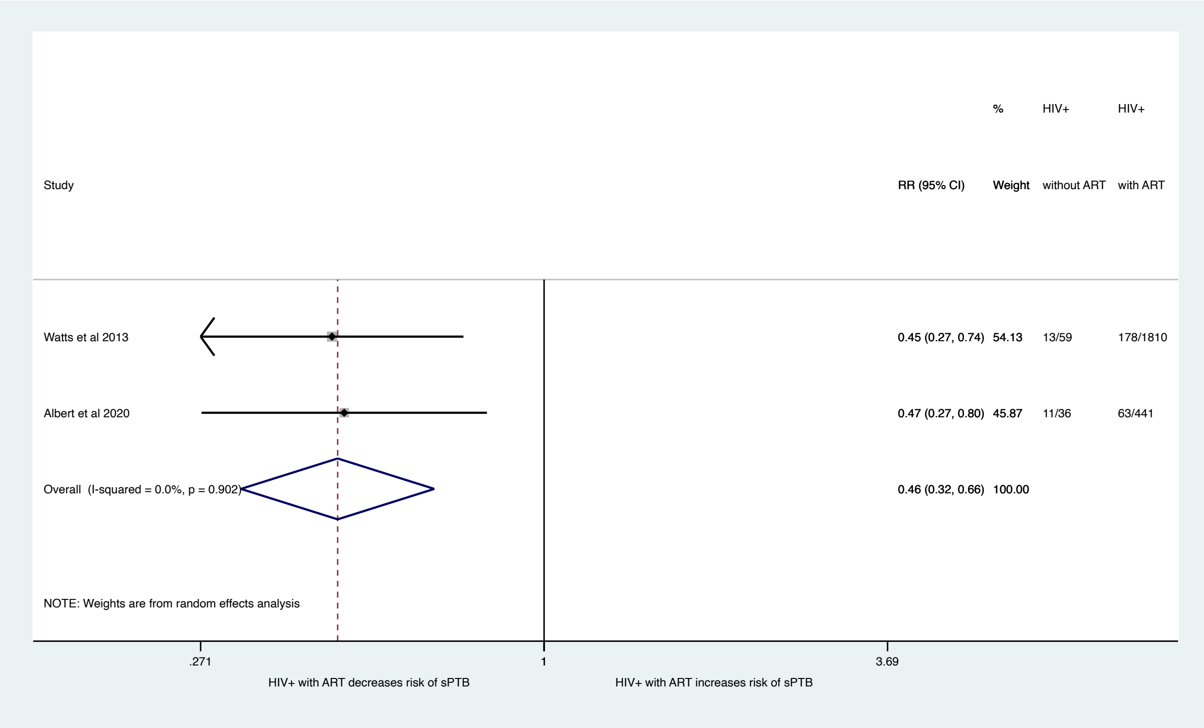

Figure 3.1.3 Spontaneous preterm birth (sPTB) in women living with HIV receiving ART vs women living with HIV without ART.

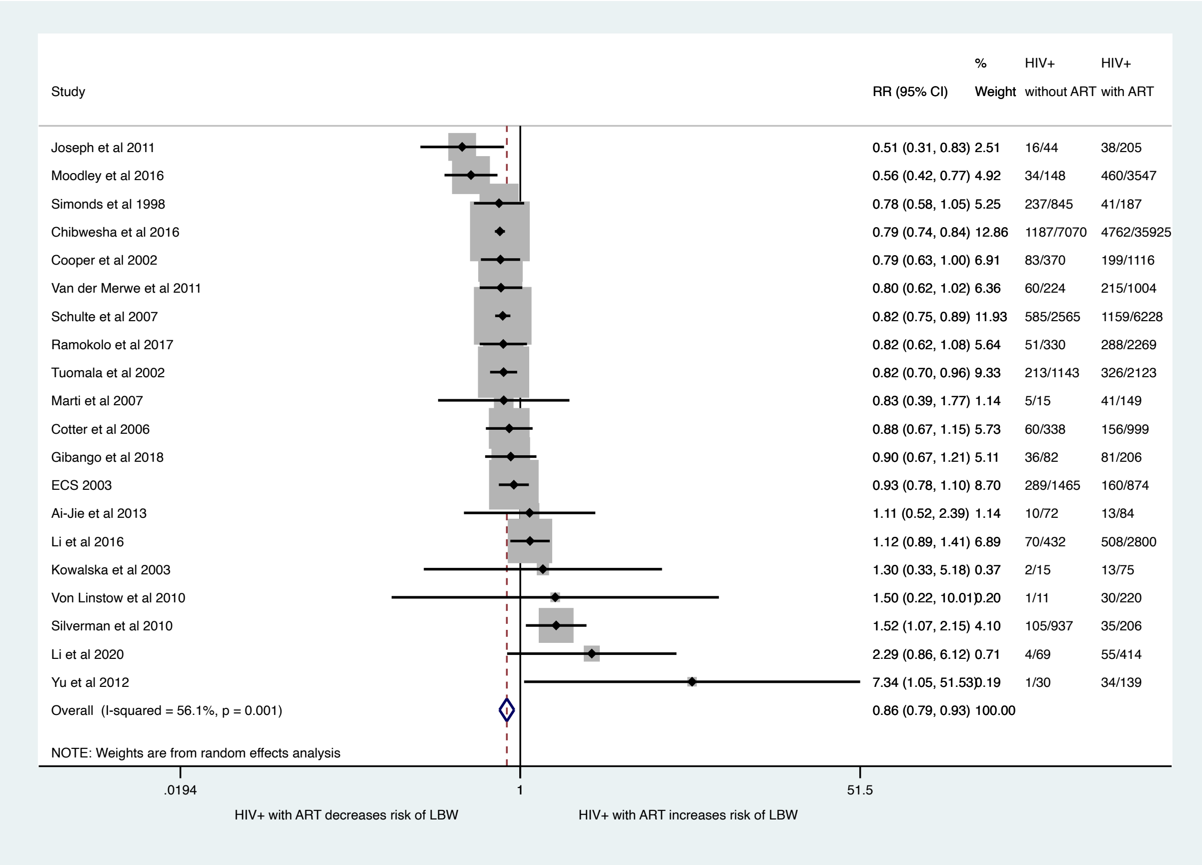

Figure 3.1.4 Low birthweight (LBW) in women living with HIV receiving ART vs women living with HIV without ART.

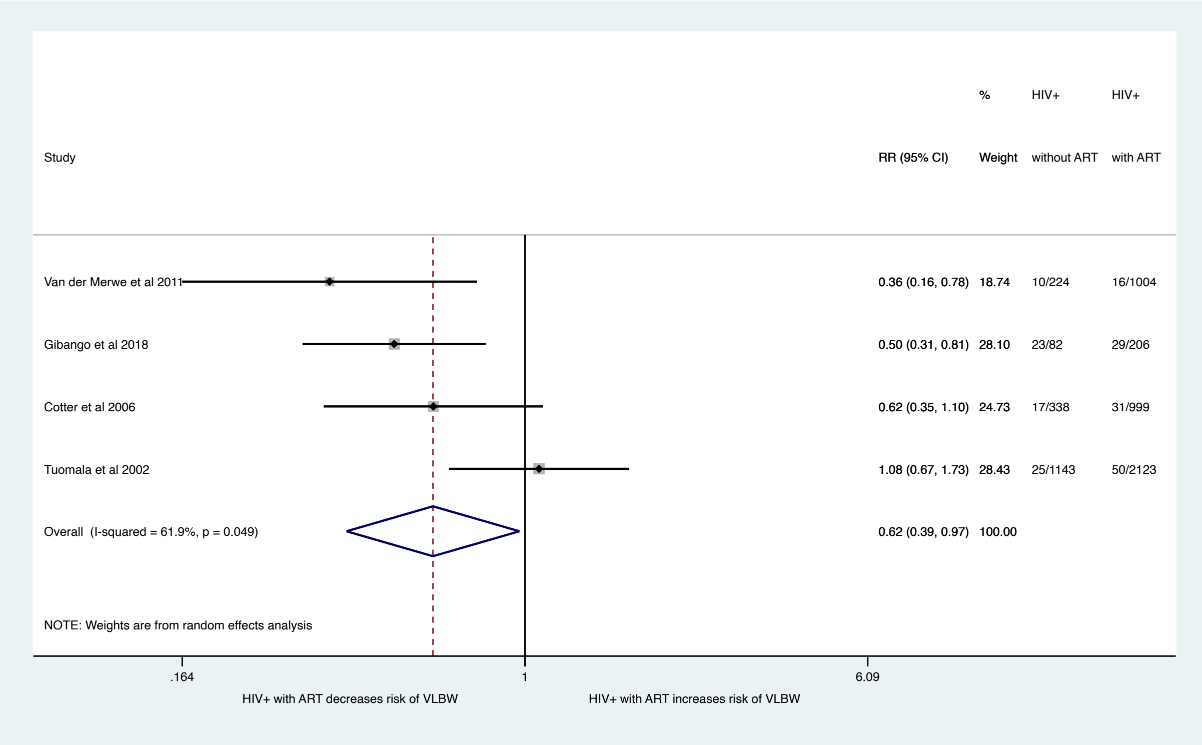

Figure 3.1.5 Very low birthweight (VLBW) in women living with HIV receiving ART vs women living with HIV without ART.

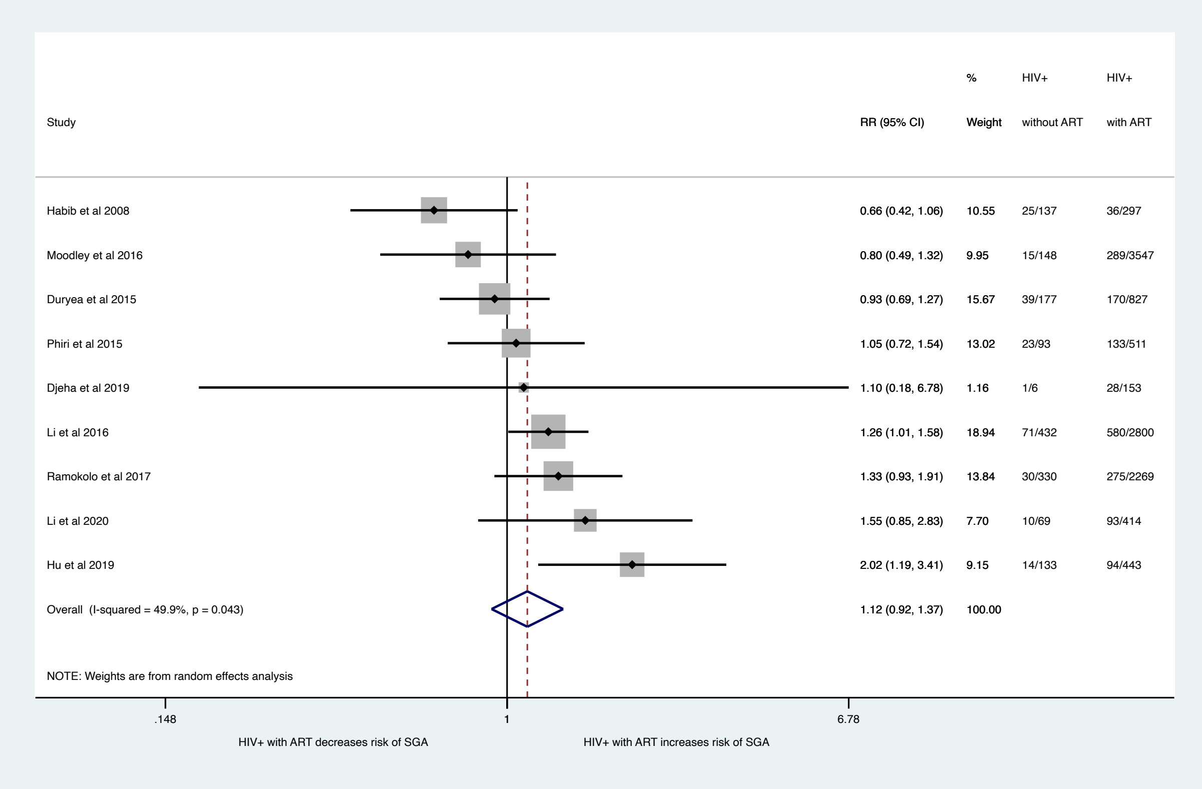

Figure 3.1.6 Small for gestational age (SGA) in women living with HIV receiving ART vs women living with HIV without ART.

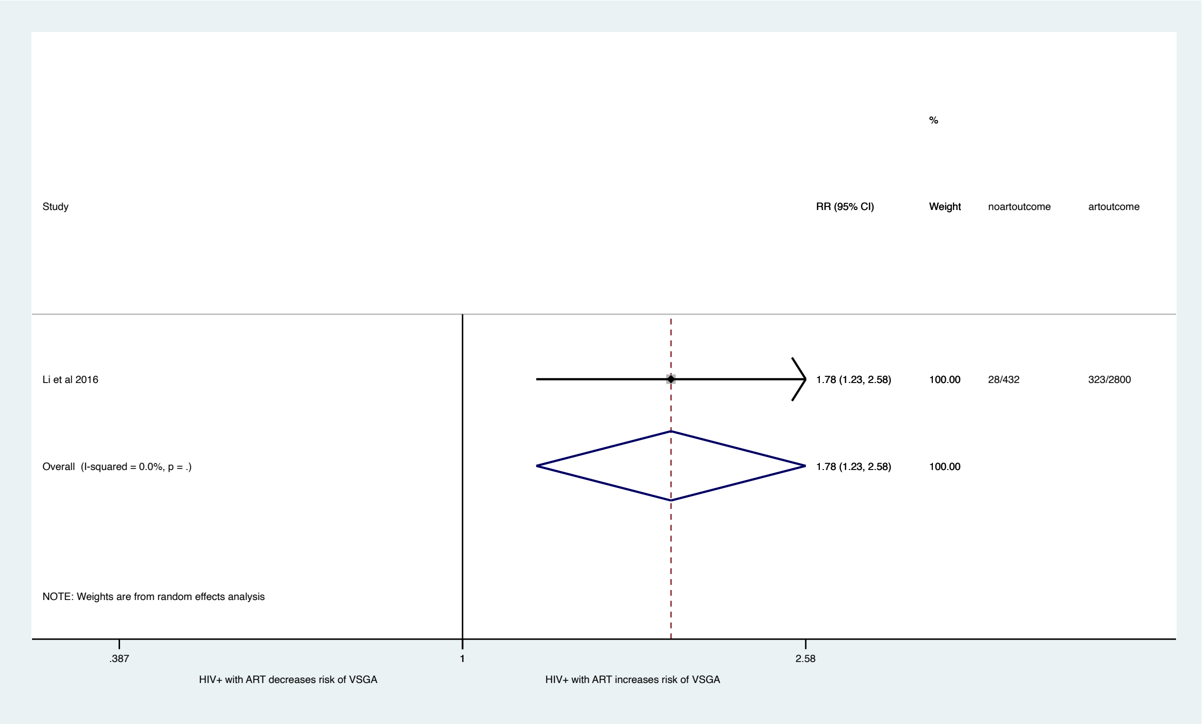

Figure 3.1.7 Very small for gestational age (VSGA) in women living with HIV receiving ART vs women living with HIV without ART.

Appendix 3.2
Women living with HIV receiving ART vs HIV-negative women

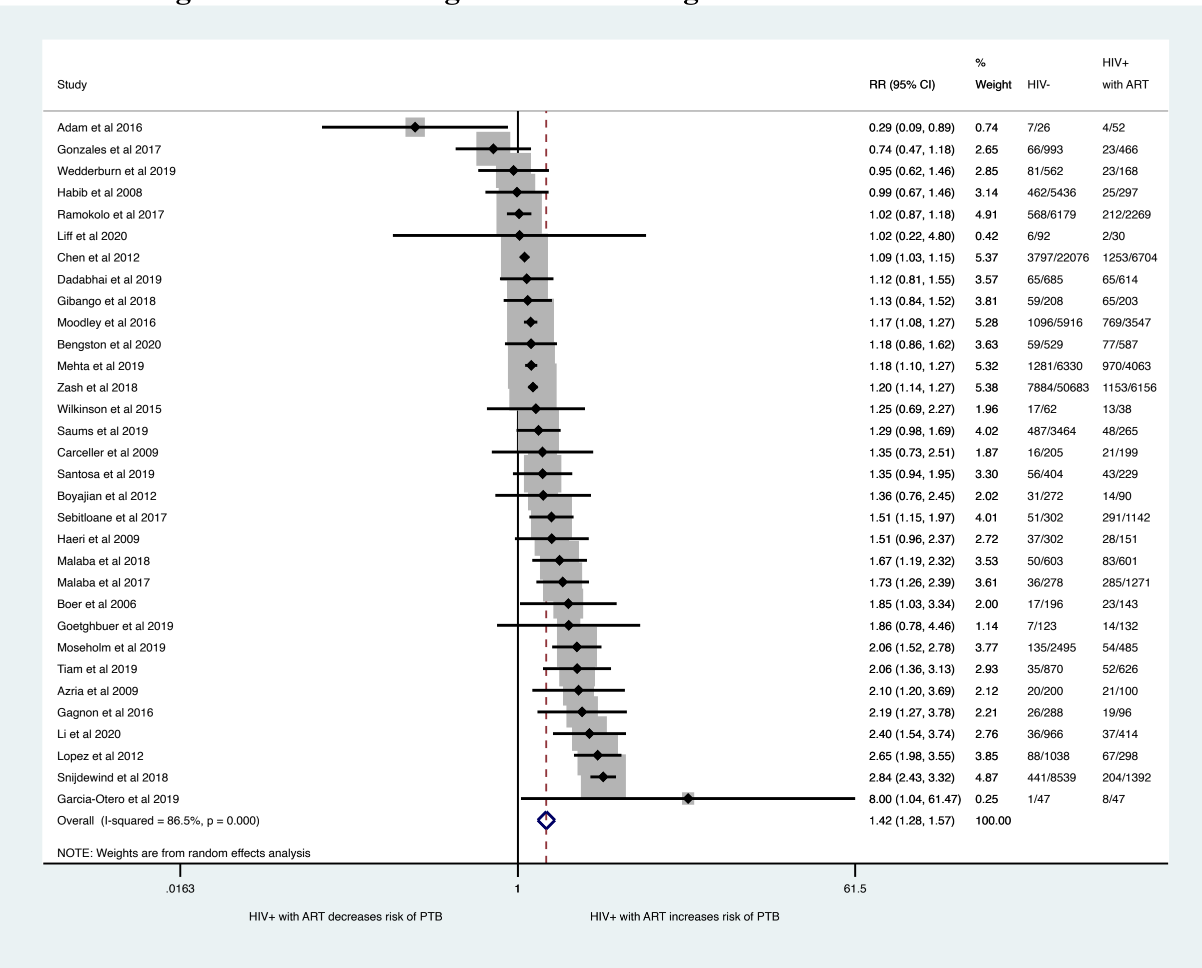

Figure 3.2.1 Preterm birth (PTB) in women living with HIV receiving ART vs HIV-negative women.

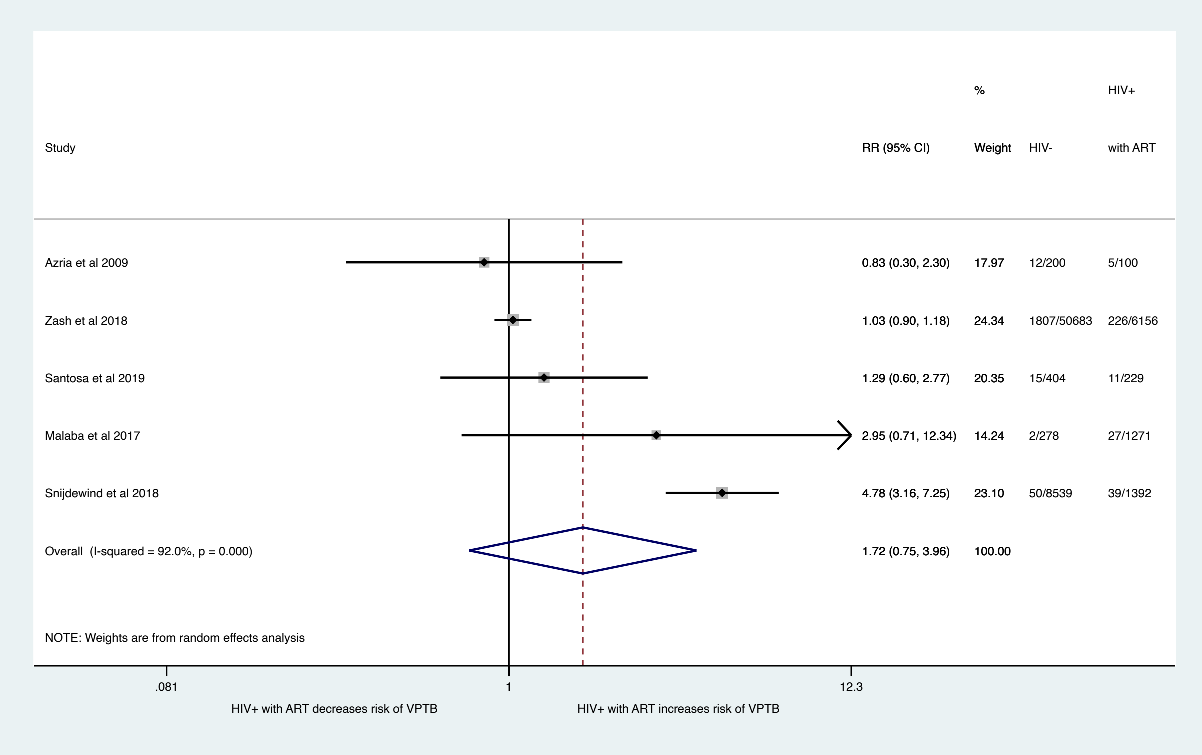

Figure 3.2.2 Very preterm birth (VPTB) in *w*omen living with HIV receiving ART vs HIV-negative women.

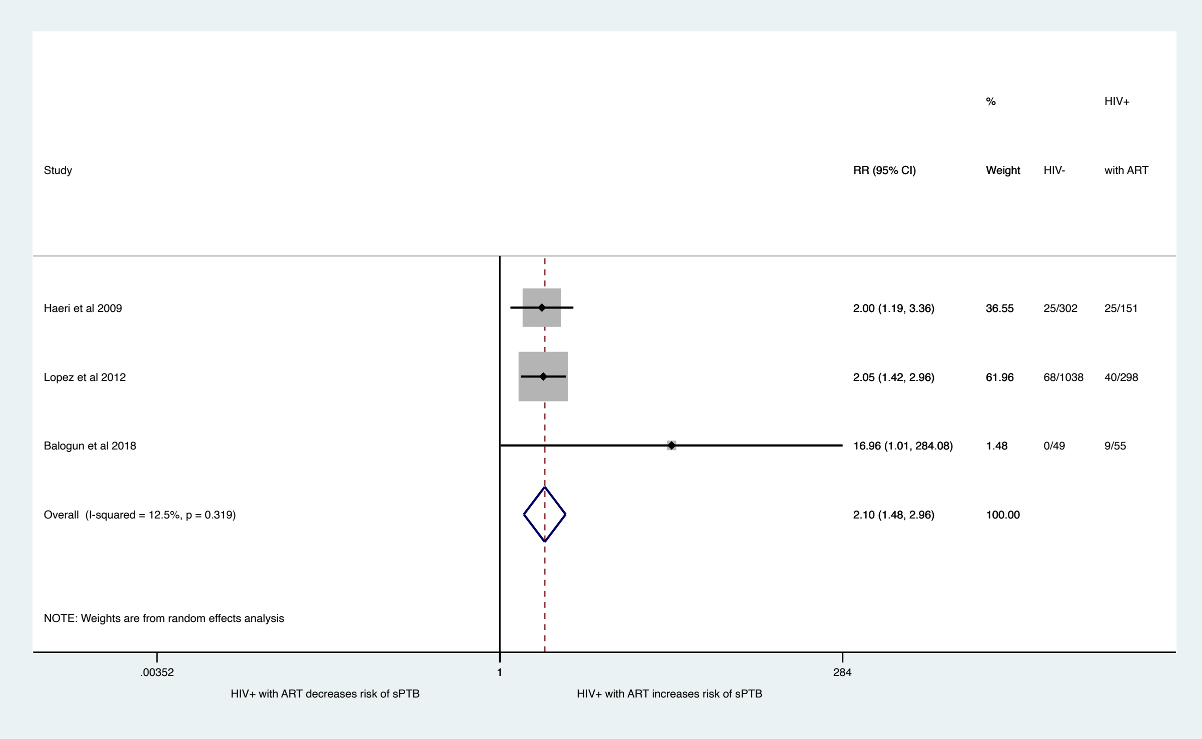

Figure 3.2.3 Spontaneous preterm birth (sPTB) in *w*omen living with HIV receiving ART vs HIV-negative women.

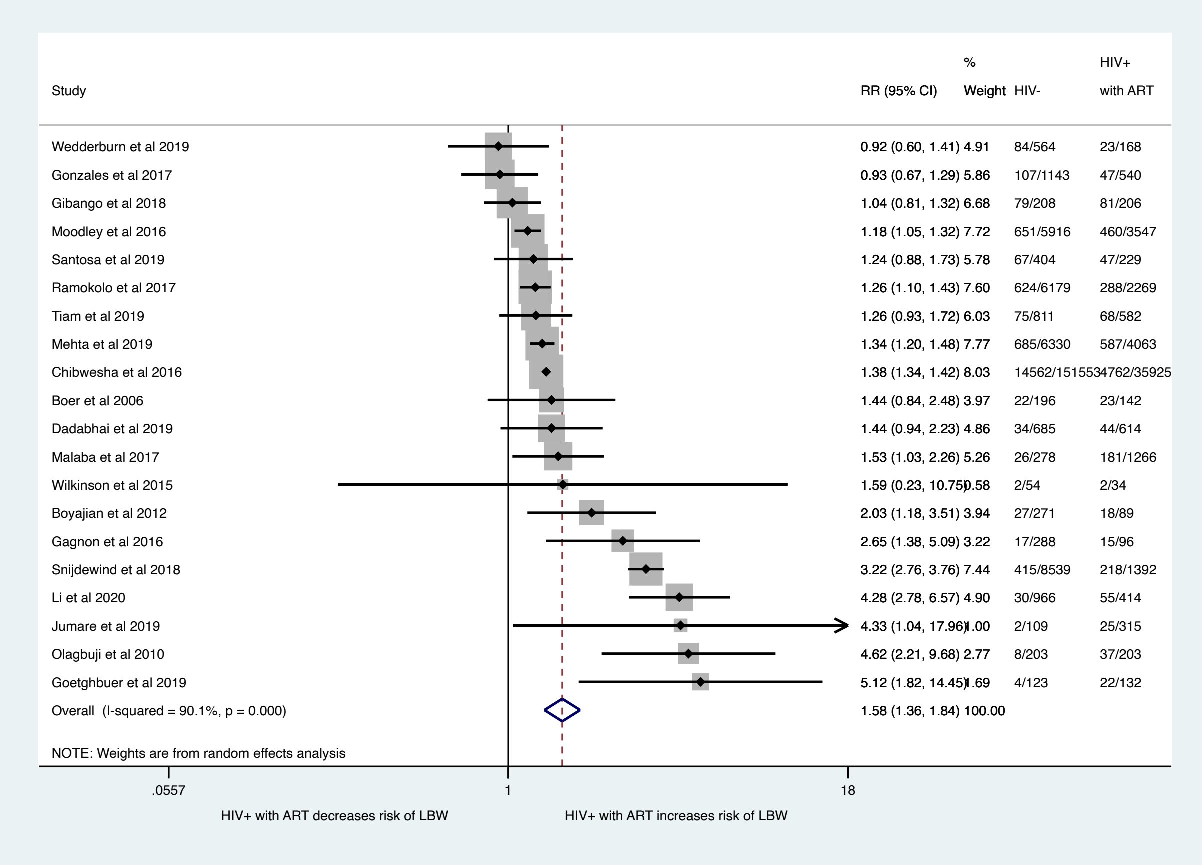

Figure 3.2.4 Low birthweight (LBW) in women living with HIV receiving ART vs HIV-negative women.

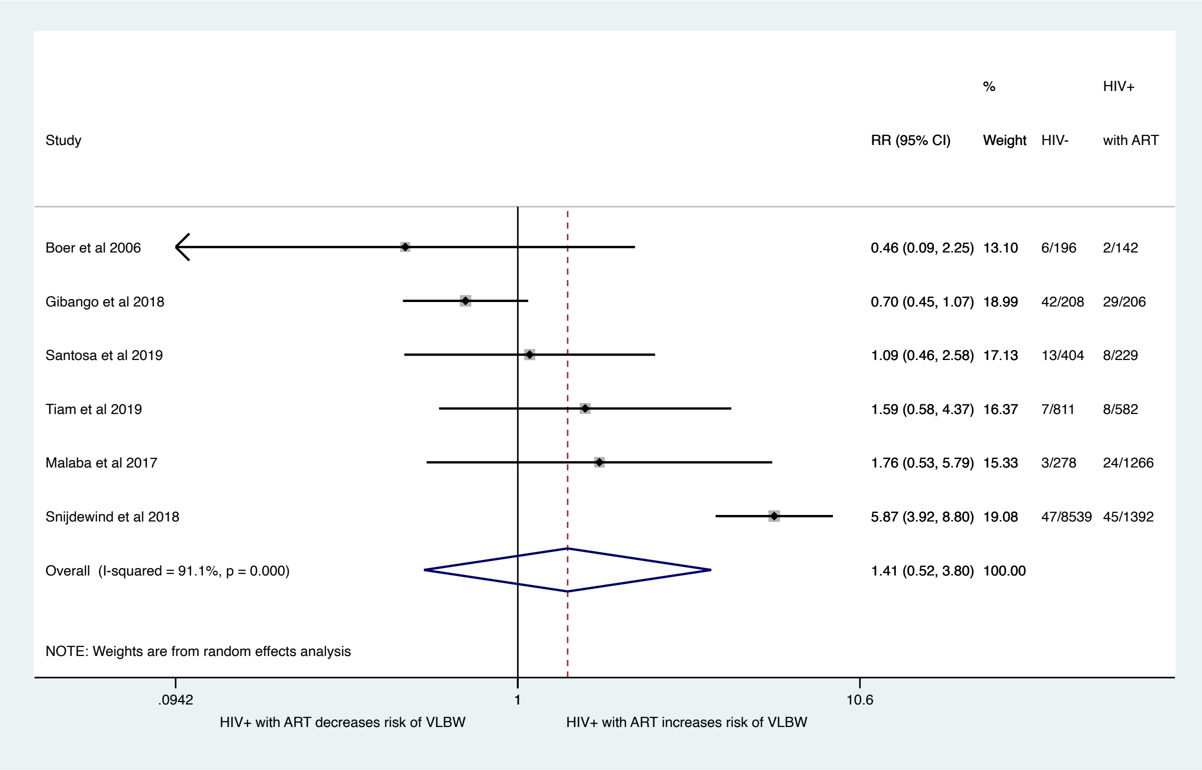

Figure 3.2.5 Very low birthweight (VLBW) in *w*omen living with HIV receiving ART vs HIV-negative women.

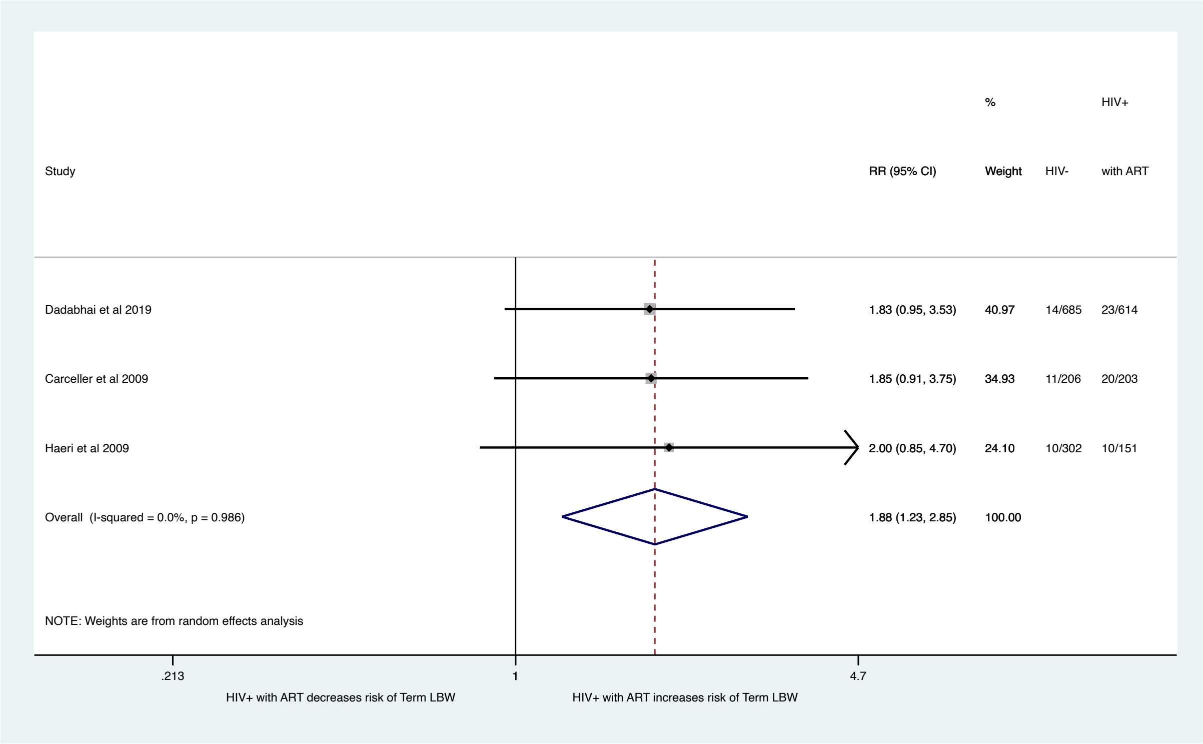

Figure 3.2.6 Term low birthweight (term LBW) in *w*omen living with HIV receiving ART vs HIV-negative women.

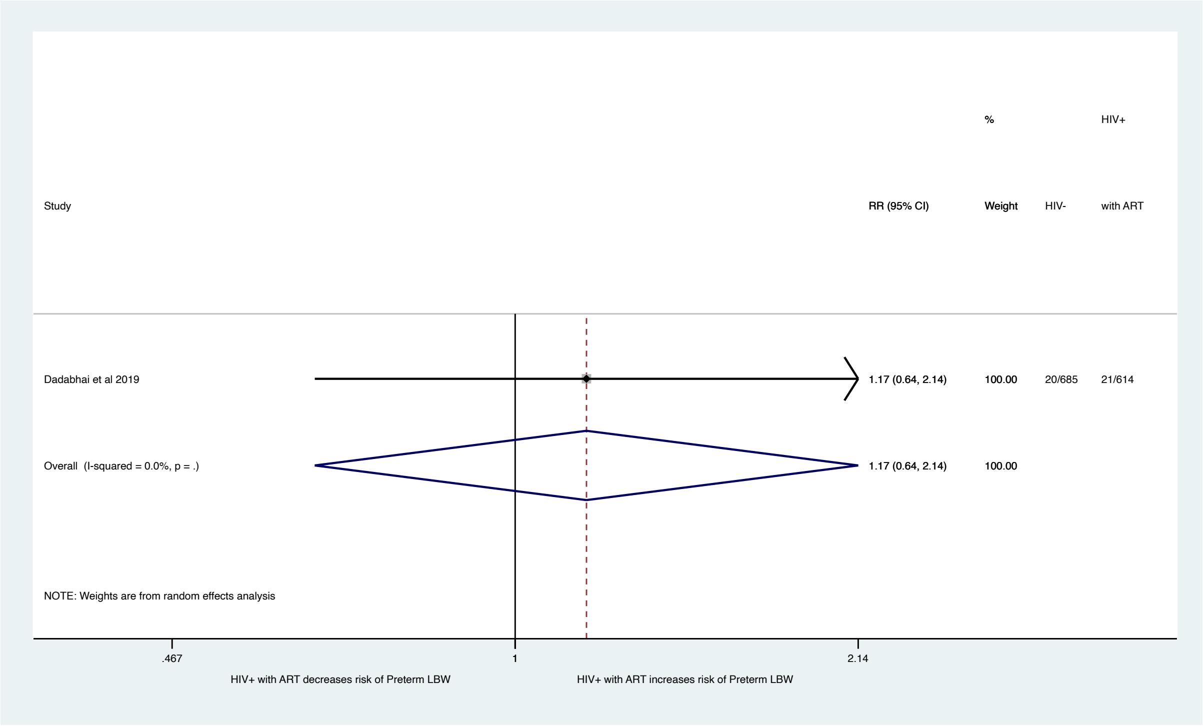

Figure 3.2.7 Preterm low birthweight (preterm LBW) in *w*omen living with HIV receiving ART vs HIV-negative women.

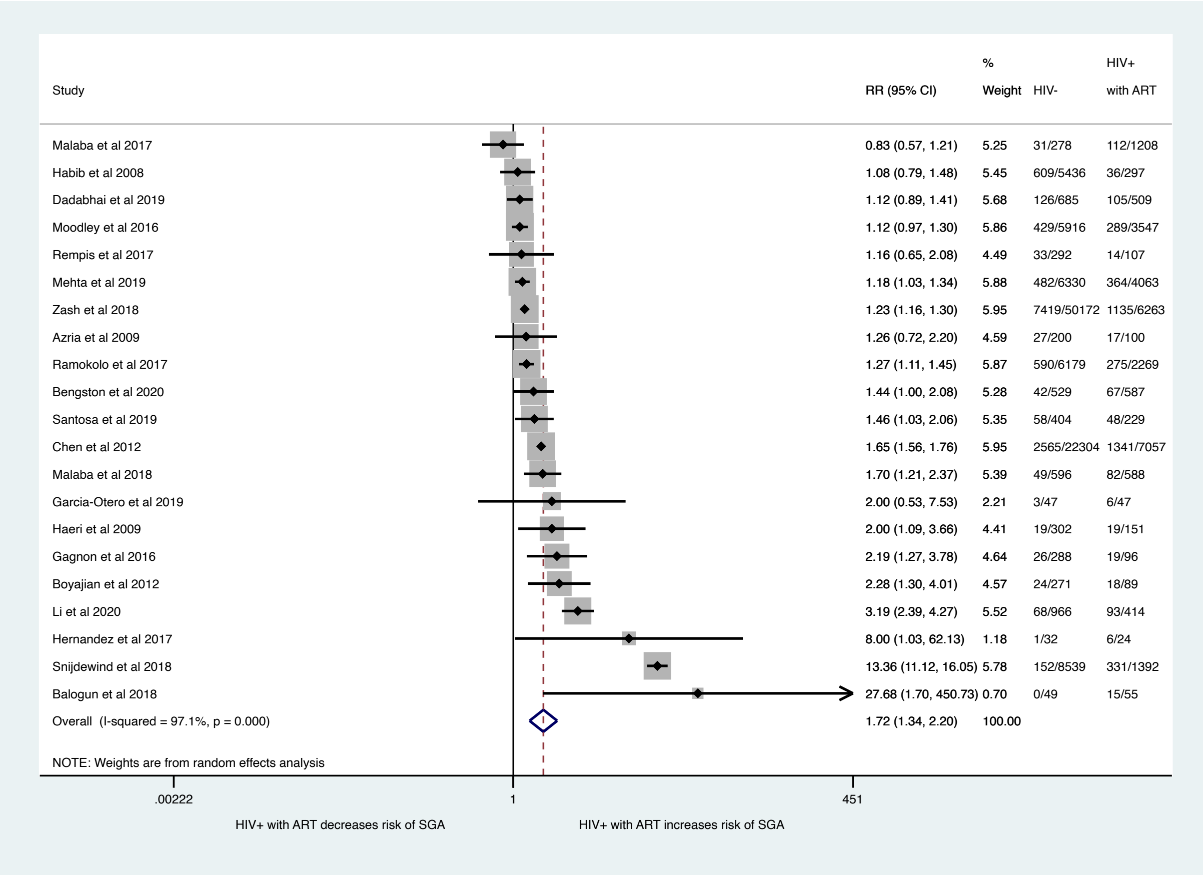

Figure 3.2.8 Small for gestational age (SGA) in women living with HIV receiving ART vs HIV-negative women.

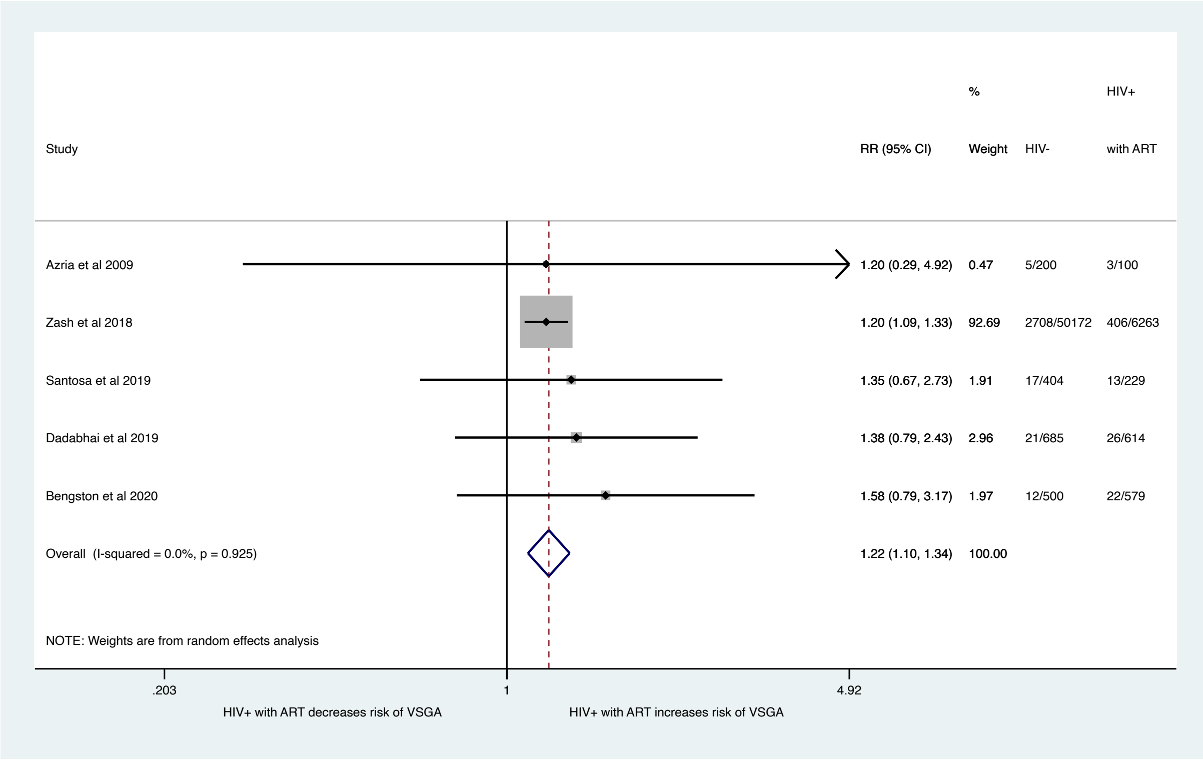

Figure 3.2.9 Very small for gestational age (VSGA) in *w*omen living with HIV receiving ART vs HIV-negative women.

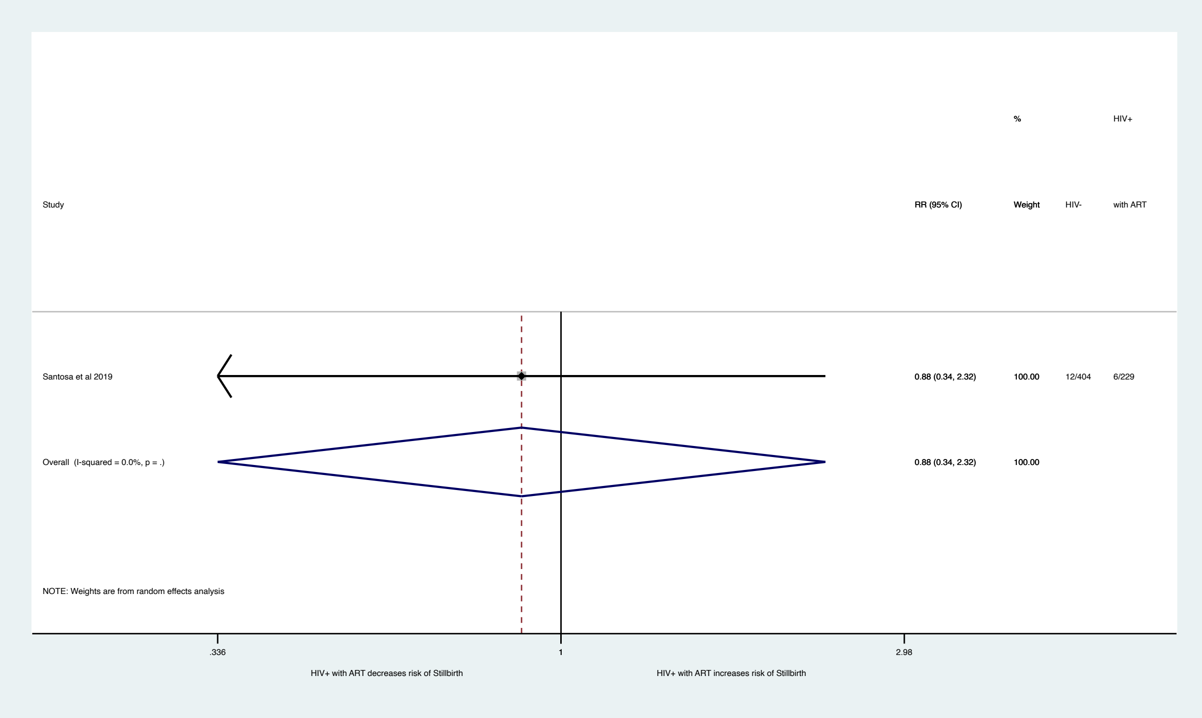

Figure 3.2.10 Stillbirth in *w*omen living with HIV receiving ART vs HIV-negative women.

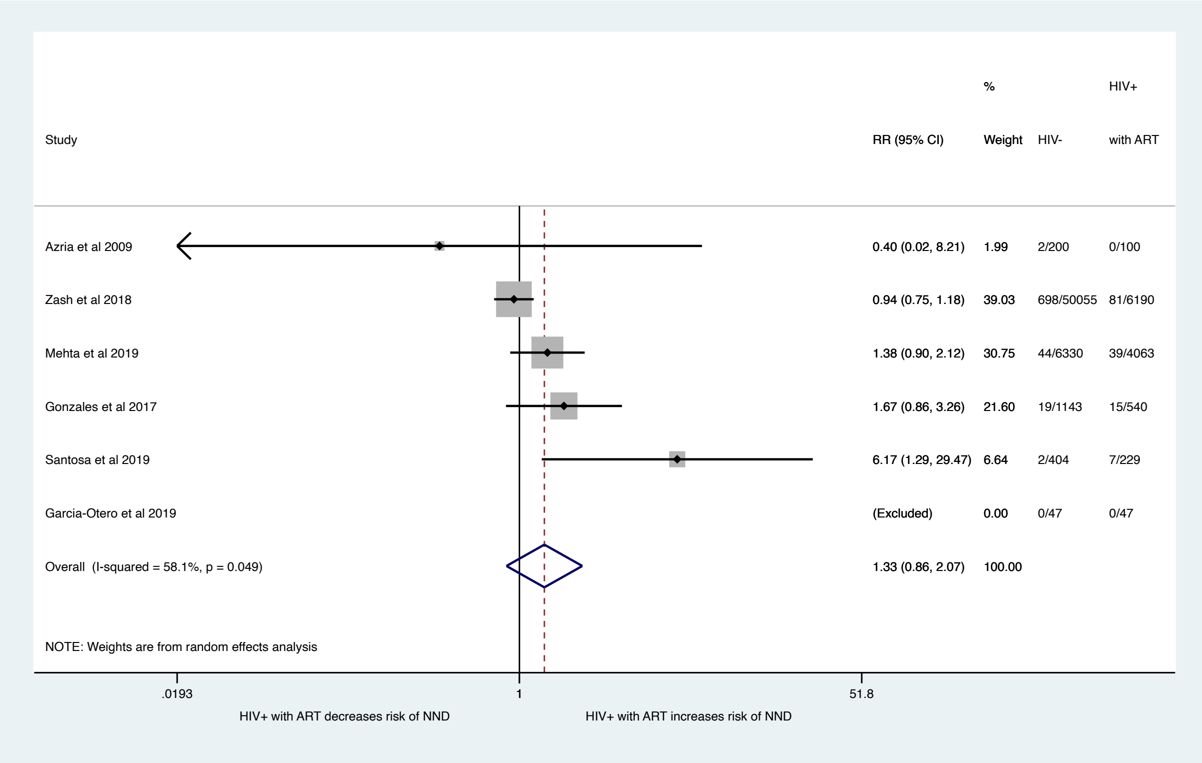

Figure 3.2.11 Neonatal death (NND) in *w*omen living with HIV receiving ART vs HIV-negative women.

Appendix 4
Adjusting for confounders in individual studies

Appendix 4.1
Sensitivity analysis: adjusting for confounders in individual studies comparing women living with HIV receiving ART to HIV-negative women for preterm birth (PTB), very preterm birth (VPTB), spontaneous preterm birth (sPTB), low birthweight (LBW), and very low birthweight (LBW).

Study	ART exposure	Perinatal outcomes									
		PTB		VPTB		sPTB		LBW		VLBW	
		OR/RR (95% CI)	aOR/aRR (95% CI)	OR/RR (95% CI)	aOR/aRR (95% CI)	OR/RR (95% CI)	aOR/aRR (95% CI)	OR/RR (95% CI)	aOR/aRR (95% CI)	OR/RR (95% CI)	aOR/aRR (95% CI)
**Boyajian (2012)**[27]	HIV-	ref	ref					ref	ref		
	HIV+ with ART	1.43 (0.68, 2.97)	1.70 (0.79, 3.66)					2.30 (1.14, 4.60)	2.91 (1.47, 5.78)		
**Dadabhai (2019)**[34]	HIV-	ref	ref					ref	ref		
	HIV+ with ART	1.13 (0.77, 1.65)	1.10 (0.73, 1.65)					1.48 (0.91, 2.42)	1.62 (0.97, 2.71)		
**Gagnon (2016)**[39]	HIV-	ref	ref					ref	ref		
	HIV+ with ART	2.60 (1.30, 5.10)	1.00 (0.30, 3.00)					2.90 (1.40, 6.20)	1.50 (0.40, 5.20)		
**Habib (2008)**[44]	HIV-	ref	ref								
	HIV+ with ART	0.99 (0.66, 1.47)*	0.99 (0.65, 1.49)*								

**Haeri (2009)**[45]	HIV-					ref	ref				
	HIV+ with ART					2.20 (1.16, 4.15)	2.27 (1.20, 4.30)				
**Li (2020)**[53]	HIV-	ref	ref					ref	ref		
	HIV+ with ART	2.54 (1.53, 4.19)	2.37 (1.44,3.89)					4.78 (2.95, 7.85)	4.20 (2.59, 6.82)		
**Lopez (2012)**[55]	HIV-	ref	ref								
	HIV+ with ART	2.60 (1.90, 3.60)	2.50 (1.90, 3.50)								
**Malaba (2017)**[56]	HIV-	ref	ref					ref	ref		
	HIV+ with ART	1.94 (1.33, 2.91)	2.03 (1.33,3.10)					1.62 (1.04, 2.60)	1.47 (0.90, 2.40)		
**Malaba (2018)**[56]	HIV-	ref	ref								
	HIV+ with ART	1.77 (1.21, 2.62)	1.98 (1.12, 3.53)								
**Moodley (2016)**[63]	HIV-	ref	ref					ref	ref		
	HIV+ with ART	1.26 (1.14, 1.40)	1.33 (1.19, 1.48)					1.26 (1.11, 1.43)	1.26 (1.11, 1.44)		
**Santosa (2019)**[71]	HIV-	ref	ref	ref	ref			ref	ref	ref	ref

	HIV+ with ART	1.38<br>(0.89, 2.15)	1.40<br>(0.86, 2.26)	1.26<br>(0.57, 2.80)	1.26<br>(0.53, 3.02)			1.26<br>(0.83, 1.92)	1.15<br>(0.73, 1.82)	1.06<br>(0.43, 2.59)	1.14<br>(0.43, 3.04)
**Zash (2018)**[88]	HIV-	ref	ref	ref	ref						
	HIV+ with ART	1.18<br>(1.12, 1.25)*	1.18<br>(1.12, 1.25)*	1.01<br>(0.88, 1.16)*	1.01<br>(0.88, 1.16)*						

Odds ratio (OR), adjusted odds ratio (aOR), and 95% confidence intervals (95% CIs) of perinatal outcomes in women living with HIV receiving ART vs HIV-negative women reported by studies included in the meta-analysis. Red indicates a significant association and black a non-significant association.
Abbreviations: ART= antiretroviral therapy, HIV= human immunodeficiency virus. *Indicates RR (risk ratio) and aRR (adjusted risk ratio) reported instead of OR/aOR.

Appendix 4.2

Sensitivity analysis: adjusting for confounders in individual studies comparing women living with HIV receiving ART to HIV-negative women for small for gestational age (SGA), very small for gestational age (VSGA), stillbirth, and neonatal death (NND).

Study	ART exposure	Perinatal outcomes							
		SGA		VSGA		Stillbirth		NND	
		OR/RR (95% CI)	aOR/aRR (95% CI)	OR/RR (95% CI)	aOR/aRR (95% CI)	OR/RR (95% CI)	aOR/aRR (95% CI)	OR/RR (95% CI)	aOR/aRR (95% CI)
**Boyajian (2012)**[27]	HIV-	ref	ref						
	HIV+ with ART	2.61 (1.27, 5.33)	2.08 (0.89, 5.25)						
**Dadabhai (2019)**[34]	HIV-	ref	ref						
	HIV+ with ART	1.15 (0.85, 1.55)	1.02 (0.75, 1.41)						
**Gagnon (2016)**[39]	HIV-	ref	ref						
	HIV+ with ART	2.50 (1.30, 4.70)	1.30 (0.50, 3.90)						
**Habib (2008)**[44]	HIV-	ref	ref						
	HIV+ with ART	1.06 (0.76, 1.48)*	1.05 (0.75, 1.48)*						
**Haeri (2009)**[45]	HIV-	ref	ref						
	HIV+ with ART	2.14 (1.03, 4.23)	1.80 (0.89, 3.70)						

**Li (2020)**[53]	HIV-	ref	ref						
	HIV+ with ART	3.82 (2.69, 5.45)	3.26 (2.29, 4.64)						
**Lopez (2012)**[55]	HIV-								
	HIV+ with ART								
**Malaba (2017)**[56]	HIV-	ref	ref						
	HIV+ with ART	0.81 (0.53, 1.28)	0.91 (0.58, 1.43)						
**Malaba (2018)**[56]	HIV-								
	HIV+ with ART								
**Moodley (2016)**[63]	HIV-	ref	ref						
	HIV+ with ART	1.15 (0.98, 1.33)	1.15 (0.98, 1.35)						
**Santosa (2019)**[71]	HIV-	ref	ref	ref	ref	ref	ref	ref	ref
	HIV+ with ART	1.55 (1.01, 2.37)	1.45 (0.91, 2.33)	1.33 (0.63, 2.81)	1.50 (0.66, 3.44)	0.88 (0.33, 2.37)	0.77 (0.24, 2.41)	6.15 (1.27, 29.88)	7.82 (1.32, 46.42)
**Zash (2018)**[88]	HIV-	ref	ref	ref	ref	ref	ref	ref	ref

	HIV+ with ART	1.23<br>(1.17, 1.31)*	1.30<br>(1.23,1.38)*	1.21<br>(1.09,1.34)*	1.28<br>(1.16, 1.42)*	1.10<br>(0.92, 1.30)*	1.08<br>(0.91, 1.29)*	0.94<br>(0.75, 1.18)*	0.92<br>(0.73, 1.17)*

Odds ratio (OR), adjusted odds ratio (aOR), and 95% confidence intervals (95% CIs) of perinatal outcomes in women living with HIV receiving ART vs HIV-negative women reported by studies included in the meta-analysis. Red indicates a significant association and black a non-significant association.
Abbreviations: ART= antiretroviral therapy, HIV= human immunodeficiency virus. *Indicates RR (risk ratio) and aRR (adjusted risk ratio) reported instead of OR/aOR.

Appendix 4.3

Sensitivity analysis: adjusting for confounders in individual studies comparing women living with HIV receiving ART to women living with HIV without ART for preterm birth (PTB), very preterm birth (VPTB), spontaneous preterm birth (sPTB), low birthweight (LBW), and very low birthweight (LBW).

**Study**	**ART exposure**	**Perinatal outcomes**					
		**PTB**		**VPTB**		**LBW**	
		OR/RR (95% CI)	aOR/aRR (95% CI)	OR/RR (95% CI)	aOR/aRR (95% CI)	OR/RR (95% CI)	aOR/aRR (95% CI)
**Chagomerana (2017)**[29]	HIV+ without ART	1.05 (0.80, 1.38)*	1.14 (0.84, 1.55)*	2.02 (1.24, 3.30)*	2.33 (1.39, 3.92)*		
	HIV+ with ART	ref	ref	ref	ref		
**Cotter (2006)**[33]	HIV+ without ART					ref	ref
	HIV+ with ART					0.90 (0.60-1.20)	0.90 (0.50-1.70)

Odds ratio (OR), adjusted odds ratio (aOR), and 95% confidence intervals (95% CIs) of perinatal outcomes in women living with HIV receiving ART vs ART-naïve women living with HIV reported by studies included in the meta-analysis. Red indicates a significant association and black a non-significant association.

Abbreviations: ART= antiretroviral therapy, HIV= human immunodeficiency virus. *Indicates RR (risk ratio) and aRR (adjusted risk ratio) reported instead of OR/aOR.
